# Developing a class of dual atom materials for multifunctional catalytic reactions

**DOI:** 10.1038/s41467-023-42756-8

**Published:** 2023-11-08

**Authors:** Xingkun Wang, Liangliang Xu, Cheng Li, Canhui Zhang, Hanxu Yao, Ren Xu, Peixin Cui, Xusheng Zheng, Meng Gu, Jinwoo Lee, Heqing Jiang, Minghua Huang

**Affiliations:** 1 School of Materials Science and Engineering, Ocean University of China, Qingdao, China; 2 Qingdao Key Laboratory of Functional Membrane Material and Membrane Technology, Qingdao Institute of Bioenergy and Bioprocess Technology, Chinese Academy of Sciences, Qingdao, China; 3 Shandong Energy Institute, Qingdao, China; 4 Qingdao New Energy Shandong Laboratory, Qingdao, China; 5 Department of Chemical and Biomolecular Engineering, Korea Advanced Institute of Science and Technology (KAIST), Yuseong-Gu, Daejeon Republic of Korea; 6 Eastern Institute for Advanced Study, Eastern Institute of Technology, Ningbo, Zhejiang PR China; 7 Department of Materials Science and Engineering, Southern University of Science and Technology, Shenzhen, China; 8 School of Physics and Astronomy, University of Birmingham, Birmingham, UK; 9 Key Laboratory of Soil Environment and Pollution Remediation, Institute of Soil Science, Chinese Academy of Sciences, Nanjing, China; 10 National Synchrotron Radiation Laboratory (NSRL), University of Science and Technology of China, Hefei, China

**Keywords:** Electrocatalysis, Fuel cells, Cheminformatics

## Abstract

Dual atom catalysts, bridging single atom and metal/alloy nanoparticle catalysts, offer more opportunities to enhance the kinetics and multifunctional performance of oxygen reduction/evolution and hydrogen evolution reactions. However, the rational design of efficient multifunctional dual atom catalysts remains a blind area and is challenging. In this study, we achieved controllable regulation from Co nanoparticles to CoN_4_ single atoms to Co_2_N_5_ dual atoms using an atomization and sintering strategy via an N-stripping and thermal-migrating process. More importantly, this strategy could be extended to the fabrication of 22 distinct dual atom catalysts. In particular, the Co_2_N_5_ dual atom with tailored spin states could achieve ideally balanced adsorption/desorption of intermediates, thus realizing superior multifunctional activity. In addition, it endows Zn-air batteries with long-term stability for 800 h, allows water splitting to continuously operate for 1000 h, and can enable solar-powered water splitting systems with uninterrupted large-scale hydrogen production throughout day and night. This universal and scalable strategy provides opportunities for the controlled design of efficient multifunctional dual atom catalysts in energy conversion technologies.

## Abstract (web summary)

This work developed a class of dual atom materials that can act as efficient and stable catalysts for multifunctional catalytic reactions in an uninterrupted water splitting system.

## Introduction

Vigorously developing the green hydrogen economy would bring great benefits for decarbonization in energy sectors^1–4^. Water splitting systems (WSSs), realized using renewable energy (e.g., solar energy) as the power and rechargeable batteries (e.g., metal-air batteries) as electricity storage, have been widely recognized as sustainable and CO_2_-free energy devices for efficient and uninterrupted hydrogen (H_2_) production^5–7^. Three core half-reactions of WSSs, namely the oxygen reduction reaction (ORR), oxygen evolution reaction (OER), and hydrogen evolution reaction (HER), are all subject to complicated multi-step proton-coupled electron transfer process, leaving the challenging issues of sluggish kinetics and high overpotentials^8–14^. To date, the benchmark catalysts for improving these reaction efficiencies are precious-metal-based materials (i.e., RuO_2_/IrO_2_ only for OER, Pt/C only for ORR/HER), but their shortcomings, such as the high cost, scarcity, poor stability, and single-functionality, greatly restrict the large-scale application for series WSSs^15–18^. Although significant efforts toward these core half-reactions have been established in the widely acclaimed single atom catalyst (SAC) featuring minimum particle size, maximum atomic utilization, and well-defined metal-N_4_ (M-N_4_) active sites, their multifunctionality and sluggish ORR/OER/HER kinetics still remain unsolved^19–24^. This arises from the obvious demerit of SAC, that is, only one kind of specific isolated active site, which makes it difficult to break the linear scaling relations between the adsorption/desorption toward complicated multi-intermediates and improve the catalytic ORR/OER/HER activities to the optimal level^25–27^. Dual atom catalysts (DACs) with adjacent dual atom sites have surfaced as a new research frontier that could bridge the SAC and metal/alloy nanoparticle catalysts, which endow DACs with integrated merits including high metal atom loading, more sophisticated and flexible active sites, easily modulated electronic structure, and the synergetic effects between two adjacent active sites^26,28–34^. Benefiting from these features, DACs offers more prospects toward conquering the challenges and limitations faced by SACs via synergistically adjusting the adsorption/desorption behaviors and activation of intermediates, thus accomplishing accelerated reaction kinetics and efficient multifunctional ORR/OER/HER performance^26,35,36^. Unfortunately, the rational design of highly efficient and robust DACs with multifunctionality is still in a blind area and with numerous challenges due to the lack of advanced fundamental knowledge of formation mechanisms for DACs.

In this study, a nanoparticle-to-single-atom-to-dual-atom (NP-to-SA-to-DA) atomization and sintering strategy was developed. We realized the controllable adjustment of the existing configuration states of Co species at the atomic level, yielding Co nanoparticles, CoN_4_ single atoms, and Co_2_N_5_ dual atoms on N-doped hollow carbon spheres (termed Co_NP_/HCS-900, Co_SA_-N-HCS-900, and Co_2_-N-HCS-900, respectively). This special design strategy allowed the investigation of the formation mechanism from NP to SA to DA. Density functional theory and experimental results demonstrated that Co atoms could be gradually stripped from the nanoparticles and trapped by N anchoring sites to form CoN_4_ single atoms, and then spontaneously sintered into Co_2_N_5_ dual atoms via thermal migration. Moreover, it was found that the spin state of Co atoms can be harmonized from NP to SA to DA, in which the Co_2_N_5_ dual atom with a low spin state can achieve ideally balanced adsorption/desorption of O* and H* intermediates. As expected, the Co_2_-N-HCS-900 affords the boosted multifunctional ORR/OER/HER activity, which endowed Zn-air batteries (ZABs) with excellent cycling charge–discharge stability over 800 h and enabled water splitting to operate for over 1000 h. The highly efficient and durable solar-powered WSS constructed using only Co_2_-N-HCS-900 ensured uninterrupted large-scale H_2_ production throughout the day and night for over 48 h. Most strikingly, this NP-to-SA-to-DA atomization and sintering strategy can be broadened to prepare 22 types of *s*-, *p*-, and *d*-block metal dual atom catalysts. This work provides a systematic study on the formation mechanisms and catalytic activities of nanoparticle, single atom, and dual atom catalysts, undoubtedly leading to an upsurge in the rational design of efficient and stable dual atom catalysts for applications in energy conversion technologies.

## Results and discussion

### Catalyst design concept

**Fig. 1:**
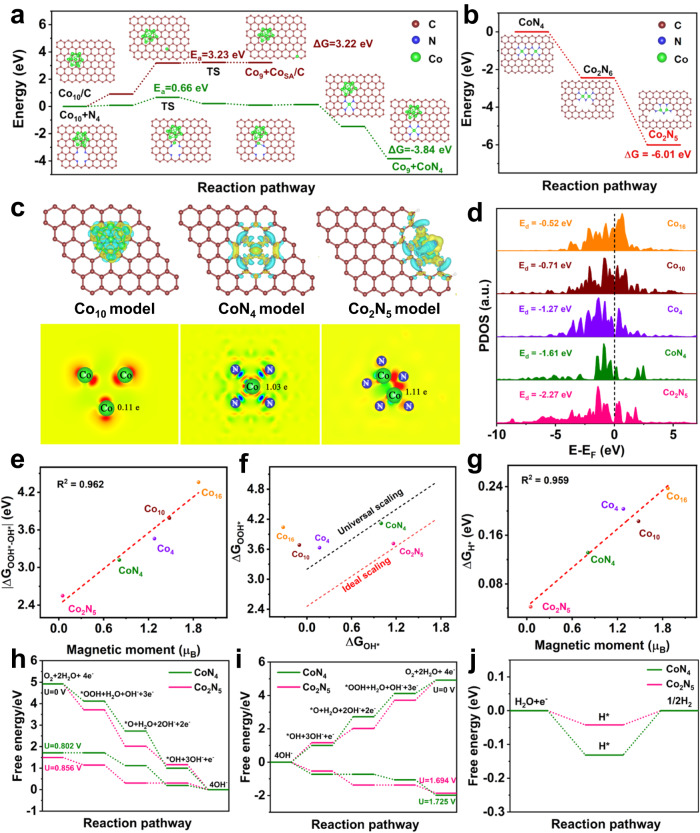
DFT calculation. **a** Calculated relative energies along the stretching pathway of the Co atom from Co_10_ to Co_SA_/C or CoN_4_ model by CI-NEB, **b** Calculated relative energies of CoN_4_, Co_2_N_6_, and Co_2_N_5_ models. **c** Charge density difference and Bader charge diagrams; **d** PDOS; **e** linear correlation between magnetic moment and |ΔG_OOH*-OH*_|; **f** the ΔG_OOH*_ to ΔG_OH*_ scaling for Co_16_, Co_10_, Co_4_, CoN_4_, and Co_2_N_5_ models relative to the universal and ideal scaling lines; and **g** linear correlation between the magnetic moment and ΔG_H*_. Free energy diagrams of CoN_4_ and Co_2_N_5_ models for **h** ORR, **i** OER, and **j** HER.

Density functional theory (DFT) calculations were conducted to reveal the structural transformation mechanism from nanoparticles to single atoms and then to dual atoms (NP-to-SA-to-DA) by taking Co_10_ nanoparticles as examples^37,38^. First, the atomization process (Fig. 1a) was investigated from the Co_10_ nanoparticles to two types of Co single atoms with different coordination of C (named Co_SA_/C) and N atoms (named CoN_4_). It can be seen that the formation of Co_SA_/C from Co_10_ nanoparticle decomposition requires overcoming a very high kinetic barrier of 3.23 eV with a large endothermicity of 3.22 eV, indicating that Co_10_ nanoparticles might be the main form of existence. The formation of CoN_4_ from Co_10_ nanoparticles decomposition requires a relatively low kinetic barrier of 0.66 eV to be overcome but with a large exothermicity of 3.84 eV, manifesting that the introduction of N elements can promote the thermal atomization from Co_10_ nanoparticles to CoN_4_ single atoms. Additionally, the atomization processes of the Co_16_ and Co_4_ models were investigated. Figure S1 and S2 show that the formation of Co_SA_/C from the decomposition of Co_4_ and Co_16_ nanoparticles requires overcoming a very high kinetic barrier of 3.97 and 3.65 eV, respectively, which is higher than that of the decomposition of Co_10_ nanoparticles (3.23 eV). Moreover, the formation of CoN_4_ from Co_10_ nanoparticles decomposition requires a relatively low kinetic barrier (0.66 eV) to be overcome, lower than those for the formation of CoN_4_ from the decomposition of Co_4_ (1.50 eV) and Co_16_ nanoparticles (1.30 eV). This implies that the transformation from Co_10_ nanoparticles to single atoms can be achieved more easily. Figure 1b displays that the transforming processes, from two neighboring CoN_4_ single atoms to edge-adjacent Co_2_N_6_ and then to the Co_2_N_5_ dual atom, were exothermic with the energy of 2.44 and 3.57 eV, respectively. Again, the processes from two randomly dispersed CoN_4_ single atoms separated by several carbon atoms to edge-adjacent Co_2_N_6_ and then to Co_2_N_5_ dual atoms were exothermic (Figure S3), further confirming that randomly dispersed CoN_4_ single atoms would spontaneously be sintered through the thermal migration process. Because the Co_2_N_5_ dual atom exhibited larger exothermicity than the Co_2_N_6_ dual atom, the former was more stable and dominantly formed during the sintering process. These results indicate that Co atoms could be gradually stripped from the Co_10_ nanoparticles to form CoN_4_ single atoms trapped by the anchoring sites of N, and then spontaneously sintered into Co_2_N_5_ dual atoms via thermal migration. Moreover, this NP-to-SA-to-DA atomization and sintering strategy can be broadened to the research scope of transforming conventional Al, Ca, Cr, Mn, Fe, Ni, Cu, Zn, Ru, Sb, Ce, Bi, and their alloys (taking the FeNi alloy as an example, Figure S4–S16).

Next, we investigated the electronic structures of Co_16_ nanoparticle, Co_10_ nanoparticle, Co_4_ nanoparticle, CoN_4_ single atom, and Co_2_N_5_ dual atom models (Figure S17). The charge density difference and Bader charge results shown in Fig. 1c confirm that the Co atoms of the Co_2_N_5_ dual atoms possess a higher charge of 1.11 e than those of the CoN_4_ single atoms (1.03 e) and Co_10_ nanoparticles (0.11 e). As shown in the projected density of states (PDOS) diagram (Figure S18), the electrons of Co-3*d* orbitals are asymmetrically arranged in the spin channels for Co_16_ nanoparticles, Co_10_ nanoparticles, Co_4_ nanoparticles, CoN_4_ single atoms, and Co_2_N_5_ dual atoms, exhibiting magnetic moments of 1.87, 1.48, 1.28, 0.81, and 0.05 *μ*_B_, respectively. The decreased spin magnetic moment from NP to SA to DA mainly results from the redistribution of electrons of the Co-3*d* orbital triggered by the energy level rearrangement, which gives rise to the increased filling degree of ${d}_{{z}^{2}}$ orbitals and induces the weakened adsorption of reaction intermediates (OOH*, O*, and OH* toward ORR/OER, H* toward HER), thus boosting the catalytic activities. Further support for this phenomenon can be demonstrated by the downshift of the *d*-band center of Co-3*d* orbitals from Co_16_ nanoparticles to Co_10_ nanoparticles to Co_4_ nanoparticles to CoN_4_ single atoms, to Co_2_N_5_ dual atoms, accompanied with the corresponding values of −0.52, −0.71, −1.27, −1.61, and −2.27 eV (Fig. 1d).

We also investigated the Gibbs free energies for the ORR/OER/HER of the Co_16_ nanoparticle, Co_10_ nanoparticle, Co_4_ nanoparticle, CoN_4_ single atom, and Co_2_N_5_ dual atom models to obtain a deeper understanding of the catalytic mechanism and origin of superior catalytic activities. The Gibbs free energy difference between ΔG_OOH*_ and ΔG_OH*_ ( | ΔG_OOH*-OH*_|) can serve as an important ORR/OER reaction descriptor, with an ideal value of 2.46 eV^39–41^. The |ΔG_OOH*-OH*_| is significantly restricted by the adsorption affinity of O*, in which either too strong or too weak adsorption of O* could result in the increased value of |ΔG_OOH*-OH*_ | ^42^. As shown in Fig. 1e, g, and S18, the spin magnetic moment presents a positive and quasi-linear correlation (R^2^ = 0.962) with the |ΔG_OOH*-OH*_|, in the positive sequence from Co_2_N_5_ dual atoms (0.05 *μ*_B_, 2.55 eV) to CoN_4_ single atoms (0.81 *μ*_B_, 3.12 eV) to Co_4_ nanoparticles (1.28 *μ*_B_, 3.46 eV) to Co_10_ nanoparticles (1.48 *μ*_B_, 3.79 eV) and to Co_16_ nanoparticles (1.87 *μ*_B_, 4.36 eV). A universal ΔG_OOH*_ to ΔG_OH*_ scaling relation with an average |ΔG_OOH*-OH*_| value of 3.2 eV has been established for most conventional catalysts, whereas the ideal ΔG_OOH*_ to ΔG_OH*_ scaling relation possesses an average |ΔG_OOH*-OH*_| value of 2.46 eV for ideal catalysts (Fig. 1f)^39,40^. The ΔG_OOH*_ to ΔG_OH*_ coordinate point of the Co_2_N_5_ dual atom is located at the ideal scaling relations accompanied with the |ΔG_OOH*-OH*_| value of 2.55 eV, which is very close to the 2.46 eV for ideal catalysts toward ORR/OER. This indicates that the energy of O* intermediate adsorption/desorption for Co_2_N_5_ was the most appropriate. The possible reason for this phenomenon is that the Co_2_N_5_ dual atom with the decreased spin magnetic moment can break the universal ΔG_OOH*_ to ΔG_OH*_ scaling relation, thus achieving the ideal balanced O* adsorption. This phenomenon is further supported by the moderate O* adsorption energy (1.89 eV, Figure S19) of the Co_2_N_5_ dual atom model among three investigated models, indicating that it could achieve optimized O* adsorption/desorption, thus boosting the ORR/OER activities. To determine the correlation between the spin configuration and the free energy of O*, the crystal orbital Hamilton population (COHP) was calculated to compare the bonding character of O* absorbed onto the Co_10_, CoN_4_, and Co_2_N_5_ models. Positive and negative COHP are due to bonding and antibonding states, respectively. The Co-O bonding strength can be evaluated using the integrated COHP (ICOHP) values, which could quantitatively describe the *d*-*p* hybridization strength. As depicted in Figure S20, the ICOHP values were found to be −0.22 for Co_10_, −0.47 for CoN_4_, and −0.45 for Co_2_N_5_. The intermediate ICOHP value of the three investigated models confirms the moderated Co-O affinity in the Co_2_N_5_ model, suggesting optimal O* adsorption/desorption, enhancing the ORR/OER activities. In Figure S21, a pronounced antibonding state for the Co_2_N_5_ model emerges at the Fermi level compared to the other models, implying greater electron transfer from the Co-3*d* orbital to the vacant O-2*p* orbital. This leads to reduced reaction activation energy and improved catalyst conductivity with minimal ohmic loss, enhancing catalytic activity^43,44^. Regarding HER, a negative and quasi-linear correlation exists between the spin magnetic moment and the Gibbs free energy of H* (ΔG_H*_). Specifically, ΔG_H*_ decreases linearly (R^2^ = 0.959) with the increased spin magnetic moment (Fig. 1g). Among the studied models, the Co_2_N_5_ dual atom possesses the highest ΔG_H*_ of −0.04 eV (close to the ideal value of 0 eV), demonstrating that its low spin state facilitates the moderate adsorption and desorption of the H* intermediate. Moreover, the highest H* adsorption energy of −0.21 eV was obtained among the three investigated models (Figure S22), again validating that it affords moderate adsorption and desorption of H*, endowing excellent HER activities.

Figure 1h–j and S23–27 depict the optimized atomic configurations and Gibbs free energies of the Co_16_ nanoparticle, Co_10_ nanoparticle, Co_4_ nanoparticle, CoN_4_ single atom, and Co_2_N_5_ dual atom models bonded with the adsorption of reaction intermediates for catalyzing the ORR/OER/HER. For ORR (Fig. 1h and S25), the Co_2_N_5_ dual atom affords a higher thermodynamic limiting potential of 0.856 V than both the CoN_4_ single atom (0.802 V) and Co_10_ nanoparticles (−0.102 V), highlighting its good ORR activity. As shown in Fig. 1i and S26, the lowest thermodynamic limiting potential of 1.694 V toward the OER was obtained for the Co_2_N_5_ dual atom among the investigated models, indicating that it shows the lowest overpotential and superior OER efficiency. Figure 1j and S27 show the Gibbs free energy of H* intermediates toward the HER across the models, where the Co_2_N_5_ dual atom provides an adsorption energy value of H* of −0.04 eV, closest to 0 eV, indicating its superior HER performance. Based on DFT analyses, the Co_2_N_5_ dual atom has a tailored spin state that promotes the moderated and balanced adsorption and desorption of reaction intermediates toward the ORR/OER/HER, enhancing its trifunctional performance^45,46^.

### Synthesis and structural characterization

**Fig. 2:**
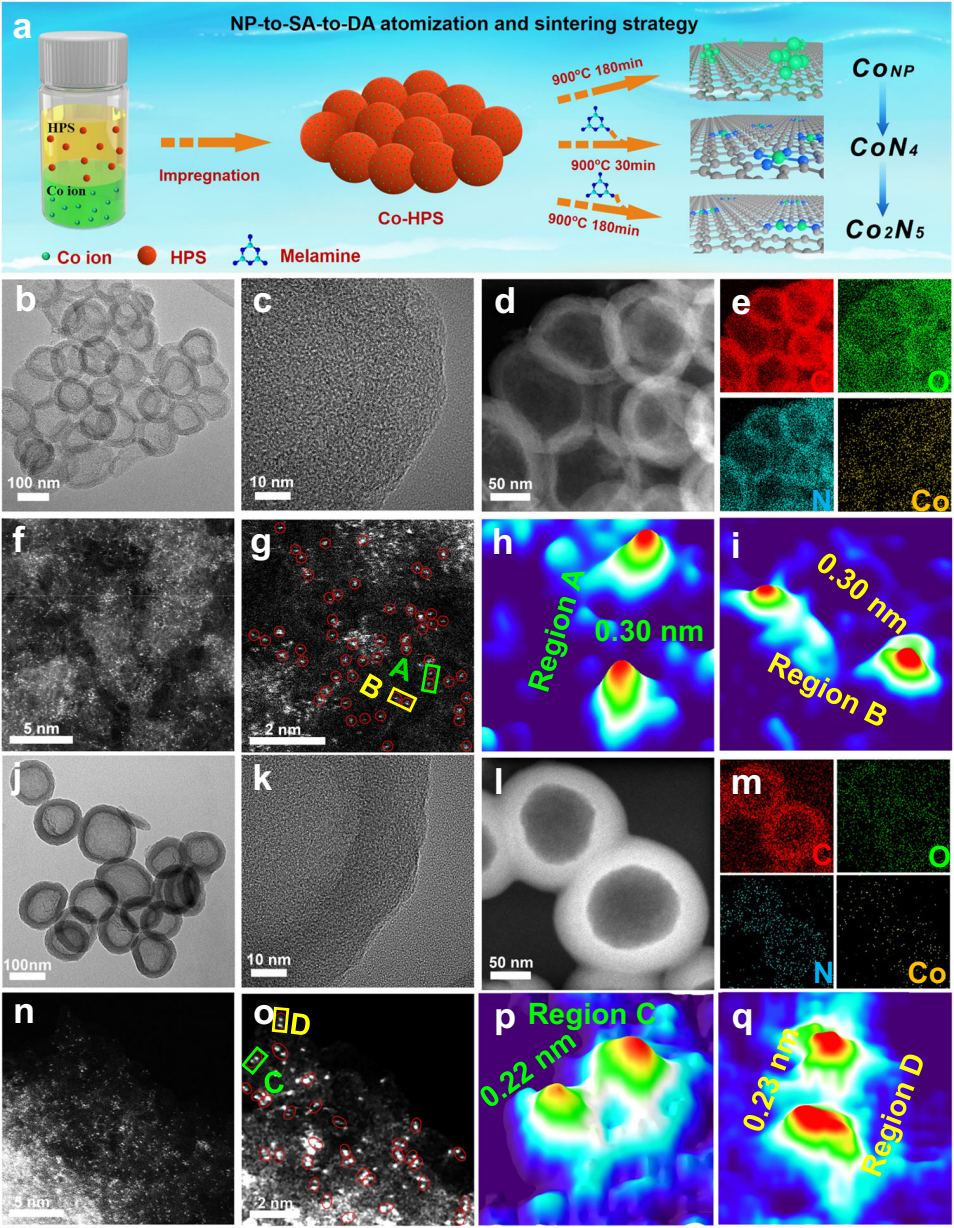
Structural characterizations. **a** Schematic diagram of the NP-to-SA-to-DA atomization and sintering strategy. **b** TEM, **c** HRTEM, **d** HAADF-STEM, and **e** C, O, N, Co elemental mapping images; **f**, **g** AC HAADF-STEM images (Co single atoms marked by red circles); **h**, **i** 3D atom-overlapping Gaussian-function fitting map in **g** for Co_SA_-N-HCS-900. **j** TEM, **k** HRTEM, **l** HAADF-STEM, and **m** C, O, N, Co elemental mapping images; **n**, **o** AC HAADF-STEM images (Co dual atoms marked by red circles); **p**, **q** 3D atom-overlapping Gaussian-function fitting map in **o** for Co_2_-N-HCS-900.

Driven by DFT analysis, a series of catalysts were prepared, including Co_NP_/HCS-900 with aggregated Co nanoparticles, Co_SA_-N-HCS-900 with CoN_4_ single atoms, and Co_2_-N-HCS-900 with paired Co_2_N_5_ dual atoms (Fig. 2a). A facile double-solvent impregnation method was used to prepare the Co-hollow polymer spheres (Co-HPS) precursor. Then, Co_NP_/HCS-900 was obtained by directly pyrolyzing the Co-HPS, and the Co_NP_/N-HCS-300 were synthesized by pyrolyzing the Co-HPS with melamine at 300 °C. Further, Co_SA_-N-HCS-900 can be synthesized when the melamine was added in the above pyrolysis process since the melamine can be decomposed at high temperatures (>400 °C) and serve as the N agent that is generally coordinated with Co atoms to form Co-Nx moieties for promoting the Co nanoparticles atomization^47^. By effectively revising the calcination time, the single Co atoms could couple with each other and sinter to form Co_2_-N-HCS-900 with the paired Co_2_N_5_ dual atom dimer via thermal migration. The X-ray diffraction (XRD) patterns in Figure S28 show that only Co_NP_/HCS-900 has a diffraction peak indexing crystalline Co nanoparticle, whereas no peak can be observed for either Co_SA_-N-HCS-900 or Co_2_-N-HCS-900. Further support for this phenomenon can be provided by their corresponding transmission electron microscope (TEM) and high-resolution TEM (HRTEM) images. As shown in Figure S29, numerous nanoparticles, accompanied by a lattice distance of 0.203 nm, indexing the (111) facet of metallic Co, were uniformly anchored on the hollow carbon spheres of Co_NP_/HCS-900. No obvious Co nanoparticles are observed in either Co_SA_-N-HCS-900 (Fig. 2b–i) or Co_2_-N-HCS-900 (Fig. 2j–q). Moreover, both Co_SA_-N-HCS-900 and Co_2_-N-HCS-900 exhibited ring-like diffraction in the selected area electron diffraction patterns (Figure S30 and S31) and a uniform distribution of C, N, O, and Co elements in the associated elemental mapping profiles (Figs. 2d–e, l–m), indicating the atomic dispersion of Co species on both Co_SA_-N-HCS-900 and Co_2_-N-HCS-900.

**Fig. 3:**
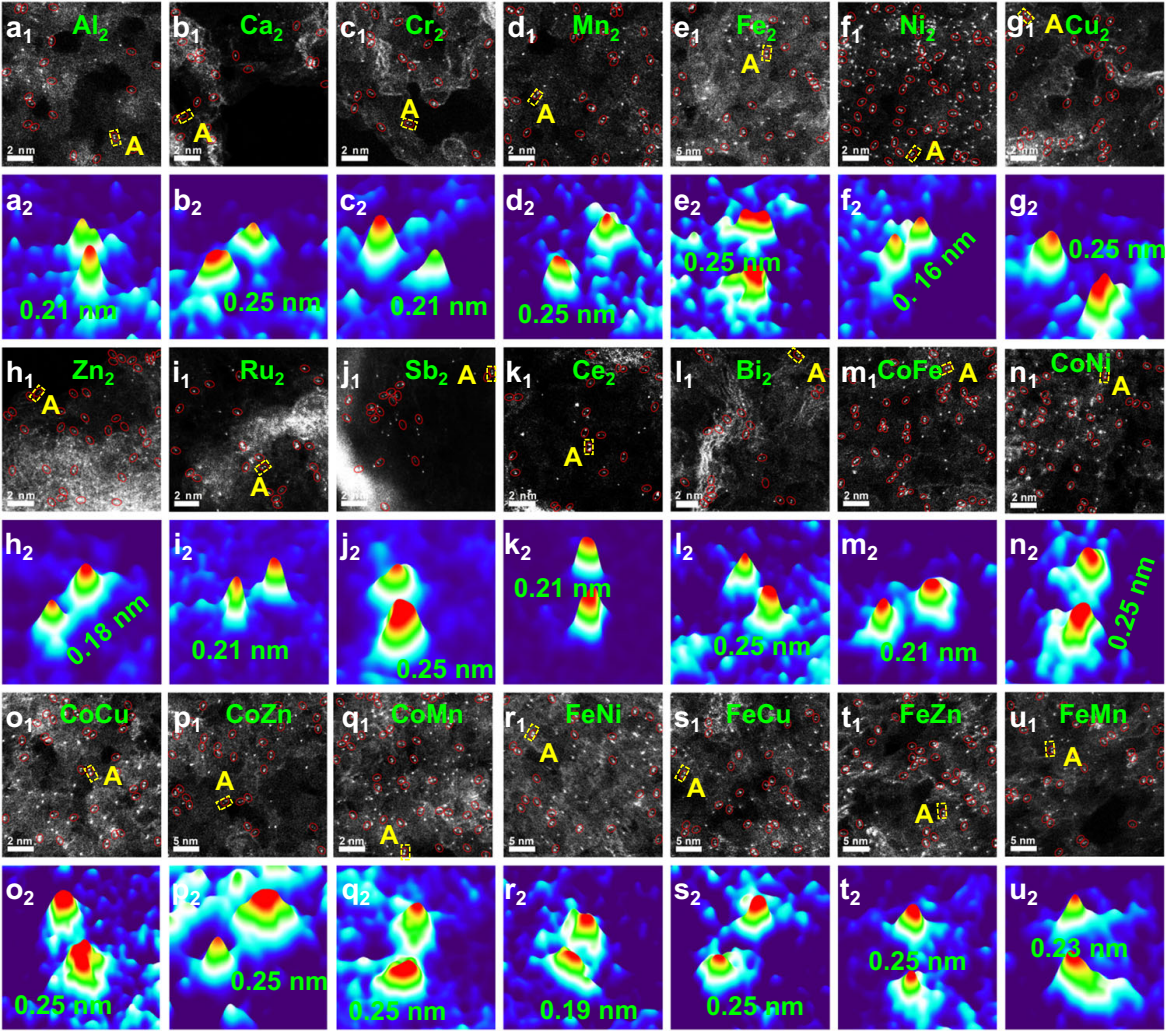
Structural characterizations of 21 types of *s*-, *p*-, and *d*-block dual atom catalysts. AC HAADF-STEM and corresponding 3D atom-overlapping Gaussian-function fitting map of region A for **a** Al_2_-N-HCS-900, **b** Ca_2_-N-HCS-900, **c** Cr_2_-N-HCS-900, **d** Mn_2_-N-HCS-900, **e** Fe_2_-N-HCS-900, **f** Ni_2_-N-HCS-900, **g** Cu_2_-N-HCS-900, **h** Zn_2_-N-HCS-900, **i** Ru_2_-N-HCS-900, **j** Sb_2_-N-HCS-900, **k** Ce_2_-N-HCS-900, **l** Bi_2_-N-HCS-900, **m** CoFe-N-HCS-900, **n** CoNi-N-HCS-900, **o** CoCu-N-HCS-900, **p** CoZn-N-HCS-900, **q** CoMn-N-HCS-900, **r** FeNi-N-HCS-900, **s** FeCu-N-HCS-900, **t** FeZn-N-HCS-900, and **u** FeMn-N-HCS-900.

Aberration-corrected high-angle annular dark-field scanning transmission electron microscopy (AC HAADF-STEM) was used to investigate the atomic states of the Co species in Co_NP_/N-HCS-300, Co_SA_-N-HCS-900, and Co_2_-N-HCS-900. As shown in Figure S32, several aggregated nanoparticles (marked with red cycles) were captured on the carbon substrate, indicating that some Co nanoparticles had surfaced on Co_NP_/N-HCS-300. The AC HAADF-STEM images of Co_SA_-N-HCS-900 (Fig. 2f–g) and Co_2_-N-HCS-900 (Fig. 2n–o) displays numerous bright dots (marked with red circles) homogeneously dispersed on the carbon skeleton, which indicate Co atoms due to the difference in Z-contrast between the heavier Co and the lighter C and N. The projected distance between two adjacent Co atoms in Co_SA_-N-HCS-900 is mainly distributed in the range of 0.30–0.50 nm (Fig. 2h–i and S33), indicating that the Co species mainly exist as single atoms. As for Co_2_-N-HCS-900 (Fig. 2o), a large proportion of Co atoms are adjacent to each other and presented in the form of dual Co atoms, with the Co-Co distance ranging from 0.12 nm to 0.25 nm (Fig. 2p–q and S34). This verifies the presence of paired Co_2_ dual atom dimers. Figure S35 reveals numerous pores (marked by green circles) surrounding the paired Co_2_ dual atom dimers, indicating that the dimers might be positioned at the edge of the carbon framework^28,48,49^. This suggests that by adjusting calcination durations, single Co atoms can merge to create edge-adjacent paired Co_2_ dual atom dimers. Additionally, this NP-to-SA-to-DA atomization and sintering strategy can be adopted for the generalized synthesis of 21 types of edge-adjacent paired *s*-, *p*-, and *d*-block M_2_ dual atom structures, including edge-adjacent Al_2_, Ca_2_, Cr_2_, Mn_2_, Fe_2_, Ni_2_, Cu_2_, Zn_2_, Ru_2_, Sb_2_, Ce_2_, Bi_2_, CoFe, CoNi, CoCu, CoZn, CoMn, FeNi, FeCu, FeZn, and FeMn dual atoms on N-HCS, which were confirmed by the AC HAADF-STEM and the corresponding 3D atom-overlapping Gaussian-function fitting map analysis (Fig. 3 and S36).

### Atomic coordination structure analysis

**Fig. 4:**
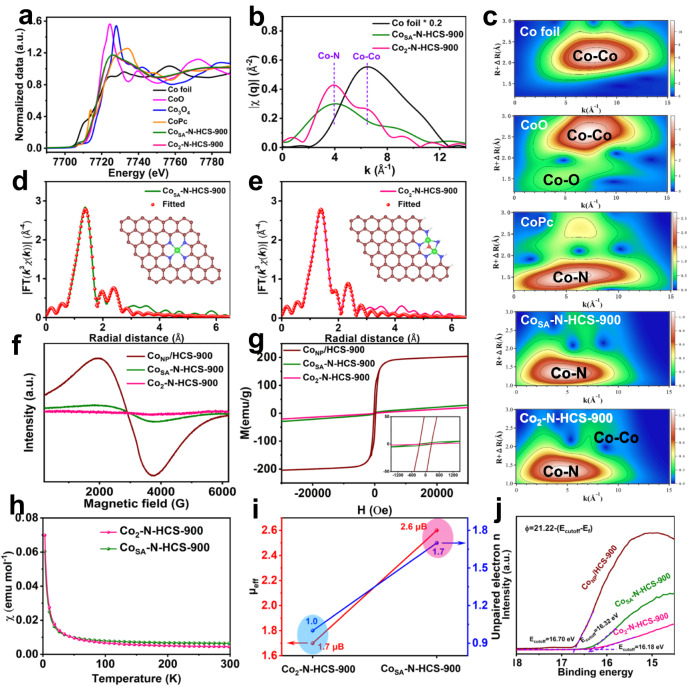
Local structural characterizations and electron spin-state analysis. **a** XANES spectra, **b** q-space magnitude comparisons, and **c** WT-EXAFS at Co K-edge of Co foil, CoO, CoPc, Co_SA_-N-HCS-900, and Co_2_-N-HCS-900; **d**, **e** k^3^-Weighted EXAFS fitting curves at R space for Co_SA_-N-HCS-900 and Co_2_-N-HCS-900 (inset: schematic structure of CoN_4_ and Co_2_N_5_ models; green: Co, blue: N, red: O); **f** X-band electron paramagnetic resonance (EPR) spectra and **g** magnetic hysteresis loops at room temperature (300 K) and inset image of the magnification of magnetic hysteresis loops around H = 0; **h** M-T curves and **i** corresponding unpaired electron *n* and effective magnetic moment (*μ*_*eff*_) for Co_SA_-N-HCS-900 and Co_2_-N-HCS-900. **j** Work function spectra for Co_NP_/HCS-900, Co_SA_-N-HCS-900, and Co_2_-N-HCS-900.

X-ray absorption spectroscopy (XAS) was employed to elucidate the detailed local structures of the Co atoms in Co_SA_-N-HCS-900 and Co_2_-N-HCS-900. As can be seen from the X-ray absorption near edge structure (XANES) spectra in Fig. 4a, the Co K-edge XANES curves for Co_2_-N-HCS-900 and Co_SA_-N-HCS-900 lie between those for Co foil and Co_3_O_4_, indicating that their Co atoms show a positive valence. Figure S37 displays the Fourier transform (FT) k^3^-weighted extended X-ray absorption fine structure (FT-EXAFS) spectra at R spaces, in which the main peak at 1.43 Å that corresponds to the Co-N shell (relative to CoPc) is obtained for both Co_SA_-N-HCS-900 and Co_2_-N-HCS-900. The secondary peak appearing at R = 2.25 Å was observed for Co_2_-N-HCS-900, which can be indexed to the Co-Co shell in reference to Co foil, indicating the formation of Co-Co bonds in the catalysts. In addition, a comparison of the q-space magnitudes in Fig. 4b confirms the existence of Co-N and Co-Co paths in Co_2_-N-HCS-900, whereas only the Co-N path exists in Co_SA_-N-HCS-900. Further support for the Co-Co path in Co_2_-N-HCS-900 is provided by wavelet transform (WT) contour plots (Fig. 4c). To determine the detailed coordination structure of the Co atoms in both Co_SA_-N-HCS-900 and Co_2_-N-HCS-900, theoretical calculations and fitted EXAFS at the Co K-edge were conducted. As shown in Fig. 4d and Table S1, Co_SA_-N-HCS-900 provides an average Co-N/O coordination number of 4.0 and a Co-Co coordination number of 0.1, indicating that Co atoms predominantly exist in the form of a CoN_4_ structure (inset of Fig. 4d). As for Co_2_-N-HCS-900, the average coordinated number of Co-N/O bond and Co-Co bond were determined to be 3.9 and 0.8 (close to 1), respectively, meaning that most Co atoms are preferentially bonded with one Co and three N atoms (and one O) to form Co_2_N_5_-O structure (inset of Fig. 4e). To further verify the possible structures of Co_SA_-N-HCS-900 and Co_2_-N-HCS-900, DFT calculations were performed to investigate the possible structures containing a single Co atom and a paired Co_2_ structure (models 1 to 10, Fig. S38). Moreover, a comparison between the simulated EXAFS and XANES spectra of the possible structures and the experimental spectra was recorded. As shown in Figure S39 and S40 and Table S2, the simulated spectra based on the single atom CoN_4_ model (model 9) agreed well with the experimental EXAFS and XANES results of Co_SA_-N-HCS-900, confirming that this model is the most likely actual structure of Co_SA_-N-HCS-900. For Co_2_-N-HCS-900, the simulated spectra based on model 2 (binuclear Co_2_N_5_ configurations with oxygen) agreed well with the experimental EXAFS and XANES results (Figure S41 and S42 and Table S3), indicating that model 2 is the most likely actual structure. These results synergistically validated that CoN_4_ single atoms could couple with each other to form paired Co_2_N_5_ dual atom dimers via thermal migration.

X-ray photoelectron spectroscopy (XPS) was conducted to explore the chemical elements and states of Co_NP_/HCS-900, Co_SA_-N-HCS-900, and Co_2_-N-HCS-900 (Figure S43a). The high-resolution C 1 *s* XPS spectra of the three investigated catalysts (Figure S44) can be divided into four peaks at approximately 284.7, 285.2, 286.3, and 290.3 eV, which are indexed to *sp*^*2*^ C = C and *sp*^*3*^ C-C (defects), C-N, and COOH species, respectively. The high-resolution N 1 *s* XPS spectra in Figure S45 confirm the coexistence of pyridinic N, Co-Nx, graphitic N, and oxidized N^50^. Figure S46 shows that Co_SA_-N-HCS-900 affords a higher content of C-N, pyridinic N, and Co-Nx species than Co_NP_/HCS-900, indicating that melamine (N agent) could be coordinated with Co atoms to form Co-Nx moieties to promote Co nanoparticle atomization. Further, both the content of pyridinic N and C-N species show a downward trend with calcination time, revealing that the C-N bonds coordinated with pyridinic N are preferentially cleaved^51,52^. This would lead to a decrease in total N content (Figure S43b) and generate many defective structures, which is further evidenced by the high *I*_D_/*I*_G_ ratio (1.48, Figure S47) and large Brunauer–Emmett–Teller (BET) surface area (772.9 m^2^ g^−1^, Figure S48a) and pore volumes (1.13 cm^3^ g^−1^, Figure S48b) for Co_2_-N-HCS-900^53–55^. The Co 2*p* XPS spectra in Figure S49 shows that four peaks attributed Co-N species and corresponding satellite peaks are obtained for the investigated catalysts, while two peaks indexing Co^0^ species are observed in the Co_NP_/HCS-900, indicating the existence of metallic Co nanoparticles in Co_NP_/HCS-900. As displayed in Figure S50, the deconvolution of the O 1 *s* spectra demonstrated the coexistence of oxygen-containing functional groups (C = O at ca. 531.8 eV, COOH at ca. 533.3 eV, and absorbed water at ca. 536.3 eV) in Co_NP_/HCS-900, Co_SA_-N-HCS-900, and Co_2_-N-HCS-900. Interestingly, a new peak appears at 530.2 eV that was indexed to the Co-O bond for Co_2_-N-HCS-900, indicating the presence of Co-O bonds in the catalyst. The Co content, measured by inductively coupled plasma optical emission spectrometry (ICP-OES), was determined to be 1.48 wt% for Co_NP_/HCS-900, 1.41 wt% for the Co_SA_-N-HCS-900, and 1.74 wt% for the Co_2_-N-HCS-900. Considering the BET surface area and Co content, the Co_2_-N-HCS-900 affords a higher atomic Co coverage of 0.233 atoms per square nanometer than the Co_SA_-N-HCS-900 (0.196 atoms per square nanometer), demonstrating more accessible active Co sites on the former.

X-band electron paramagnetic resonance (EPR) measurement is a powerful tool to investigate the paramagnetic properties of catalysts. As shown in Fig. 4f, a strong signal was detected for Co_NP_/HCS-900, indicating that it is paramagnetic. A downward trend was observed for Co_NP_/HCS-900 to Co_SA_-N-HCS-900 to Co_2_-N-HCS-900, indicating that the spin magnetic moment decreased from the Co nanoparticles to single CoN_4_ sites and then to paired Co_2_N_5_ sites. Notably, no signal appeared for Co_2_-N-HCS-900, possibly because of the formation of a binuclear Co structure with antiferromagnetic coupling sites, again confirming that the paired Co_2_N_5_ structure was successfully constructed in Co_2_-N-HCS-900. As shown in Fig. 4g, the ferromagnetic hysteresis loops of the investigated catalysts at 300 K exhibited saturation magnetization. Notably, the saturation magnetization decreased from Co_NP_/HCS-900 to Co_SA_-N-HCS-900 to Co_2_-N-HCS-900. An enlarged view of the curve around H = 0 indicates that the Co_2_-N-HCS-900 provides the lowest coercive magnetic field and residual magnetization (Fig. 4g). To further reveal the electron-spin configurations of the investigated catalysts, zero-field cooling (ZFC) temperature-dependent magnetic susceptibility measurements were conducted (Fig. 4h and S51). As presented in Fig. 4h and i, the effective magnetic moment of Co_SA_-N-HCS-900 and Co_2_-N-HCS-900 were calculated to be 2.6 and 1.7 *μ*_*eff*_, respectively. The average number of unpaired electrons is 1.0 in the Co-3*d* orbitals for Co_2_-N-HCS-900, which is lower than that of Co_SA_-N-HCS-900 (1.7), indicating decreased electron spin polarization from Co_SA_-N-HCS-900 to Co_2_-N-HCS-900. Based on these results, it can be concluded that the spin magnetic moment exhibits a downward trend from Co_NP_/HCS-900 to Co_SA_-N-HCS-900 to Co_2_-N-HCS-900. Ultraviolet photoemission spectroscopy (UPS) was used to investigate the electronic states of Co_NP_/HCS-900, Co_SA_-N-HCS-900, and Co_2_-N-HCS-900. The work function represents the minimum energy required for the inner electrons to escape from the catalyst surface. As shown in Fig. 4j and S52, the work function of Co_2_-N-HCS-900 was determined to be 5.04 eV, which is higher than those of Co_SA_-N-HCS-900 (4.90 eV) and Co_NP_/HCS-900 (4.52 eV). The valence band maximum is referred to as the highest occupied molecular orbital (HOMO) and is related to the highest energy level of the valence band in the solid material. It is widely recognized that shifts in the valance band indicate changes in the E_*d*_ energy level, primarily because valence electrons near the Fermi level significantly influence the *d* states^56^. The Co_2_-N-HCS-900 exhibits a high calculated valance band maximum (VBM) value of 5.63 eV compared to that of Co_SA_-N-HCS-900 (5.00 eV) and Co_NP_/HCS-900 (4.41 eV) (Fig. 4j, S52, and S53), indicating that the valance band gets away from the Fermi level for the Co_2_-N-HCS-900. The larger work function and VBM indicate that Co_2_-N-HCS-900 presents a higher energy barrier for electron donation and possesses a reduced E_*d*_ energy level, resulting in favorable interactions between the intermediates and active sites and enhanced reaction activity^56^.

### Evaluation of electrochemical performance

**Fig. 5:**
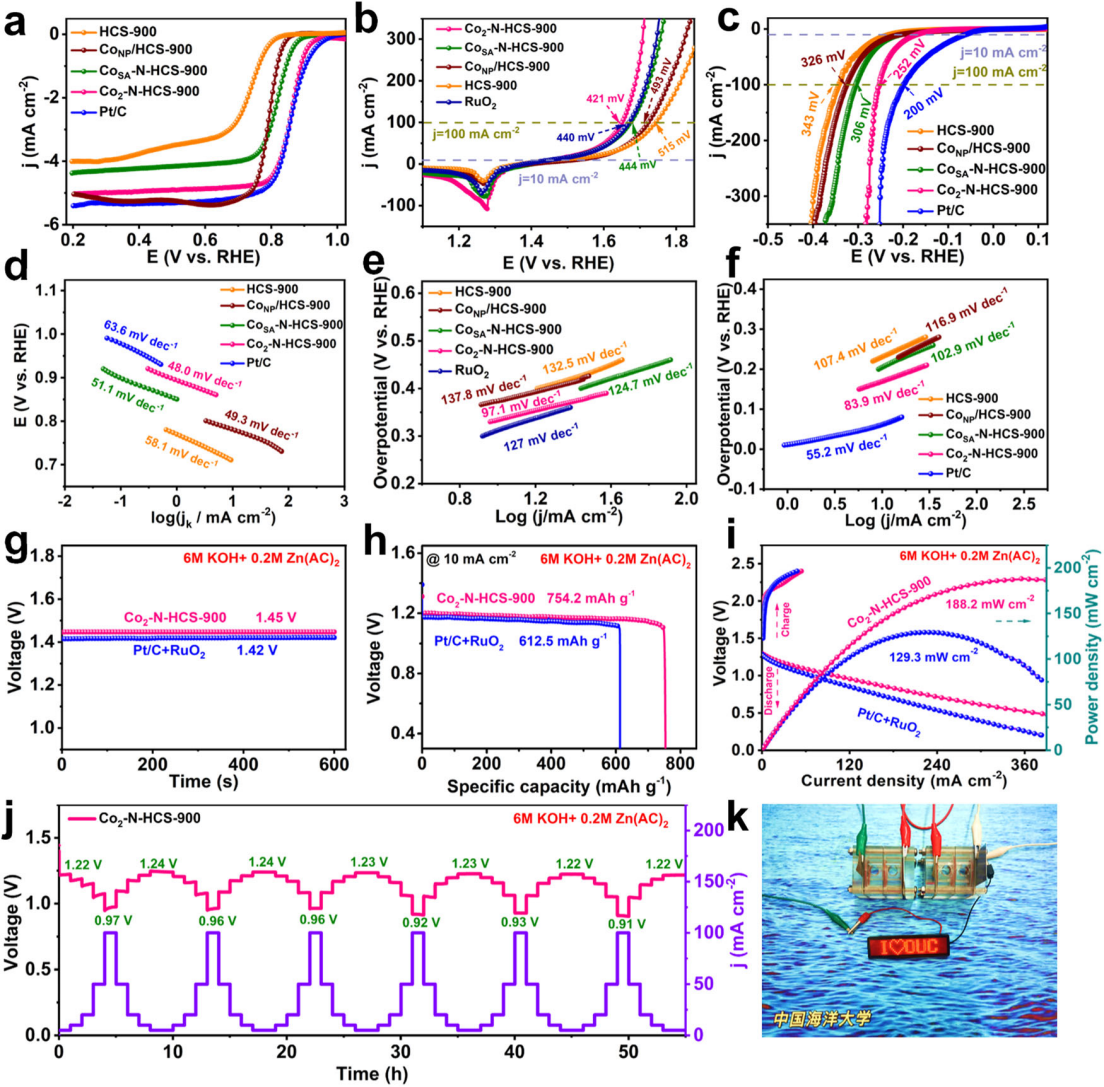
Electrocatalytic activity and the performance of ZABs. **a** LSV curves and **d** Tafel plots toward ORR in 0.1 M KOH; **b** LSV curves and **e** Tafel plots toward OER; and **c** LSV curves and **f** Tafel plots toward HER in 1.0 M KOH for all investigated and commercial (Pt/C or RuO_2_) catalysts. **g** Open-circuit potential plots; **h** specific capacities; and **i** discharge polarization, charge polarization, and corresponding power density curves for ZABs driven by Co_2_-N-HCS-900 or commercial Pt/C + RuO_2_. **j** Discharge curves of ZABs at various discharge current densities, and **k** LED screen powered by two tandem Co_2_-N-HCS-900-based ZABs.

The ORR/OER/HER performances of the obtained catalysts were then investigated (Fig. 5a–f). Linear sweep voltammetry (LSV) curves show that Co_2_-N-HCS-900 exhibits a more positive onset potential (0.99 V) and half-wave potential (0.86 V) toward ORR than HCS-900, Co_NP_/HCS-900, and Co_SA_-N-HCS-900 (Fig. 5a and S54), which are comparable to those of commercial Pt/C and other reported non-precious M-N-C catalysts (Table S4). The superior ORR kinetics of Co_2_-N-HCS-900 are evident as it demonstrates the smallest Tafel slope (48.0 mV dec^−1^) and highest kinetic current density (8.33 mA cm^−2^ @ 0.85 V), as shown in Fig. 5d and S55. Moreover, Co_2_-N-HCS-900 exhibits a higher electrochemical double-layer capacitance (C_dl_) value of 195.1 mF cm^−2^ and mass activity of 97.6 A g^−1^_Co_ @ 0.9 V than the control catalysts (Co_NP_/HCS-900, Co_SA_-N-HCS-900, and commercial Pt/C). This suggests that more abundant and accessible active sites for ORR catalysis are present (Figure S56–58). Koutecky–Levich (K–L) plots derived from the LSV curves indicate that the ORR kinetics of Co_2_-N-HCS-900 are closely related to the O_2_-diffusion process (Figure S59). The electron transfer number (*n*) is determined to be 3.66, indicating a direct 4*e*^-^ reduction pathway during ORR (Figure S59). Additionally, Co_2_-N-HCS-900 exhibits the lowest OER overpotentials of 333 mV at 10 mA cm^−2^ and smallest Tafel slope (97.1 mV dec^−1^) of the investigated catalysts, rivaling that of commercial RuO_2_ and other advanced M-N-C catalysts (Figs. 5b, e, and Table S5). For the HER, Co_2_-N-HCS-900 achieved good activity in view of its low overpotential (166 mV at 10 mA cm^−2^ and 252 mV at 100 mA cm^−2^) and small Tafel slopes (83.9 mV dec^−1^), surpassing those of HCS-900, Co_NP_/HCS-900, and Co_SA_-N-HCS-900 and comparable to those of most reported M-N-C catalysts (Table S6). Further, Co_2_-N-HCS-900 exhibits a large mass activity of 2.79 A g^−1^_Co_ @ η = 400 mV toward OER and 1.31 A g^−1^_Co_ @ η = 200 mV toward HER, exceeding those of the control Co_NP_/HCS-900, Co_SA_-N-HCS-900, and commercial Pt/C (Figure S60 and S61). These results highlight the significant role of the adjacent Co atoms in the Co_2_N_5_ structure in realizing advanced trifunctional activities, which also have advantages over currently reported multifunctional single atom catalysts (Table S7). Moreover, Co_2_-N-HCS-900 confers superior long-term ORR/OER/HER stability compared to commercial catalysts (Pt/C or RuO_2_, Figure S62–64).

The ORR/OER activity of Co_2_-N-HCS-900 motivated us to investigate its practical application in assembled liquid ZABs. Figure 5g–i and Table S8 show that the ZABs driven by Co_2_-N-HCS-900 demonstrate a high open-circuit potential (OCP) of 1.45 V, a large specific capacity of 754.2 mAh g^−1^, and an eminent peak power density of 188.2 mW cm^−2^, far surpassing commercial Pt/C + RuO_2_ based ZABs (1.42 V, 612.5 mAh g^−1^, and 129.3 mW cm^−2^). The discharge curves in Fig. 5j show that Co_2_-N-HCS-900-based ZABs provide a voltage of 1.22 V (5 mA cm^−2^) and 0.97 V (100 mA cm^−2^) during the first cycle, and the voltage loss is negligible after 6 cycles, validating its excellent rate performance and reversibility. The ZABs powered by Co_2_-N-HCS-900 also possessed an excellent round-trip efficiency of 58.1% at a current density of 5 mA cm^−2^ and an ultralong lifespan of over 800 h with negligible round-trip efficiency fading (Figure S65 and S66). Notably, the Co_2_-N-HCS-900-based ZABs can operate over 600 cycles under a high current density of 50 mA cm^−2^ (Figure S67), demonstrating its promising practical application. Moreover, two or three tandem Co_2_-N-HCS-900-based ZABs offer an OCP of 2.91 or 4.35 V, respectively, which could power a light-emitting diode screen (LED, 2 V) for several hours (Fig. 5k and S68).

**Fig. 6:**
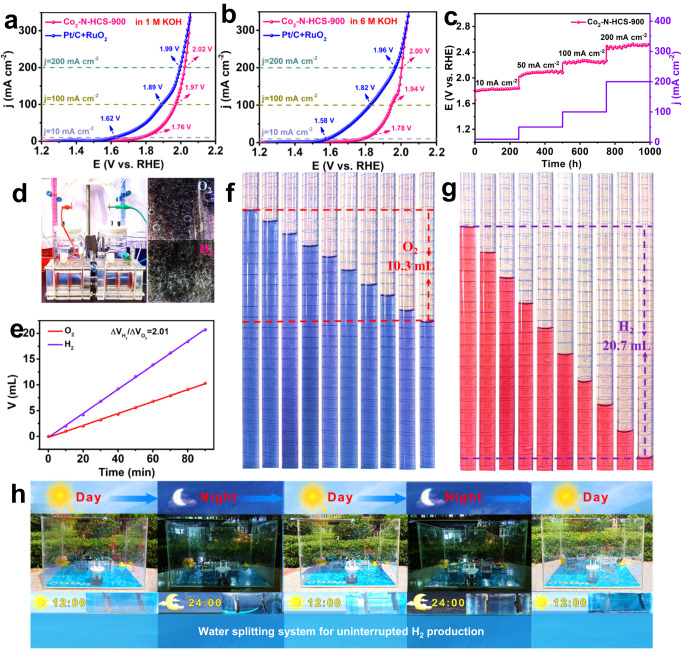
Electrocatalytic overall water-splitting performance. **a**, **b** LSV curves in 1.0 M KOH and 6.0 M KOH. **c** Stability test for overall water splitting cells. **d** Digital image of the water splitting powered by ZABs. **e** Volumes of O_2_ and H_2_ evolved with time. **f**, **g** Enlarged digital images of the measured gas quantities generated at 10 min intervals for 90 min. **h** Photograph of series WSS driven by Co_2_-N-HCS-900 during day and night over 48 h.

Leveraging its good bifunctional OER/HER activity, water electrolysis devices were assembled using Co_2_-N-HCS-900 as the anode and cathode catalysts. The Co_2_-N-HCS-900-based system requires potentials of approximately 1.76, 1.97, and 2.02 V to achieve current densities of 10, 100, and 200 mA cm^−2^, respectively, which can compete with those of commercial Pt/C + RuO_2_ counterparts (~1.62, 1.89, and 1.99 V, Fig. 6a). Figure 6b displays the overall water-splitting performance of the Co_2_-N-HCS-900-based devices in a 6 M KOH solution. A large potential is required to achieve a current density of 10 mA cm^−2^ in comparison with its commercial Pt/C + RuO_2_ counterparts, but the potential gaps gradually become narrower as the current densities increase from 10 to 200 mA cm^−2^, again emphasizing its good overall water splitting activity. In addition, the Co_2_-N-HCS-900-based splitting devices maintained a constant potential along with negligible changes after continuous operation for 1000 h in 1 M KOH (Fig. 6c), underlining the excellent stability of Co_2_-N-HCS-900. Inspired by its excellent trifunctional ORR/OER/HER performance, a cell including two tandem ZABs and water electrolysis devices was established using only Co_2_-N-HCS-900 (Fig. 6d). The cell could provide an ultra-high H_2_ production rate of 616 µmol h^−1^, which was determined by the volume of generated H_2_ (20.7 mL) and O_2_ (10.3 mL) over a total of 90 min (Fig. 6e–g). The quantified generated H_2_/O_2_ ratio was calculated to be approximately 2.01, which agrees well with the theoretical ratio (Fig. 6e). As the proof-of-concept, a highly efficient and durable solar-powered WSS that integrates polycrystalline Si solar panels, three tandem ZABs (with an OCP of 4.35 V, Figure S69), and water electrolysis devices was also constructed. The WSS could ensure uninterrupted H_2_ production for 48 h, throughout day and night (Fig. 6h and S69), showcasing the promising potential of Co_2_-N-HCS-900 for uninterrupted, large-scale H_2_ production.

In summary, a NP-to-SA-to-DA atomization and sintering strategy was implemented to achieve a controllable adjustment of the existing configuration states from Co nanoparticles to CoN_4_ single atoms to Co_2_N_5_ dual atoms at the atomic level. We discovered that dual atom Co_2_N_5_ with low spin magnetic moments disrupts the conventional ΔG_OOH*_ to ΔG_OH*_ scaling relations, achieving optimal O* adsorption and moderated H* adsorption/desorption. As expected, Co_2_-N-HCS-900 exhibited enhanced multifunctional ORR/OER/HER activity, which enabled the solar-powered WSS to produce H_2_ continuously over 48 h. More importantly, this universal strategy can be broadened to transform 22 types of conventional *s*-, *p*-, and *d*-block metals or their alloys into dual atom structures. This work both provides a systematic investigation of the formation mechanisms of dual atom catalysts and emphasizes a universal strategy to synthesize dual atom catalysts in pursuit of a breakthrough in multifunctional activities, motivating the rational design of highly efficient multifunctional dual atom catalysts for application in renewable energy conversion technologies.

## Methods

### Chemicals

Hexamethylenetetramine (HMT), 2,4-dihydroxybenzoic acid (DA), sodium oleate (SO), cobalt chloride hexahydrate (CoCl_2_ · 6H_2_O), nickel chloride hexahydrate (NiCl_2_ · 6H_2_O), iron chloride hexahydrate (FeCl_3_ · 6H_2_O), manganese acetate (Mn(CH_3_COO)_2_ · 4H_2_O), copper chloride dihydrate (CuCl_2_ · 2H_2_O), zinc acetate dihydrate (Zn(CH_3_COO)_2_ · 2H_2_O), antimony trichloride (SbCl_3_), calcium acetate monohydrate (Ca(CH_3_COO)_2_ · H_2_O), bismuth nitrate pentahydrate (Bi(NO_3_)_3_ · 5H_2_O), chromium chloride hexahydrate (CrCl_3_ · 6H_2_O), cerium nitrate hexahydrate (Ce(NO_3_)_3_ · 6H_2_O), ruthenium(III) chloride anhydrous (RuCl_3_), aluminum chloride hexahydrate (AlCl_3_ · 6H_2_O), melamine, and n-pentane were obtained from Aladdin Chemical Co. Pluronic P123 (nonionic triblock copolymer, EO_20_PO_70_EO_20_) and RuO_2_ were purchased from Sigma-Aldrich. A commercial Pt/C catalyst (20 wt%) was acquired from Johnson Matthey Co. (Shanghai, China).

### Preparation of hollow polymer spheres (HPS)

The HPS precursor was prepared using the following method^57,58^. An aqueous solution A containing 0.375 mM Pluronic P123 and 12 mM SO was injected into aqueous solution B containing 8.3 mM HMT and 20 mM DA. The mixed solution was then transferred to a Teflon-lined stainless-steel autoclave and heated to 160 °C for 2 h. Finally, the HPS precursors were obtained by centrifuging with water and dried at 50 °C for 24 h.

### Synthesis of Co_2_N_5_ dual atom on the N-doped hollow carbon spheres (Co_2_-N-HCS-900)

HPS (50 mg) was dispersed in n-pentane (10 mL) by ultrasonication for 30 min and stirred for 30 min at room temperature. A certain amount of CoCl_2_^.^6H_2_O solution was injected into the HPS solution and then continuously stirred for 12 h at room temperature to evaporate the solvent. The precursor (denoted as Co-HPS) was obtained after drying in a vacuum at 50 °C for 24 h. Finally, the Co-HPS precursor and melamine with the ratio of 1:10 were ground together and placed into a tube furnace, heated to 900 °C, and maintained there for 180 min under N_2_ atmosphere to yield the final catalysts with 20% yield of Co-HPS (termed Co_2_-N-HCS-900).

### Synthesis of CoN_4_ single atom on the N-doped hollow carbon spheres (Co_SA_-N-HCS-900)

Co-HPS precursor and melamine with the ratio of 1:10 were ground together and then placed into a tube furnace, heated to 900 °C, and maintained there for 30 min under N_2_ atmosphere to yield the Co_SA_-N-HCS-900 catalysts (around 20% yield of Co-HPS).

### Synthesis of Co nanoparticles on the hollow carbon spheres (Co_NP_/HCS-900)

The Co-HPS precursor was directly placed into a tube furnace, heated to 900 °C, and maintained there for 180 min under N_2_ atmosphere to yield Co_NP_/HCS-900 (around 20% yield of Co-HPS).

### Synthesis of hollow carbon spheres (HCS-900)

The HPS precursor was directly placed into a tube furnace, heated to 900 °C, and maintained there for 180 min under N_2_ atmosphere to yield HCS-900 (around 20% yield of HPS).

### Preparation of 21 different dual atom catalysts

The Al_2_-N-HCS-900, Ca_2_-N-HCS-900, Cr_2_-N-HCS-900, Mn_2_-N-HCS-900, Fe_2_-N-HCS-900, Ni_2_-N-HCS-900, Cu_2_-N-HCS-900, Zn_2_-N-HCS-900, Ru_2_-N-HCS-900, Sb_2_-N-HCS-900, Ce_2_-N-HCS-900, Bi_2_-N-HCS-900, CoFe-N-HCS-900, CoNi-N-HCS-900, CoCu-N-HCS-900, CoZn-N-HCS-900, CoMn-N-HCS-900, FeNi-N-HCS-900, FeCu-N-HCS-900, FeZn-N-HCS-900, and FeMn-N-HCS-900 were synthesized using a similar procedure to that used for fabricating Co_2_-N-HCS-900, except the metal Co salts solution were replaced with a AlCl_3_ · 6H_2_O, Ca(CH_3_COO)_2_ · H_2_O, CrCl_3_ · 6H_2_O, Mn(CH_3_COO)_2_ · 4H_2_O, FeCl_3_ · 6H_2_O, NiCl_2_ · 6H_2_O, CuCl_2_ · 2H_2_O, Zn(CH_3_COO)_2_ · 2H_2_O, RuCl_3_, SbCl_3_, Ce(NO_3_)_3_ · 6H_2_O, or Bi(NO_3_)_3_ · 5H_2_O solution or a two-mixture solution (1:1).

### Characterization

Transmission electron microscopy (TEM) was performed using a Hitachi H-7650 microscope. High-resolution TEM (HRTEM), high-angle annular dark-field scanning transmission electron microscopy (HAADF-STEM), and energy-dispersive X-ray spectroscopy (EDS) were performed using a JEM-2100F microscope. Aberration-corrected (AC) HAADF-STEM was employed on a Titan-Cubed Themis G2. X-ray absorption near edge structure (XANES) and extended X-ray absorption fine structure (EXAFS) measurements of the Co K-edge were performed in the fluorescence mode at beamline BL14W1. The X-ray diffraction (XRD) patterns were recorded using a Bruker D8 Advance diffractometer. Raman spectra were obtained using a Thermo Fisher spectrometer equipped with helium-neon (633 nm) and argon (532 nm) lasers. X-ray photoelectron spectroscopy (XPS) was conducted using a Thermo ESCALAB 250XI instrument. Ultraviolet photoelectron spectroscopy (UPS) was performed using a Thermo ESCALAB Xi+ instrument equipped with an ultraviolet photoelectron spectroscope (Hel (21.22 eV)). Inductively coupled plasma-optical emission spectrometry (ICP-OES) was used to precisely detect the Co content. Brunauer–Emmett–Teller (BET) analysis was used to investigate the specific surface areas of the catalysts.

### Electrochemical measurements

The ORR, OER, and HER performances were investigated using an electrochemical station (CHI-660E) equipped with a conventional three-electrode system. For the OER and HER, the catalyst-modified pretreated Ni foam, graphite rods, and Hg/HgO electrodes were used as the working, counter, and reference electrodes, respectively. A total of 10 mg catalysts were dispersed into 1 mL Nafion-solution containing water, isopropanol, and Nafion (v/v/v = 4:1:0.1), and then ultrasonicated for 1 h. Then, 100 μL of the suspension was pipetted onto pre-treated Ni foam (1 cm^2^) and dried under an infrared lamp; this was used as the working electrode with a loading amount of 1 mg cm^−2^. Linear sweep voltammetry (LSV) was used to evaluate the OER and HER performances of the catalysts in 1 M KOH with iR compensation. Long-term stability tests were conducted using chronopotentiometric measurements. The overall water-splitting performance of the two-electrode electrolysis devices was investigated in 1.0 and 6.0 M KOH solutions.

For the ORR, a catalyst-modified glassy carbon electrode (GCE), a Pt wire, and a Ag/AgCl (KCl-saturated) electrode were used as the working, counter, and reference electrodes, respectively. 2 mg catalysts were added to 1 mL Nafion-solution and ultrasonicated for 1 h. Then, 27 μL of the suspension was pipetted onto a polished rotating disk electrode (RDE, diameter: 4 mm) or rotating ring disk electrode (RRDE, diameter: 4 mm), which was used as the working electrode with a loading amount of 0.43 mg cm^−2^. Linear sweep voltammetry (LSV) was used to explore the ORR performance in O_2_-saturated 0.1 M KOH at a rotation rate of 1600 rpm.

### Aqueous Zn-air battery assembly

Homemade aqueous zinc-air batteries (ZABs) were established to assess their practical applications. Polished zinc foil was used as the anode, and a hydrophilic carbon fiber paper substrate coated with a catalyst layer (1 mg cm^−2^) was used as the air cathode. A solution of 6.0 M KOH + 0.2 M Zn(CH_3_COO)_2_ was used as the electrolyte in the ZABs. LSV measurements were performed on a CHI-660 electrochemical workstation at a scan rate of 10 mV s^–1^ at room temperature. The galvanostatic charge and discharge measurements were performed at room temperature by a LAND testing system at 5 mA cm^−2^ with 5 min of discharge followed by 5 min of charge.

### Computational methods

All calculations were performed within the framework of the density functional theory (DFT) as implemented in the Vienna Ab initio simulation package (VASP 5.4.4) code within the Perdew–Burke–Ernzerhof (PBE) generalized gradient approximation and projected augmented wave (PAW) method^34,59–61^. The cut-off energy for the plane-wave basis set was set to 400 eV. An ultrasoft pseudopotential was employed to describe the interaction between the valence electrons and ionic core. The Brillouin zone was sampled using gamma-centered 1 × 1 × 1 k-point meshes to perform geometry optimization and electronic structure calculations. During the geometry optimization, all atoms were allowed to relax without any constraints until the convergence thresholds of maximum force and energy were smaller than 0.05 eV/Å and 1.0 × 10^−4^ eV/atom, respectively. A vacuum layer of 30 Å was introduced to avoid interactions between periodic images. In addition, van der Waals (vdW) interactions were described by Grimme’s DFT-D3 scheme with the application of dispersion correction^62^. Furthermore, transition states were searched using the climbing image nudged-elastic-band (CI-NEB) method combined with the VTST code^37,63^.

### Supplementary information

Supplementary Information

Peer Review File

### Source data

Source Data

## Data Availability

All the data supporting this study are available in the paper and Supplementary Information. Additional data related to this study are available from the corresponding authors on reasonable request. Source data are provided with this paper.
