# Supplementary material for: Developing a class of dual atom materials for multifunctional catalytic reactions
Source: Nat Commun. 2023 Nov 8;14:7210. doi: 10.1038/s41467-023-42756-8 (PMC10632389; doi:10.1038/s41467-023-42756-8)
Supplement: Supplementary file 1 — Supplementary Information [file 41467_2023_42756_MOESM1_ESM.pdf]

Supporting Information

Developing a class of dual atom materials for multifunctional catalytic reactions

Xingkun Wang[1,2,3,4#], Liangliang Xu[5#], Cheng Li[6,7,8#], Canhui Zhang[1#], Hanxu Yao[2,3,4], Ren Xu[1], Peixin Cui[9], Xusheng Zheng[10], Meng Gu[6,7], Jinwoo Lee[5*], Heqing Jiang[2,3,4]*, Minghua Huang[1]*

[1] *School of Materials Science and Engineering, Ocean University of China, Qingdao, 266100, China.*
[2] *Qingdao Key Laboratory of Functional Membrane Material and Membrane Technology, Qingdao Institute of Bioenergy and Bioprocess Technology, Chinese Academy of Sciences, Qingdao 266101, China.*
[3] *Shandong Energy Institute, Qingdao 266101, China.*
[4] *Qingdao New Energy Shandong Laboratory, Qingdao 266101, China.*
[5] *Department of Chemical and Biomolecular Engineering, Korea Advanced Institute of Science and Technology (KAIST), 291 Daehak-Ro, Yuseong-Gu, Daejeon 34141, Republic of Korea*
[6] *Eastern Institute for Advanced Study, Eastern Institute of Technology, Ningbo, Zhejiang 315200, P. R. China*
[7] *Department of Materials Science and Engineering, Southern University of Science and Technology, Shenzhen, 518055, China.*
[8] *School of Physics and Astronomy, University of Birmingham, Birmingham, B15 2TT, UK*
[9] *Key Laboratory of Soil Environment and Pollution Remediation, Institute of Soil Science, Chinese Academy of Sciences, Nanjing, 210008 China*
[10] *National Synchrotron Radiation Laboratory (NSRL), University of Science and Technology of China, Hefei, 230029 China*

Corresponding Author: huangminghua@ouc.edu.cn, jianghq@qibebt.ac.cn, jwlee1@kaist.ac.kr

Contents

Supplemental Figures and Tables 6

Figure S1. Calculated relative energies along the stretching pathway of the Co atom from Co_{16} to Co_{SA}/C or CoN_4 model by CI-NEB. 6

Figure S2. Calculated relative energies along the stretching pathway of the Co atom from Co_4 to Co_{SA}/C or CoN_4 model by CI-NEB. 7

Figure S3. (a-f) Calculated relative energies of different CoN_4 models that are separated by several carbon atoms, Co_2N_6, and Co_2N_5 models. 8

Figure S4. (a) Calculated relative energies along the stretching pathway of the Al atom from Al_{10} to Al_{SA}/C or AlN_4 model by CI-NEB. (b) Calculated relative energies of AlN_4, Al_2N_6, and Al_2N_5 models. 9

Figure S5. (a) Calculated relative energies along the stretching pathway of the Ca atom from Ca_{10} to Ca_{SA}/C or CaN_4 model by CI-NEB. (b) Calculated relative energies of CaN_4, Ca_2N_6, and Ca_2N_5 models. 10

Figure S6. (a) Calculated relative energies along the stretching pathway of the Cr atom from Cr_{10} to Cr_{SA}/C or CrN_4 model by CI-NEB. (b) Calculated relative energies of CrN_4, Cr_2N_6, and Cr_2N_5 models. 11

Figure S7. (a) Calculated relative energies along the stretching pathway of the Mn atom from Mn_{10} to Mn_{SA}/C or MnN_4 model by CI-NEB. (b) Calculated relative energies of MnN_4, Mn_2N_6, and Mn_2N_5 models. 12

Figure S8. (a) Calculated relative energies along the stretching pathway of the Fe atom from Fe_{10} to Fe_{SA}/C or FeN_4 model by CI-NEB. (b) Calculated relative energies of FeN_4, Fe_2N_6, and Fe_2N_5 models. 13

Figure S9. (a) Calculated relative energies along the stretching pathway of the Ni atom from Ni_{10} to Ni_{SA}/C or NiN_4 model by CI-NEB. (b) Calculated relative energies of NiN_4, Ni_2N_6, and Ni_2N_5 models. 14

Figure S10. (a) Calculated relative energies along the stretching pathway of the Cu atom from Cu_{10} to Cu_{SA}/C or CuN_4 model by CI-NEB. (b) Calculated relative energies of CuN_4, Cu_2N_6, and Cu_2N_5 models. 15

Figure S11. (a) Calculated relative energies along the stretching pathway of the Zn atom from Zn_{10} to Zn_{SA}/C or ZnN_4 model by CI-NEB. (b) Calculated relative energies of ZnN_4, Zn_2N_6, and Zn_2N_5 models. 16

Figure S12. (a) Calculated relative energies along the stretching pathway of the Ru atom from Ru_{10} to Ru_{SA}/C or RuN_4 model by CI-NEB. (b) Calculated relative energies of RuN_4, Ru_2N_6, and Ru_2N_5 models. 17

Figure S13. (a) Calculated relative energies along the stretching pathway of the Sb atom from Sb_{10} to Sb_{SA}/C or SbN_4 model by CI-NEB. (b) Calculated relative energies of SbN_4, Sb_2N_6, and Sb_2N_5 models. 18

Figure S14. (a) Calculated relative energies along the stretching pathway of the Bi atom from Bi_{10} to Bi_{SA}/C or BiN_4 model by CI-NEB. (b) Calculated relative energies of BiN_4, Bi_2N_6, and Bi_2N_5 models. 19

Figure S15. (a) Calculated relative energies along the stretching pathway of the Ce atom from Ce_{10} to Ce_{SA}/C or CeN_4 model by CI-NEB. (b) Calculated relative energies of CeN_4, Ce_2N_6, and Ce_2N_5 models. 20

Figure S16. (a) Calculated relative energies along the stretching pathway of the Fe/Ni atom from Fe_5Ni_5 to Fe_{SA}/Ni_{SA}-C or FeN_4/NiN_4 model by CI-NEB. (b) Calculated relative energies of FeN_4/NiN_4, $FeNiN_6$, and $FeNiN_5$ models. 21

Figure S17. Optimized atomic configurations of (a) Co_{16}, (b) Co_{10}, (c) Co_4, (d) CoN_4 and (e) Co_2N_5 model. 22

Figure S18. PDOS of the (a) Co_{16}, (b) Co_{10}, (c) Co_4, (d) CoN_4, and (e) Co_2N_5 models. 23

Figure S19. Calculated adsorption free energies of O* intermediates on the Co_{10} nanoparticles, CoN_4 single atom, and Co_2N_5 dual atom models, respectively. 24

Figure S20. Crystal Orbital Hamilton Population (COHP) analysis corresponding integrated-COHP (-ICOHP) value of the Co_{10}-O (a), CoN_4-O (b) and Co_2N_5-O (c). 25

Figure S21. The COHP analysis (a) and the magnification around Fermi level image (b) of the Co_{10}-O, CoN_4-O and Co_2N_5-O. 26

Figure S22. Calculated adsorption free energies of H* intermediates on the Co_{10} nanoparticles, CoN_4 single atom and Co_2N_5 dual atom models, respectively. 27

Figure S23. Optimized models and atomic configurations of oxygen-containing species (OOH*, O* and OH*) adsorbed on (a) Co_{16}, (b) Co_{10}, (c) Co_4, (d) CoN_4, and (e) Co_2N_5 models. 28

Figure S24. Optimized models and atomic configurations of H* species toward HER adsorbed on (a) Co_{16}, (b) Co_{10}, (c) Co_4, (d) CoN_4, and (e) Co_2N_5 models. 29

Figure S25. Free energy diagram of (a) Co_{16}, (b) Co_{10} and (c) Co_4 models for ORR. 30

Figure S26. Free energy diagram of (a) Co_{16}, (b) Co_{10} and (c) Co_4 models for OER. 31

Figure S27. Free energy diagram of (a) Co_{16}, (b) Co_{10} and (c) Co_4 models for HER. 32

Figure S28. XRD patterns for HCS-900, Co_{NP}/HCS-900, Co_{SA}-N-HCS-900, and Co_2-N-HCS-900. 33

Figure S29. (a) TEM, (b and c) HRTEM, (d) SAED, and (e-i) HAADF-STEM and C, O, N, Co elemental mapping images for the Co_{NP}/HCS-900. 34

Figure S30. SAED pattern of the Co_{SA}-N-HCS-900. 35

Figure S31. SAED pattern of the Co_2-N-HCS-900. 36

Figure S32. AC HAADF-STEM images of Co_{NP}/N-HCS-300. 37

Figure S33. Statistical 50 pairs Co-Co distance for the Co_{SA}-N-HCS-900. 38

Figure S34. Statistical 50 pairs Co-Co distance for the Co_2-N-HCS-900. 39

Figure S35. AC HAADF-STEM image of Co_2-N-HCS-900 (pores marked by green cycles). 40

Figure S36. AC HAADF-STEM and corresponding statistical M-M distance for (a) Al_2-N-HCS-900, (b) Ca_2-N-HCS-900, (c) Cr_2-N-HCS-900, (d) Mn_2-N-HCS-900, (e) Fe_2-N-HCS-900, (f) Ni_2-N-HCS-900, (g) Cu_2-N-HCS-900, (h) Zn_2-N-HCS-900, (i) Ru_2-N-HCS-900, (j) Sb_2-N-HCS-900, (k) Ce_2-N-HCS-900, (l) Bi_2-N-HCS-900, (m) CoFe-N-HCS-900, (n) CoNi-N-HCS-900, (o) CoCu-N-HCS-900, (p) CoZn-N-HCS-900, (q) CoMn-N-HCS-900, (r) FeNi-N-HCS-900, (s) FeCu-N-HCS-900, (t) FeZn-N-HCS-900, and (u) FeMn-N-HCS-900. 41

Figure S37. k^3-weighted FT-EXAFS spectra at Co K-edge for Co foil, CoO, CoPc, Co_{SA}-N-HCS-900, and Co_2-N-HCS-900. 42

Figure S38. The possible Co atomic structures optimized by DFT (green ball: Co; blue ball: N; purple ball: C; red ball: O). 43

Figure S39. Comparison between the Co K-edge XANES experimental spectrum of Co_{SA}-N-HCS-900 (solid blue line) and the theoretical spectrum (solid red line) calculated with different Co atomic structures (green ball: Co; blue ball: N; purple ball: C; red ball: O). 44

Figure S40. Comparison between the Co K-edge EXAFS experimental spectrum of Co_{SA}-N-HCS-900 (solid black line) and the theoretical spectrum (solid red line) calculated with different Co atomic structures (green ball: Co; blue ball: N; purple ball: C; red ball: O). 45

Figure S41. Comparison between the Co K-edge XANES experimental spectrum of Co_2-N-HCS-900 (solid blue line) and the theoretical spectrum (solid red line) calculated with different Co atomic structures (green ball: Co; blue ball: N; purple ball: C; red ball: O). 46

Figure S42. Comparison between the Co K-edge EXAFS experimental spectrum of Co_2-N-HCS-900 (solid black line) and the theoretical spectrum (solid red line) calculated with different Co atomic structures. 47

Figure S43. (a) XPS survey spectra and (b) the corresponding N content for Co_{NP}/HCS-900, Co_{SA}-N-HCS-900, and Co_2-N-HCS-900. 48

Figure S44. C 1*s* high-resolution XPS spectra of (a) Co_{NP}/HCS-900, (b) Co_{SA}-N-HCS-900, and (c) Co_2-N-HCS-900. 49

Figure S45. N 1*s* high-resolution XPS spectra of (a) Co_{NP}/HCS-900, (b) Co_{SA}-N-HCS-900, and (c) Co_2-N-HCS-900. 50

Figure S46. The tendency of (a) C-N bond, (b) pyridinic-N and Co-N_x from Co_{NP}/HCS-900 to Co_{SA}-N-HCS-900 and Co_2-N-HCS-900. 51

Figure S47. Raman spectra for the (a) Co_{NP}/HCS-900, (b) Co_{SA}-N-HCS-900, and (c) Co_2-N-HCS-900. 52

Figure S48. (a) Nitrogen adsorption-desorption isotherms and (b) pore size distribution for the Co_{NP}/HCS-900, Co_{SA}-N-HCS-900, and Co_2-N-HCS-900. 53

Figure S49. Co 2*p* high-resolution XPS spectra of (a) Co_{NP}/HCS-900, (b) Co_{SA}-N-HCS-900, and (c) Co_2-N-HCS-900 54

Figure S50. O 1*s* high-resolution XPS spectra of (a) Co_{NP}/HCS-900, (b) Co_{SA}-N-HCS-900, and (c) Co_2-N-HCS-900. 55

Figure S51. M-T curve of the Co_{NP}/HCS-900. 56

Figure S52. UPS for the Co_{NP}/HCS-900, Co_{SA}-N-HCS-900, and Co_2-N-HCS-900. 57

Figure S53. The valence-band spectrum for the Co_{NP}/HCS-900, Co_{SA}-N-HCS-900, and Co_2-N-HCS-900 58

Figure S54. The corresponding E_{onset} and $E_{1/2}$ for the HCS-900, Co_{NP}/HCS-900, Co_{SA}-N-HCS-900, Co_2-N-HCS-900, and commercial Pt/C. 59

Figure S55. The corresponding j_k for the HCS-900, Co_{NP}/HCS-900, Co_{SA}-N-HCS-900, Co_2-N-HCS-900, and commercial Pt/C at 0.90 V and 0.85 V. 60

Figure S56. Cyclic voltammetry (CV) curves for (a) HCS-900, (b) Co_{NP}/HCS-900, (c) Co_{SA}-N-HCS-900, and (d) Co_2-N-HCS-900 in O_2-saturated 0.1 M KOH solution. 61

Figure S57. The value of C_{dl} for the HCS-900, Co_{NP}/HCS-900, Co_{SA}-N-HCS-900, and Co_2-N-HCS-900 in O_2-saturated 0.1 M KOH solution. 62

Figure. S58. Mass activity toward ORR for the Co_{NP}/HCS-900, Co_{SA}-N-HCS-900, Co_2-N-HCS-900 and commercial Pt/C. 63

Figure S59. (a) LSV curves of Co_2-N-HCS-900 at different rotation rates, (b) K-L plots, (c) RRDE curves, and (d) the HO_2^- yield and n in O_2-saturated 0.1 M KOH solution. 64

Figure S60. Mass activity toward OER for the Co_{NP}/HCS-900, Co_{SA}-N-HCS-900, Co_2-N-HCS-900 and commercial RuO_2. 65

Figure S61. Mass activity toward HER for the Co_{NP}/HCS-900, Co_{SA}-N-HCS-900, Co_2-N-HCS-900 and commercial Pt/C. 66

Figure S62. Relative current-density-time curves of Co_2-N-HCS-900 and Pt/C at 0.7 V with a rotation speed of 200 rpm in O_2-saturated 0.1 M KOH solution. 67

Figure S63. Durability tests for OER at 10 mA cm^{-2} in 1 M KOH solution. 68

Figure S64. Durability tests for HER at 10 mA cm^{-2} in 1 M KOH solution. 69

Figure S65. Galvanostatic discharge-charge cycling curves for ZABs driven by Co_2-N-HCS-900 at current density of 5 mA cm^{-2}. 70

Figure S66. Galvanostatic discharge-charge cycling curves for ZABs using Co_2-N-HCS-900 as the air cathode at (a) 0-1 h, (b) 299-300 h, (c) 599-600 h, and (d) 799-800 h. 71

Figure S67. Galvanostatic discharge-charge cycling curves for ZABs driven by Co_2-N-HCS-900 at current density of 50 mA cm^{-2}. 72

Figure S68. (a) Open-circuit potential plots of one or two or three tandem Zn-air batteries driven by Co_2-N-HCS-900, and (b-d) Photograph of one or two or three tandem ZABs driven by Co_2-N-HCS-900 displaying a measured open-circuit voltage of ~1.46, 2.91, and 4.35 V. 73

Figure S69. Photograph of series WSS driven by Co_2-N-HCS-900 during the day and night over 48 h. 74

Table S1. EXAFS fitting parameters at the Co K-edge for various samples (S_0^2= 0.776). 75

Table S2. The XANES and EXAFS fitting parameters at the Co K-edge of the possible structures from model 1 to model 10 for the Co_{SA}-N-HCS-900 76

Table S3. The XANES and EXAFS fitting parameters at the Co K-edge of the possible structures from model 1 to model 10 for the Co_2-N-HCS-900 77

Table S4. Comparison of alkaline ORR performances between Co_2-N-HCS-900 and other M-N-C materials in the literatures 78

Table S5. Comparison of alkaline OER performances between Co_2-N-HCS-900 and other M-N-C materials in the literatures 79

Table S6. Comparison of alkaline HER performances between Co_2-N-HCS-900 and other M-N-C materials in the literatures 80

Table S7. Comparison of trifunctional electrocatalytic performance between Co_2-N-HCS-900 and other M-N-C materials in the literatures. 81

Table S8. Comparison of Zn-air batteries performances between Co_2-N-HCS-900 and other M-N-C materials in the literatures 82

Supplemental Figures and Tables

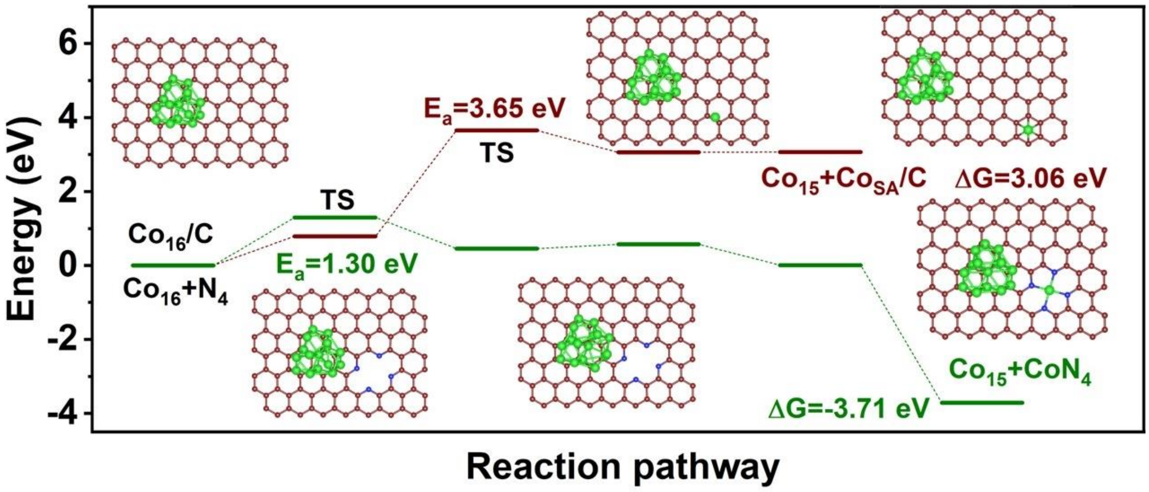

Figure S1. Calculated relative energies along the stretching pathway of the Co atom from Co_{16} to Co_{SA}/C or CoN_4 model by CI-NEB.

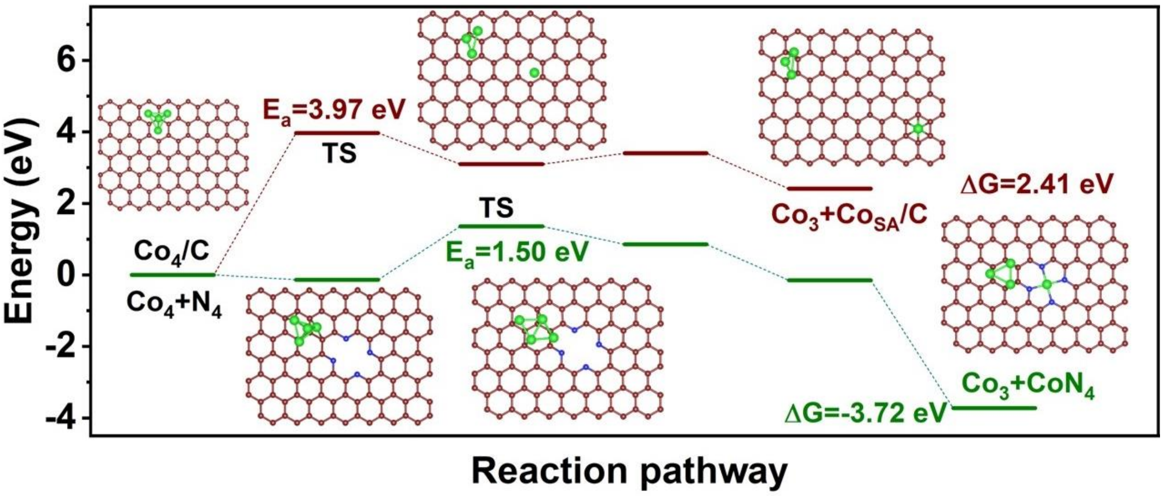

Figure S2. Calculated relative energies along the stretching pathway of the Co atom from Co_4 to Co_{SA}/C or CoN_4 model by CI-NEB.

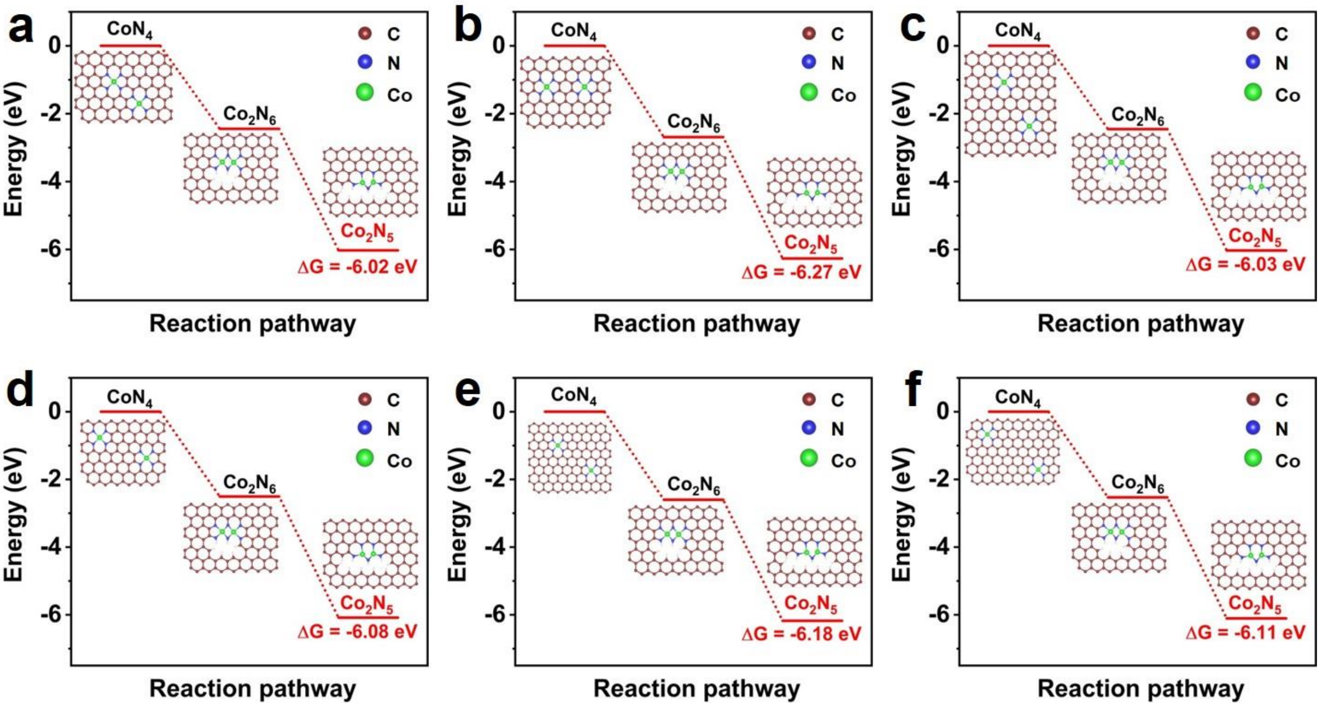

Figure S3. (a-f) Calculated relative energies of different CoN_4 models that are separated by several carbon atoms, Co_2N_6, and Co_2N_5 models.

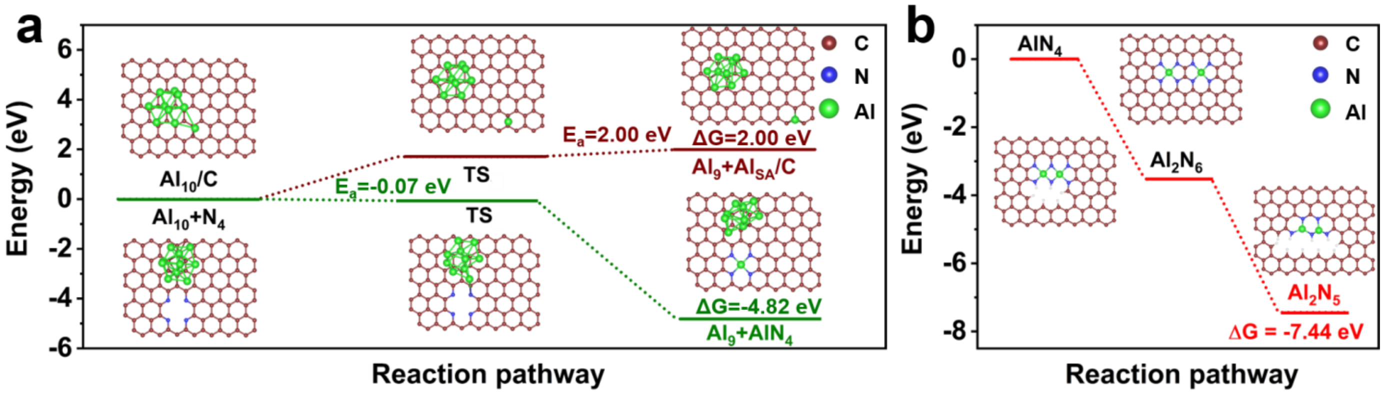

Figure S4. (a) Calculated relative energies along the stretching pathway of the Al atom from Al_{10} to Al_{SA}/C or AlN_4 model by CI-NEB. (b) Calculated relative energies of AlN_4, Al_2N_6, and Al_2N_5 models.

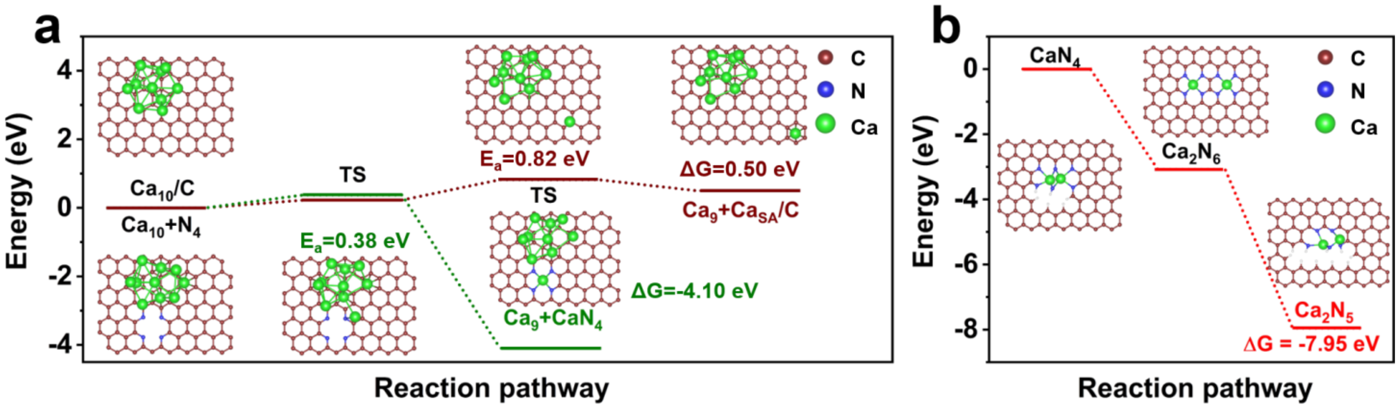

Figure S5. (a) Calculated relative energies along the stretching pathway of the Ca atom from Ca_{10} to Ca_{SA}/C or CaN_4 model by CI-NEB. (b) Calculated relative energies of CaN_4, Ca_2N_6, and Ca_2N_5 models.

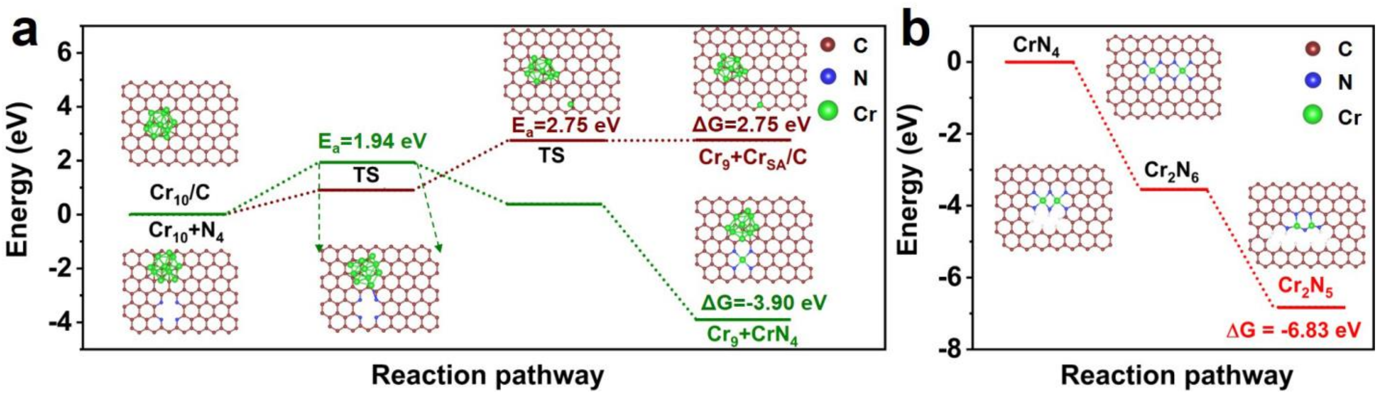

Figure S6. (a) Calculated relative energies along the stretching pathway of the Cr atom from Cr_{10} to Cr_{SA}/C or CrN_4 model by CI-NEB. (b) Calculated relative energies of CrN_4, Cr_2N_6, and Cr_2N_5 models.

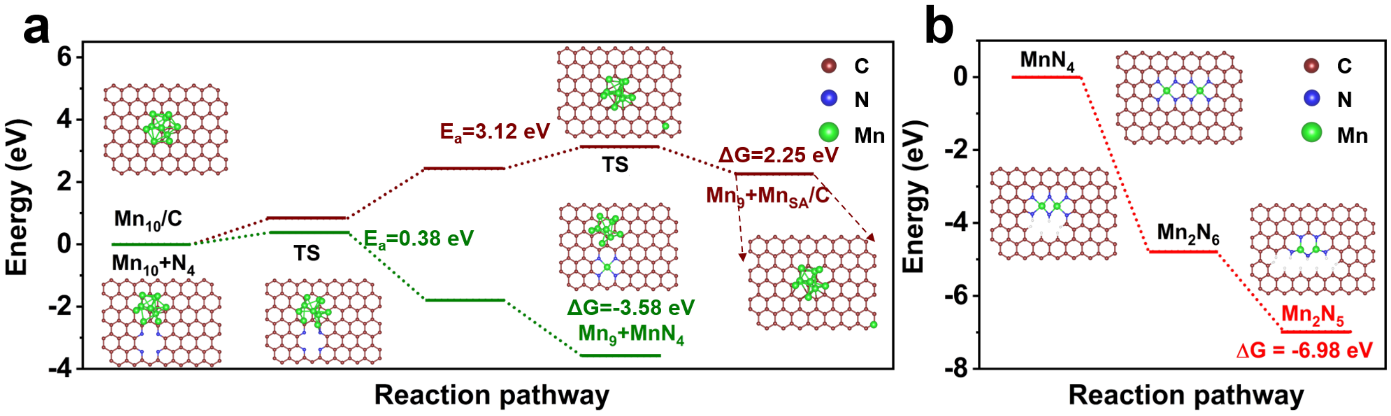

Figure S7. (a) Calculated relative energies along the stretching pathway of the Mn atom from Mn_{10} to Mn_{SA}/C or MnN_4 model by CI-NEB. (b) Calculated relative energies of MnN_4, Mn_2N_6, and Mn_2N_5 models.

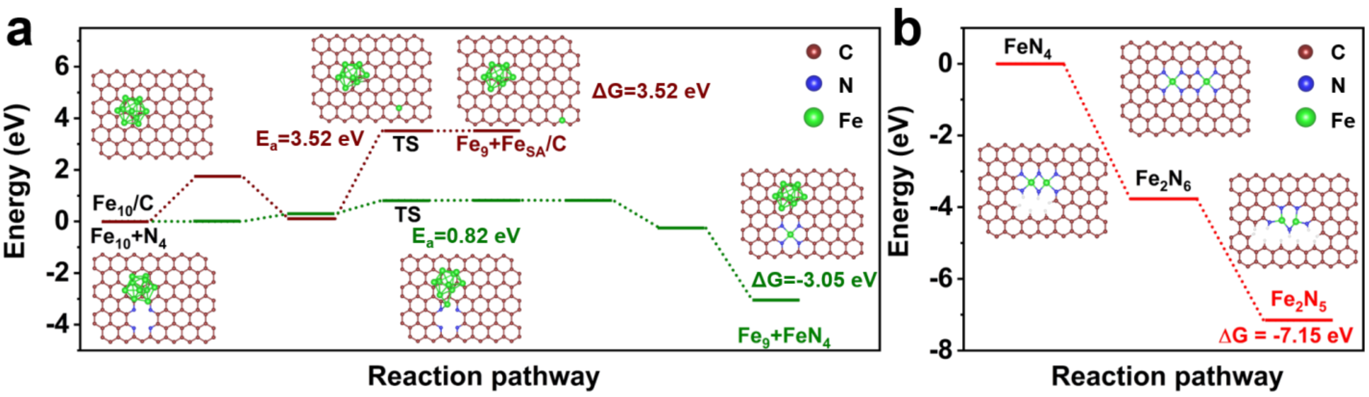

Figure S8. (a) Calculated relative energies along the stretching pathway of the Fe atom from Fe_{10} to Fe_{SA}/C or FeN_4 model by CI-NEB. (b) Calculated relative energies of FeN_4, Fe_2N_6, and Fe_2N_5 models.

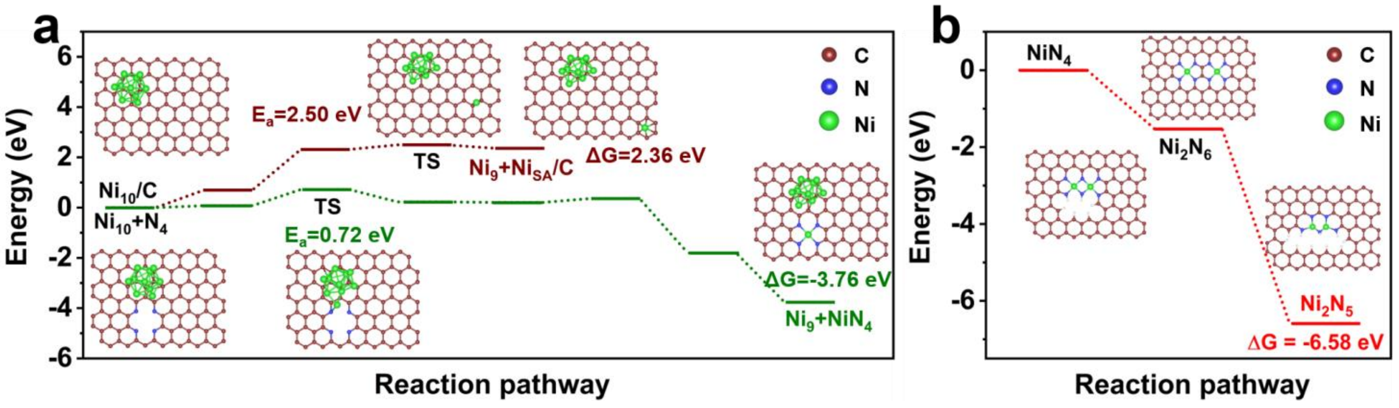

Figure S9. (a) Calculated relative energies along the stretching pathway of the Ni atom from Ni_{10} to Ni_{SA}/C or NiN_4 model by CI-NEB. (b) Calculated relative energies of NiN_4, Ni_2N_6, and Ni_2N_5 models.

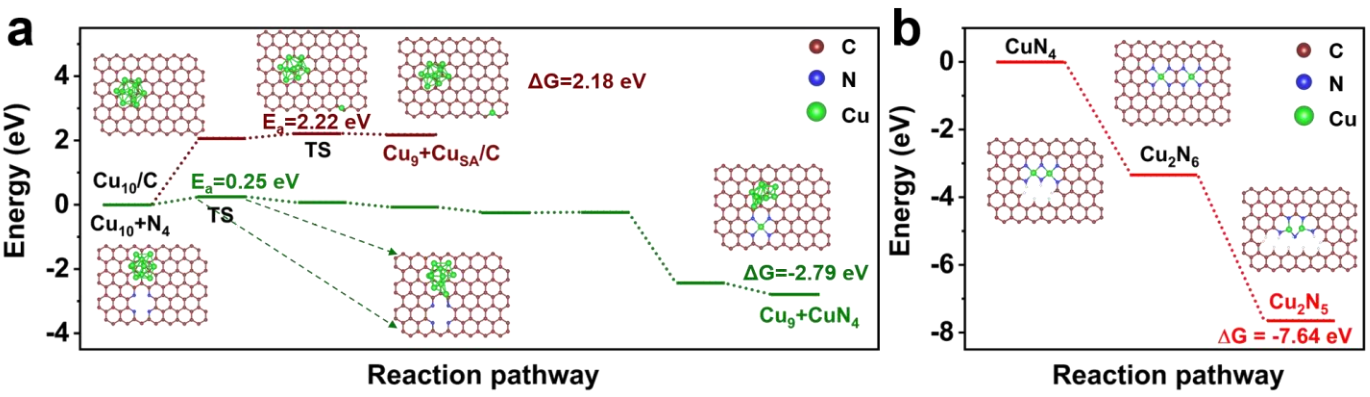

Figure S10. (a) Calculated relative energies along the stretching pathway of the Cu atom from Cu_{10} to Cu_{SA}/C or CuN_4 model by CI-NEB. (b) Calculated relative energies of CuN_4, $Cu_2N_{6,}$ and Cu_2N_5 models.

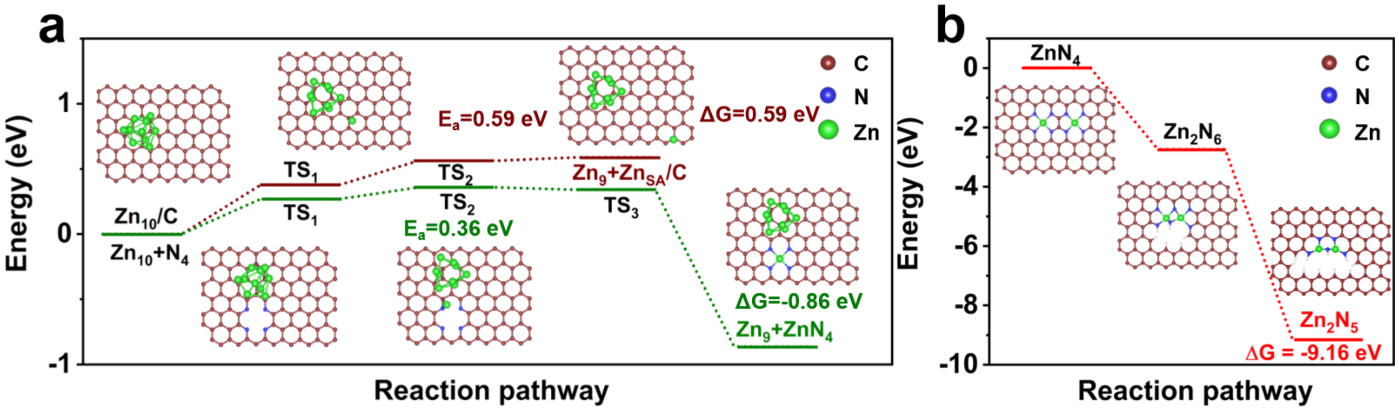

Figure S11. (a) Calculated relative energies along the stretching pathway of the Zn atom from Zn_{10} to Zn_{SA}/C or ZnN_4 model by CI-NEB. (b) Calculated relative energies of ZnN_4, Zn_2N_6, and Zn_2N_5 models.

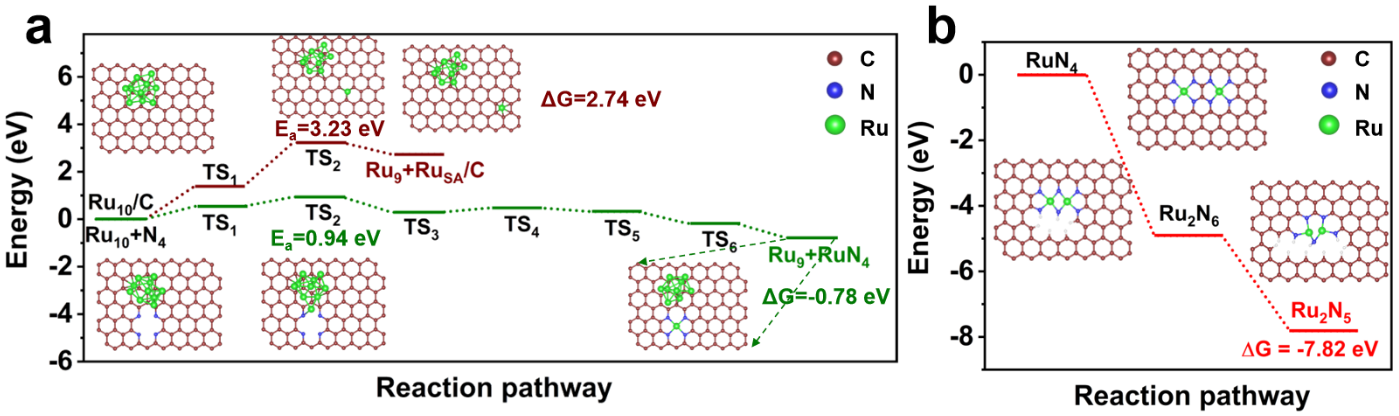

Figure S12. (a) Calculated relative energies along the stretching pathway of the Ru atom from Ru_{10} to Ru_{SA}/C or RuN_4 model by CI-NEB. (b) Calculated relative energies of RuN_4, Ru_2N_6, and Ru_2N_5 models.

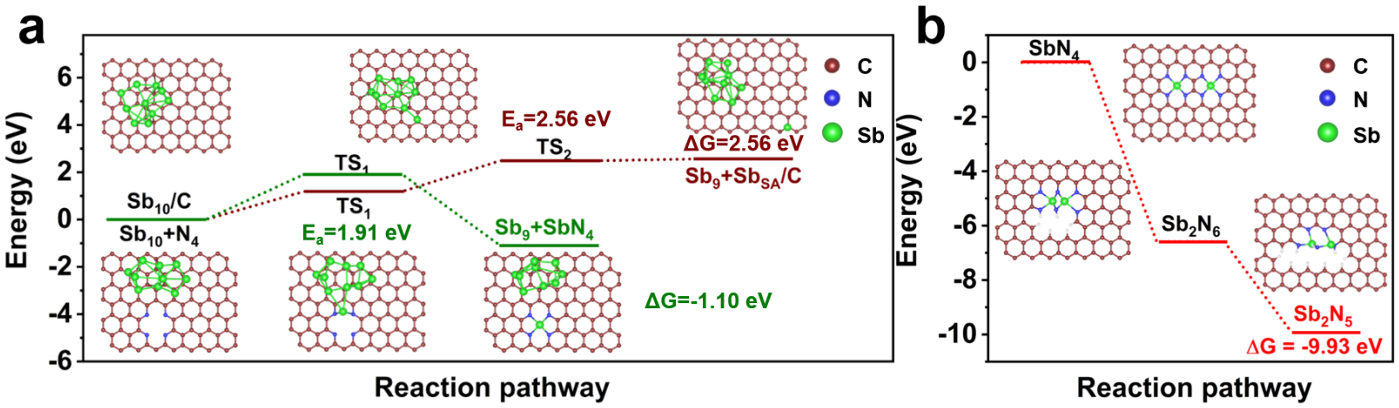

Figure S13. (a) Calculated relative energies along the stretching pathway of the Sb atom from Sb_{10} to Sb_{SA}/C or SbN_4 model by CI-NEB. (b) Calculated relative energies of SbN_4, Sb_2N_6, and Sb_2N_5 models.

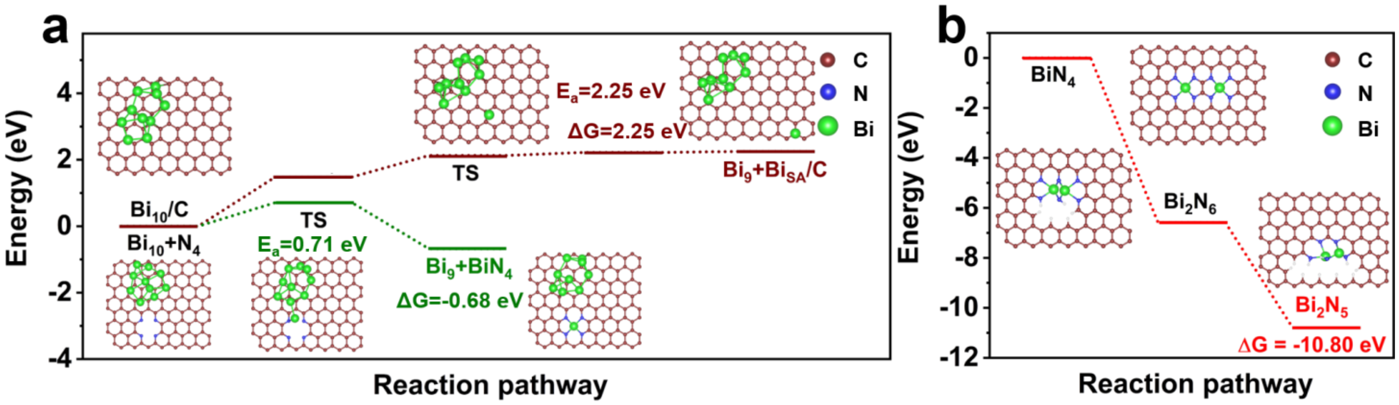

Figure S14. (a) Calculated relative energies along the stretching pathway of the Bi atom from Bi_{10} to Bi_{SA}/C or BiN_4 model by CI-NEB. (b) Calculated relative energies of BiN_4, Bi_2N_6, and Bi_2N_5 models.

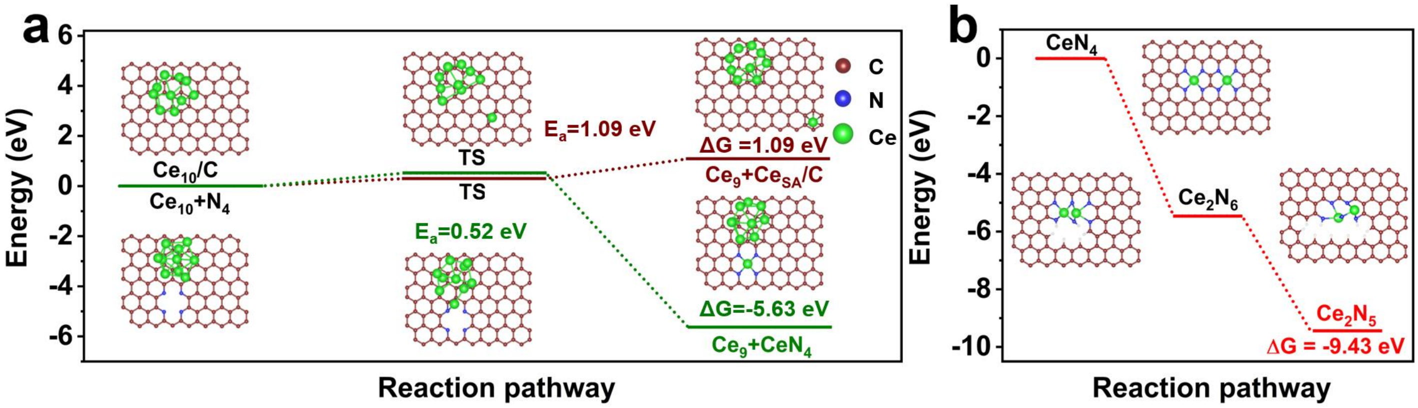

Figure S15. (a) Calculated relative energies along the stretching pathway of the Ce atom from Ce_{10} to Ce_{SA}/C or CeN_4 model by CI-NEB. (b) Calculated relative energies of CeN_4, Ce_2N_6, and Ce_2N_5 models.

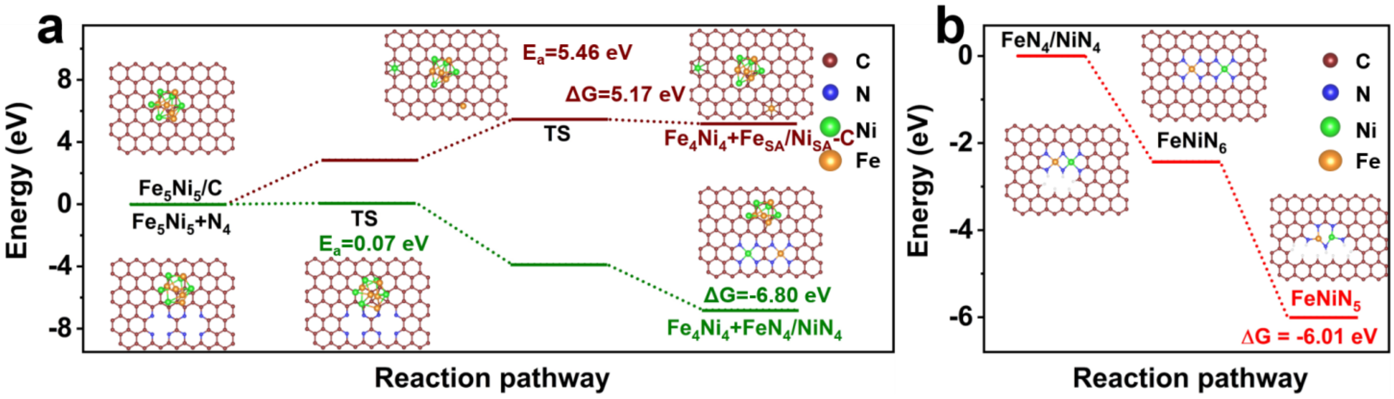

Figure S16. (a) Calculated relative energies along the stretching pathway of the Fe/Ni atom from Fe_5Ni_5 to Fe_{SA}/Ni_{SA}-C or FeN_4/NiN_4 model by CI-NEB. (b) Calculated relative energies of FeN_4/NiN_4, $FeNiN_6$, and $FeNiN_5$ models.

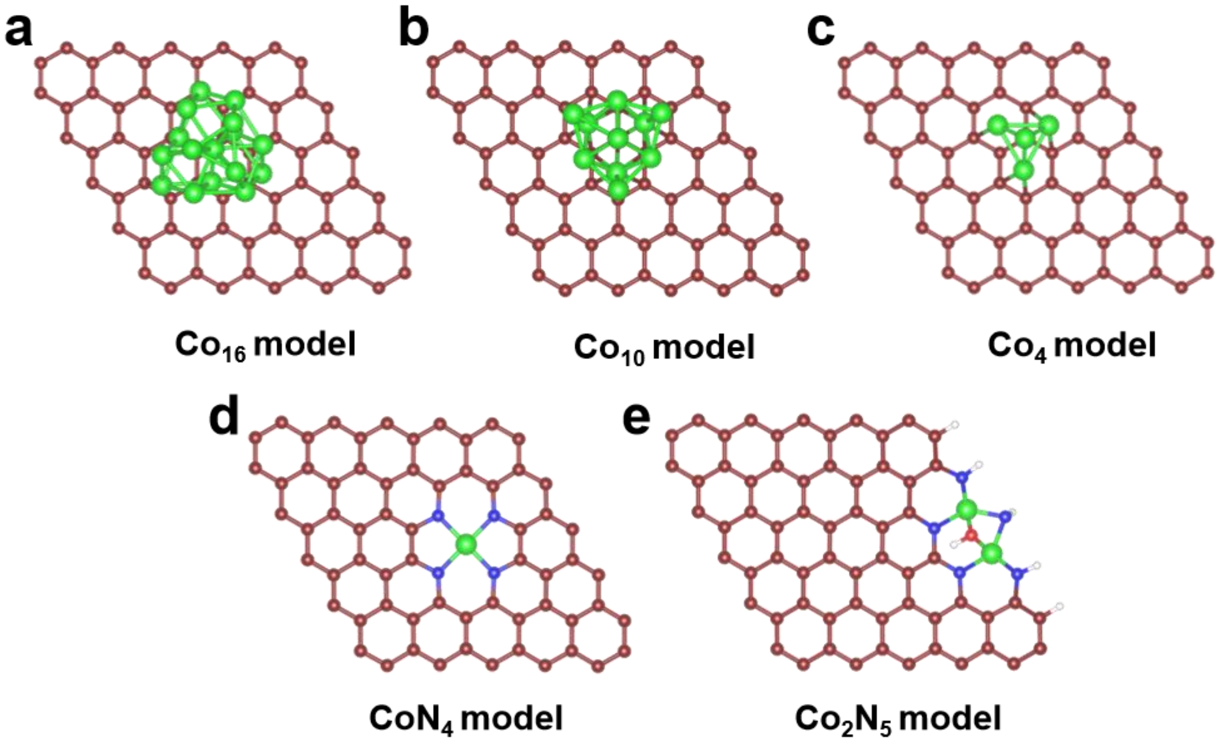

Figure S17. Optimized atomic configurations of (a) Co_{16}, (b) Co_{10}, (c) Co_4, (d) CoN_4 and (e) Co_2N_5 model.

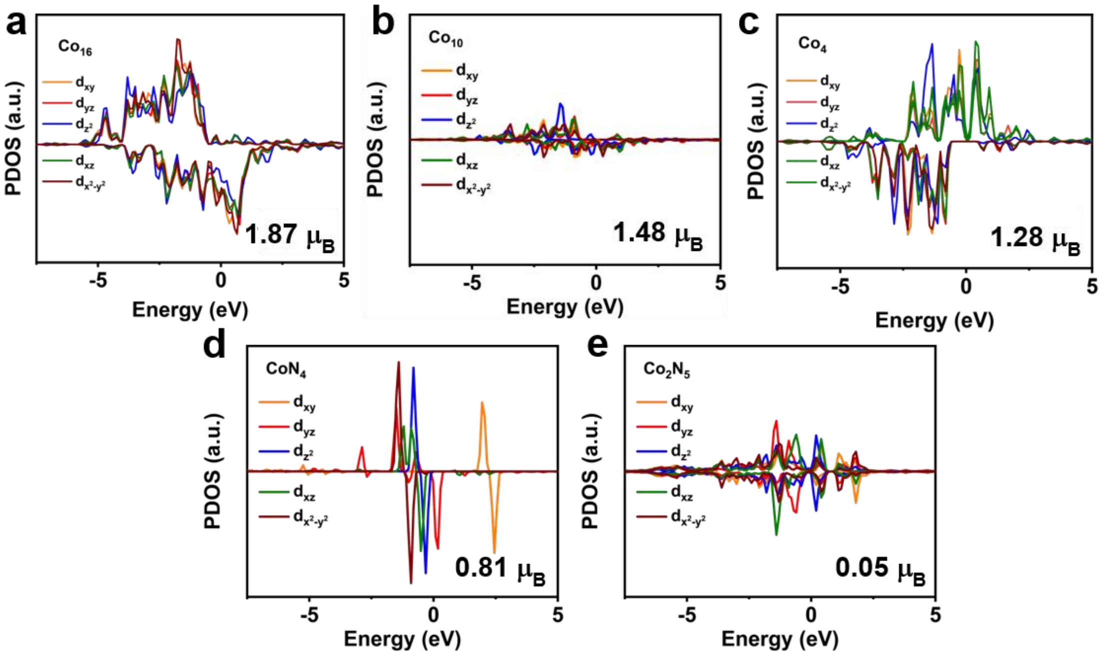

Figure S18. PDOS of the (a) Co_{16}, (b) Co_{10}, (c) Co_4, (d) CoN_4, and (e) Co_2N_5 models.

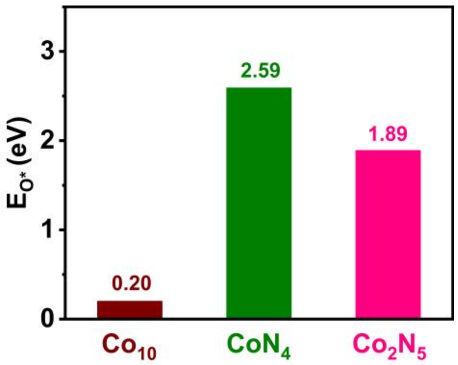

Figure S19. Calculated adsorption free energies of O* intermediates on the Co_{10} nanoparticles, CoN_4 single atom, and Co_2N_5 dual atom models, respectively.

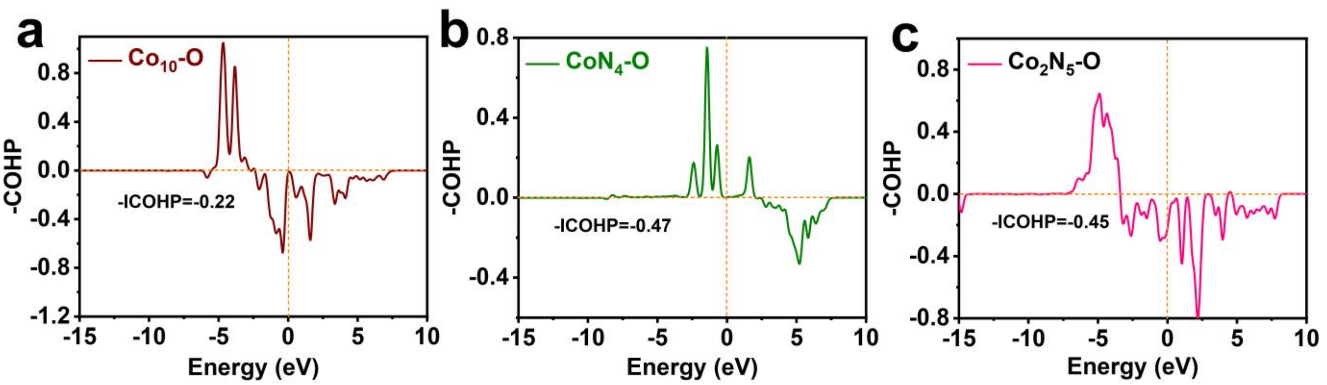

Figure S20. Crystal Orbital Hamilton Population (COHP) analysis corresponding integrated-COHP (-ICOHP) value of the Co_{10}-O (a), CoN_4-O (b) and Co_2N_5-O (c).

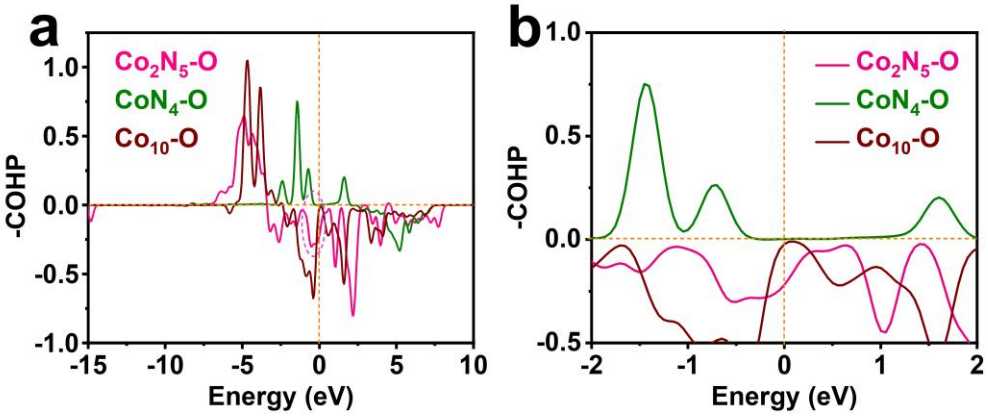

Figure S21. The COHP analysis (a) and the magnification around Fermi level image (b) of the Co_{10}-O, CoN_4-O and Co_2N_5-O.

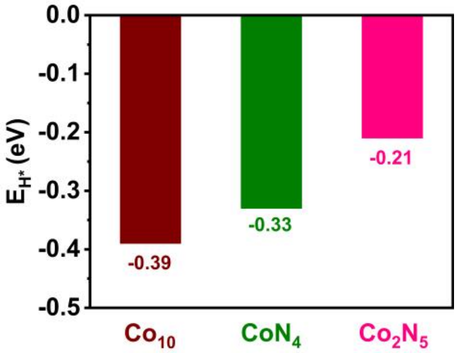

Figure S22. Calculated adsorption free energies of H* intermediates on the Co_{10} nanoparticles, CoN_4 single atom and Co_2N_5 dual atom models, respectively.

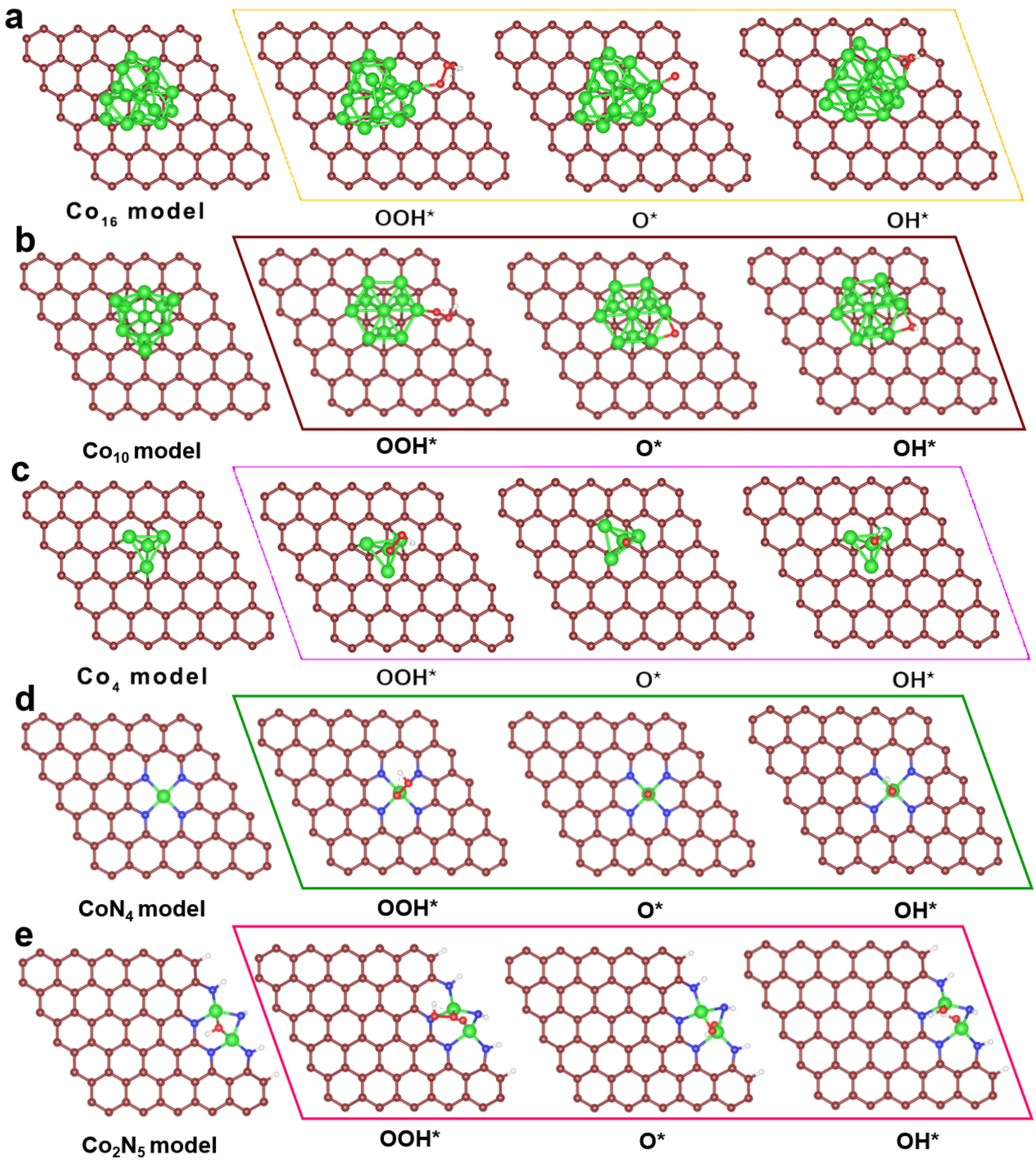

Figure S23. Optimized models and atomic configurations of oxygen-containing species (OOH*, O* and OH*) adsorbed on (a) Co_{16}, (b) Co_{10}, (c) Co_4, (d) CoN_4, and (e) Co_2N_5 models.

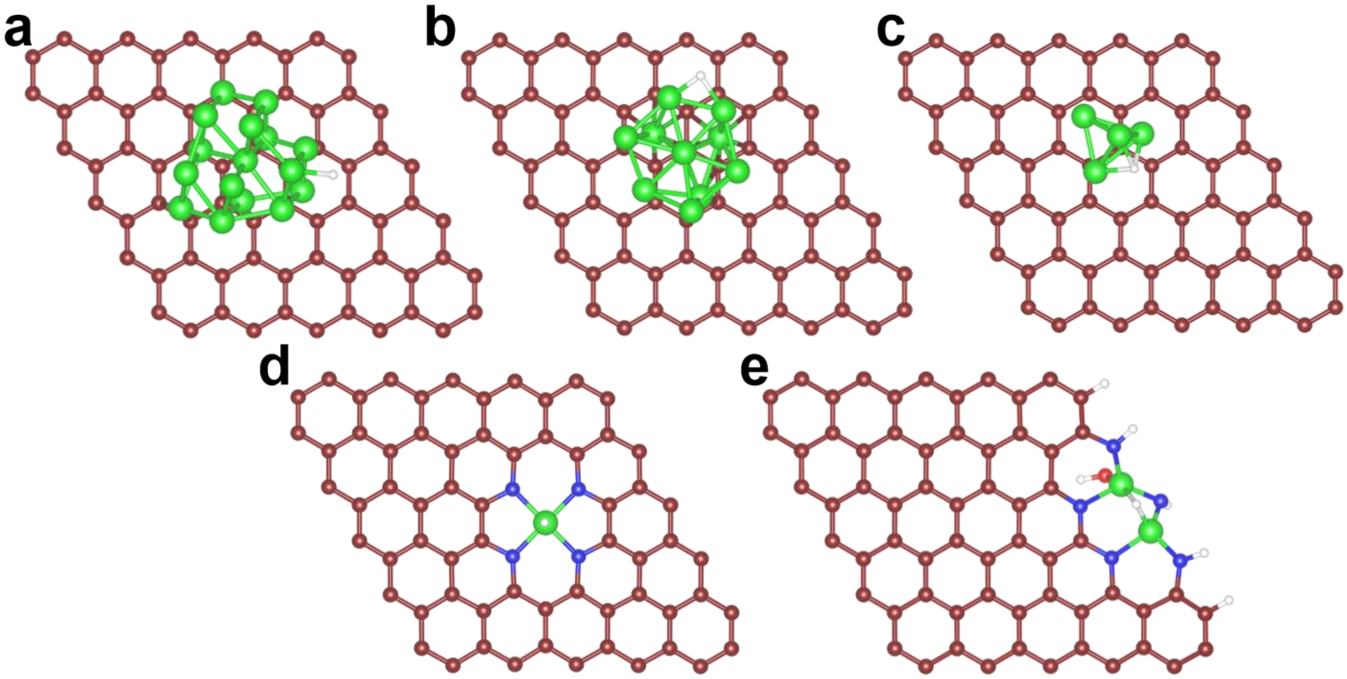

Figure S24. Optimized models and atomic configurations of H* species toward HER adsorbed on (a) Co_{16}, (b) Co_{10}, (c) Co_4, (d) CoN_4, and (e) Co_2N_5 models.

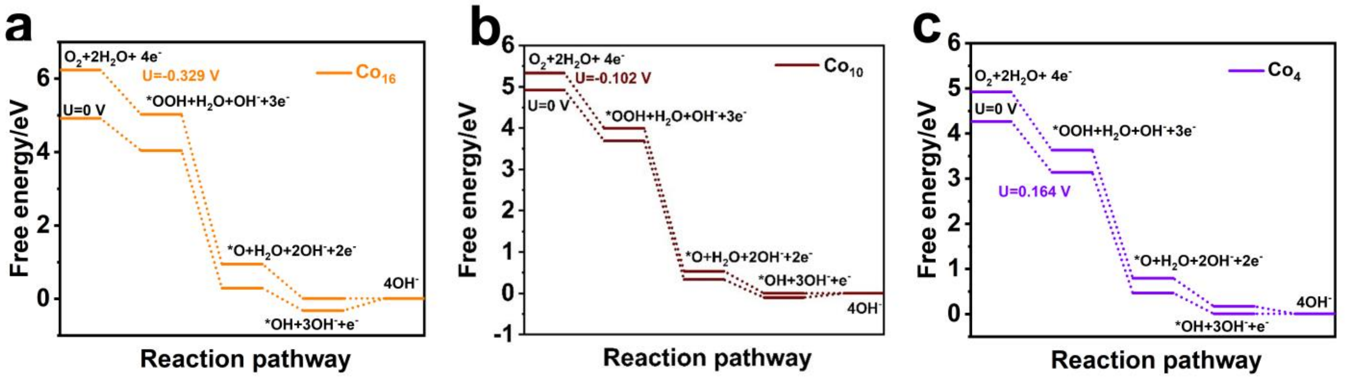

Figure S25. Free energy diagram of (a) Co_{16}, (b) Co_{10} and (c) Co_4 models for ORR.

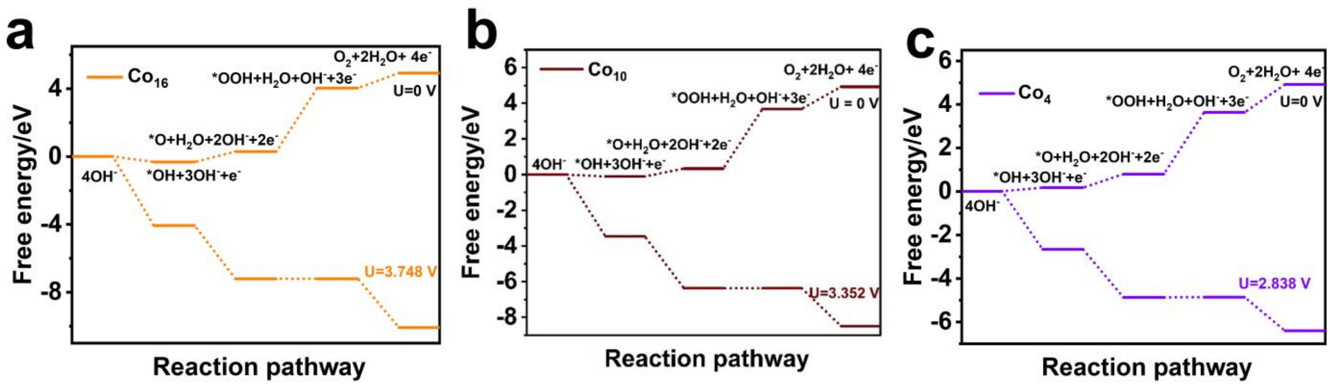

Figure S26. Free energy diagram of (a) Co_{16}, (b) Co_{10} and (c) Co_4 models for OER.

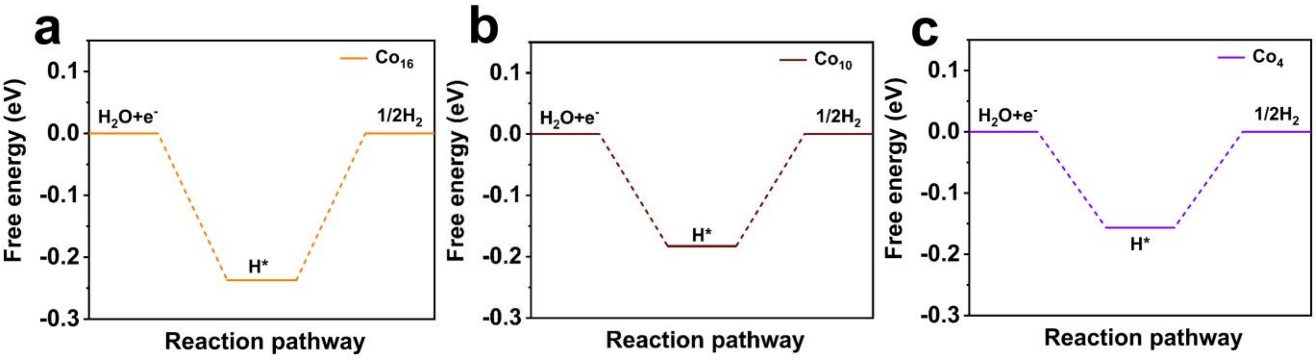

Figure S27. Free energy diagram of (a) Co_{16}, (b) Co_{10} and (c) Co_4 models for HER.

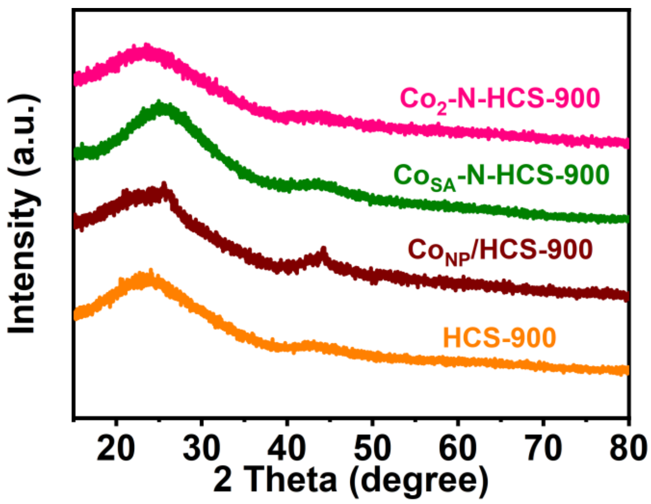

Figure S28. XRD patterns for HCS-900, Co_{NP}/HCS-900, Co_{SA}-N-HCS-900, and Co_2-N-HCS-900.

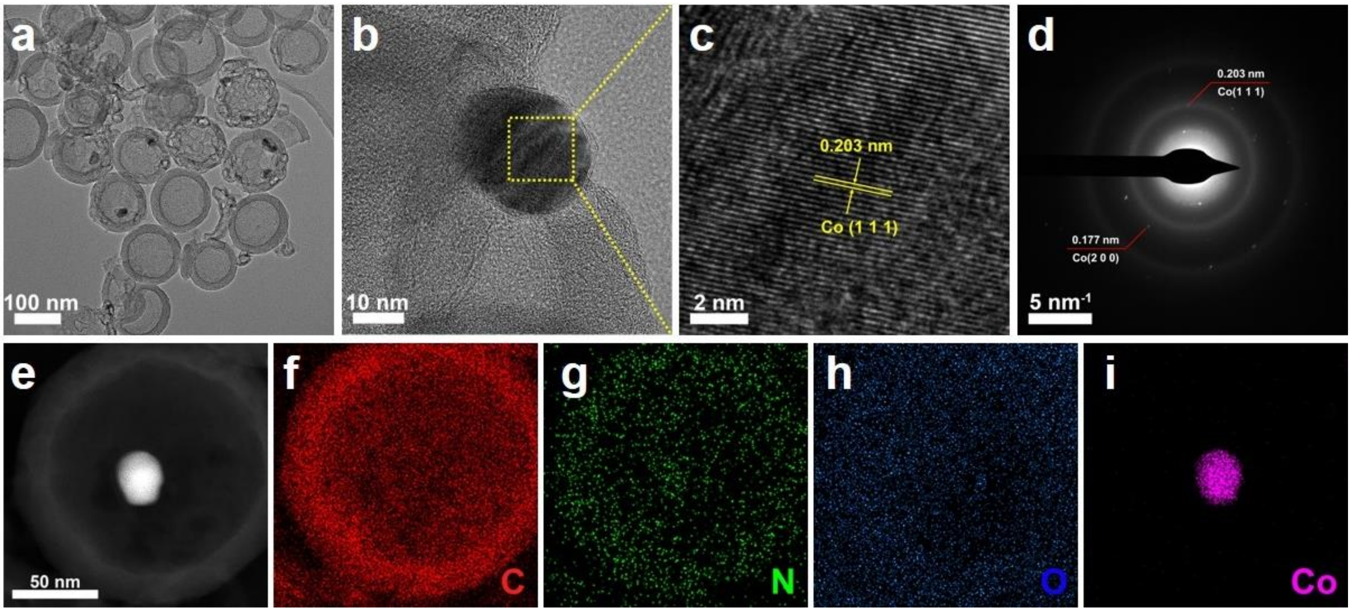

Figure S29. (a) TEM, (b and c) HRTEM, (d) SAED, and (e-i) HAADF-STEM and C, O, N, Co elemental mapping images for the Co_{NP}/HCS-900.

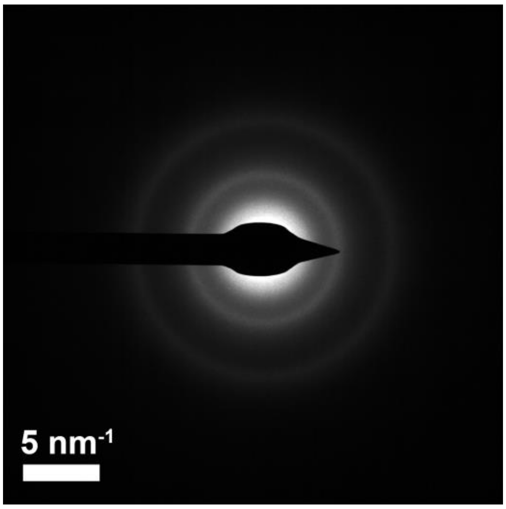

Figure S30. SAED pattern of the Co_{SA}-N-HCS-900.

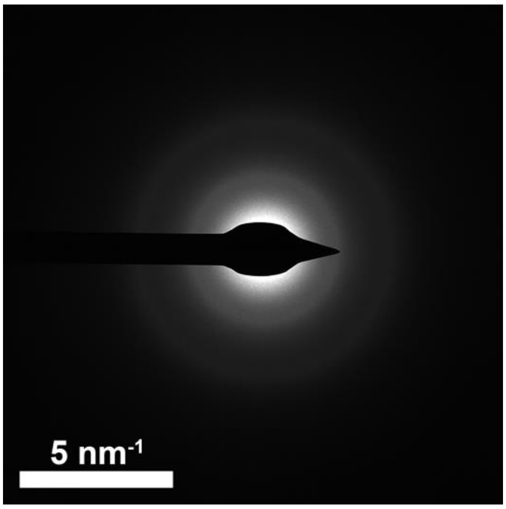

Figure S31. SAED pattern of the Co_2-N-HCS-900.

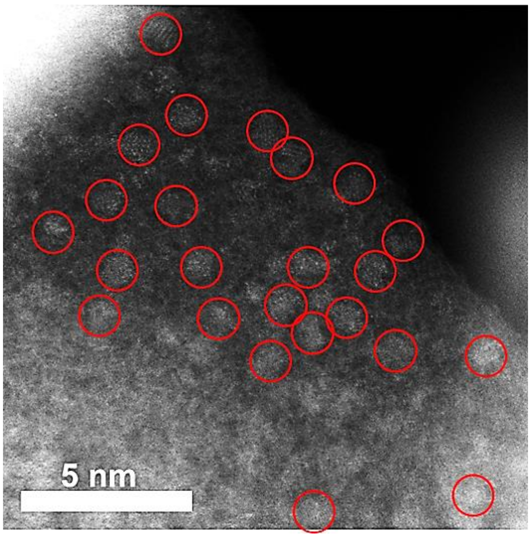

Figure S32. AC HAADF-STEM image of Co_{NP}/N-HCS-300.

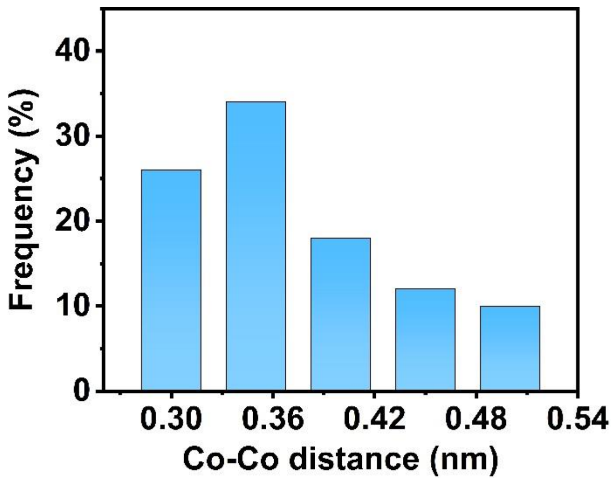

Figure S33. Statistical 50 pairs Co-Co distance for the Co_{SA}-N-HCS-900.

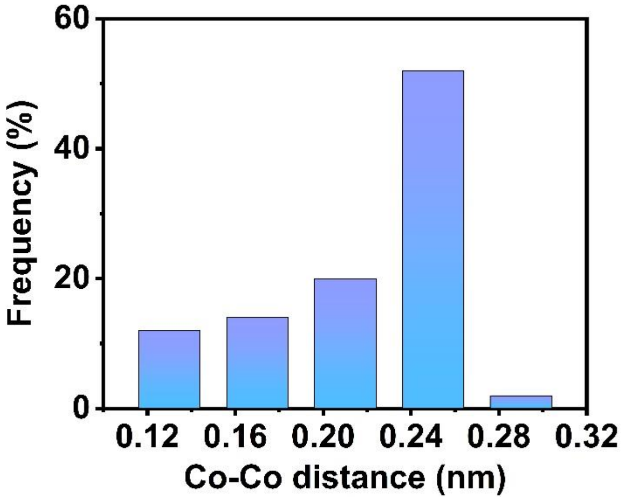

Figure S34. Statistical 50 pairs Co-Co distance for the Co_2-N-HCS-900.

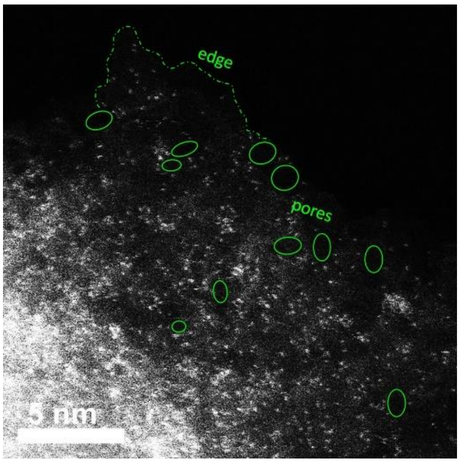

Figure S35. AC HAADF-STEM image of Co_2-N-HCS-900 (pores marked by green cycles).

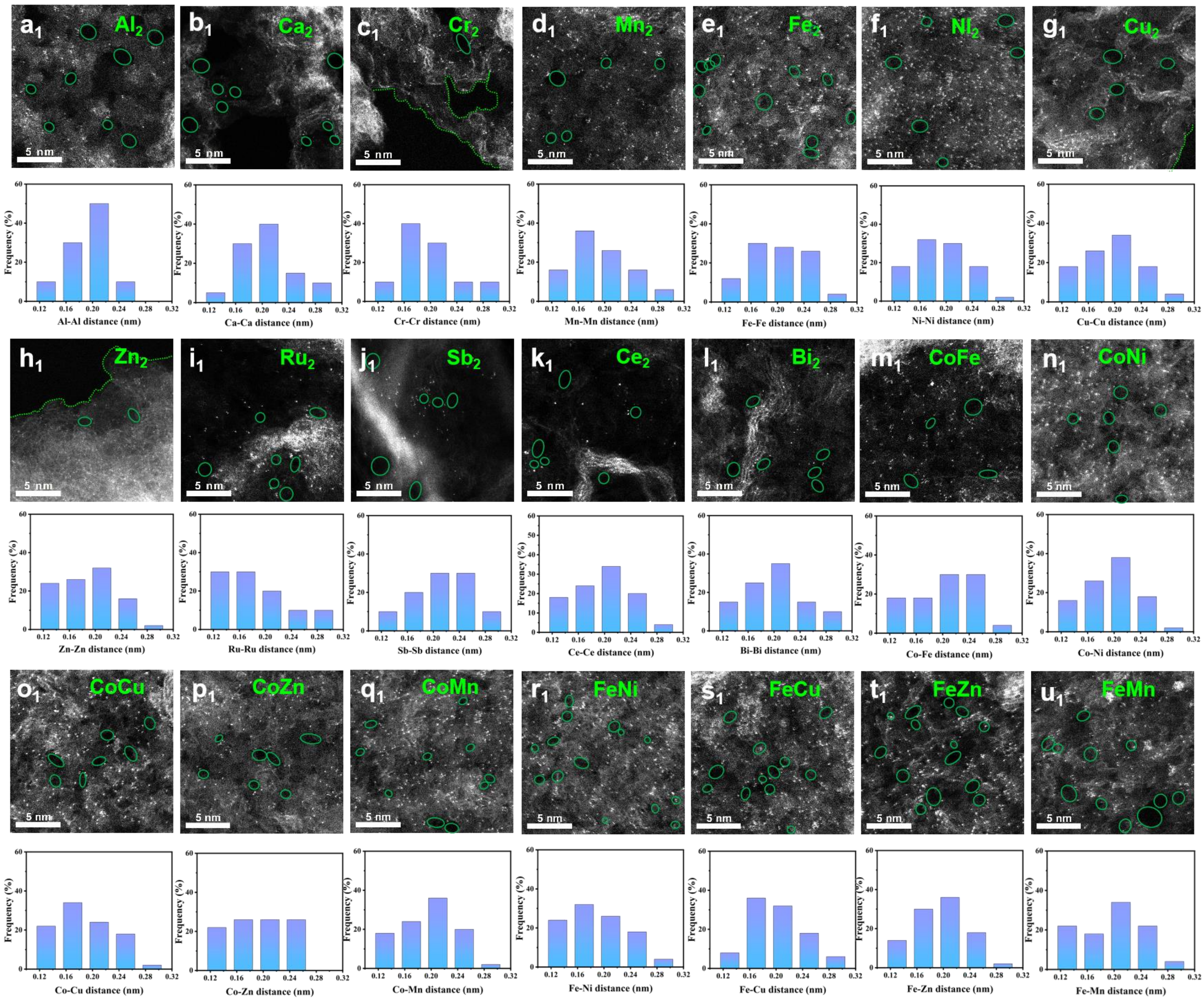

Figure S36. AC HAADF-STEM and corresponding statistical M-M distance for (a) Al_2-N-HCS-900, (b) Ca_2-N-HCS-900, (c) Cr_2-N-HCS-900, (d) Mn_2-N-HCS-900, (e) Fe_2-N-HCS-900, (f) Ni_2-N-HCS-900, (g) Cu_2-N-HCS-900, (h) Zn_2-N-HCS-900, (i) Ru_2-N-HCS-900, (j) Sb_2-N-HCS-900, (k) Ce_2-N-HCS-900, (l) Bi_2-N-HCS-900, (m) CoFe-N-HCS-900, (n) CoNi-N-HCS-900, (o) CoCu-N-HCS-900, (p) CoZn-N-HCS-900, (q) CoMn-N-HCS-900, (r) FeNi-N-HCS-900, (s) FeCu-N-HCS-900, (t) FeZn-N-HCS-900, and (u) FeMn-N-HCS-900.

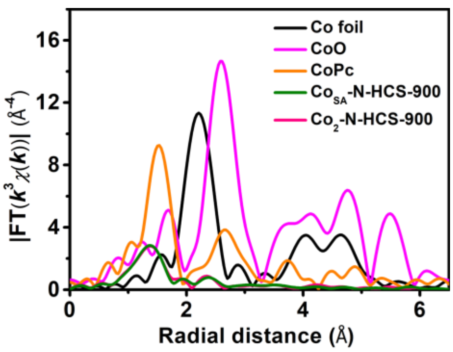

Figure S37. k^3-weighted FT-EXAFS spectra at Co K-edge for Co foil, CoO, CoPc, Co_{SA}-N-HCS-900, and Co_2-N-HCS-900.

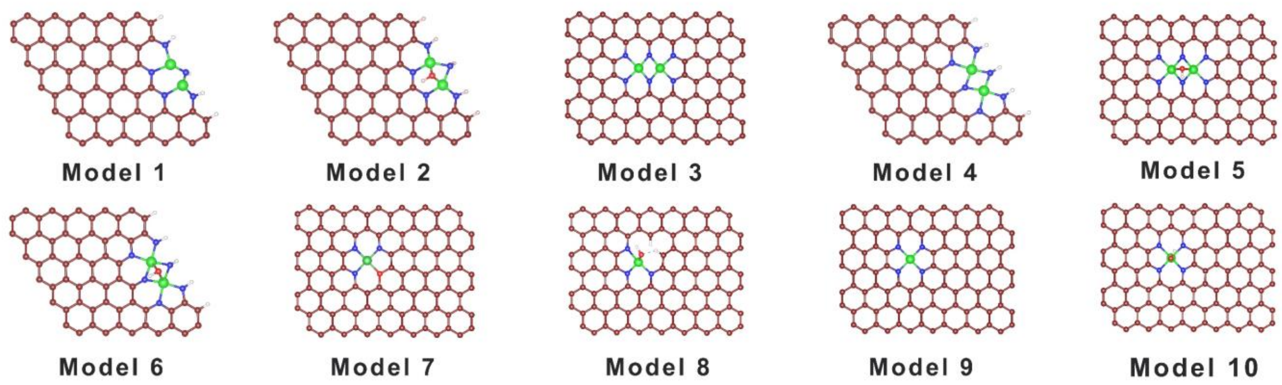

Figure S38. The possible Co atomic structures optimized by DFT (green ball: Co; blue ball: N; purple ball: C; red ball: O).

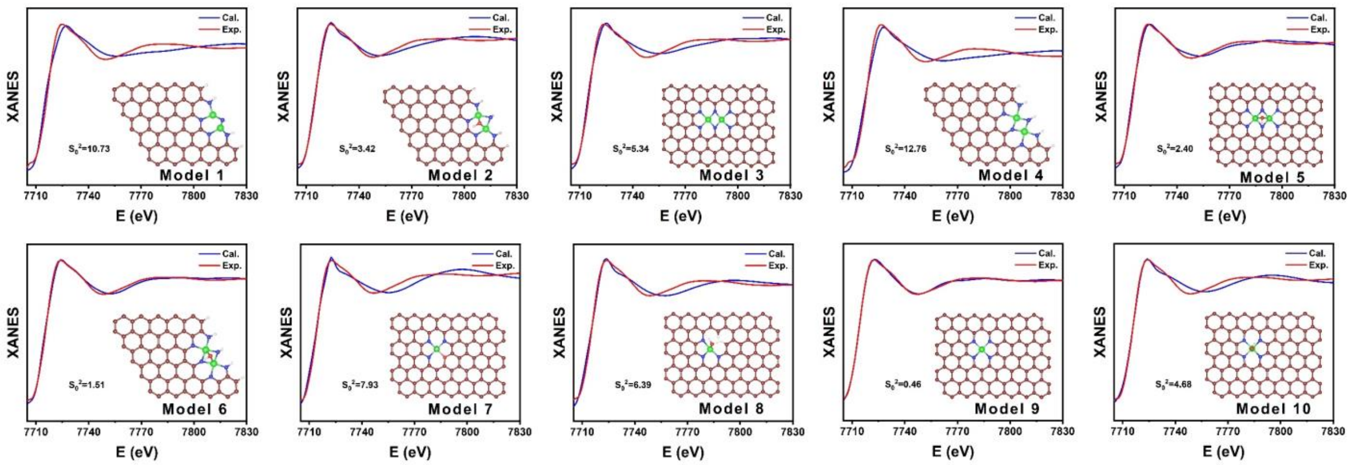

Figure S39. Comparison between the Co K-edge XANES experimental spectrum of Co_{SA}-N-HCS-900 (solid red line) and the theoretical spectrum (solid blue line) calculated with different Co atomic structures (green ball: Co; blue ball: N; purple ball: C; red ball: O).

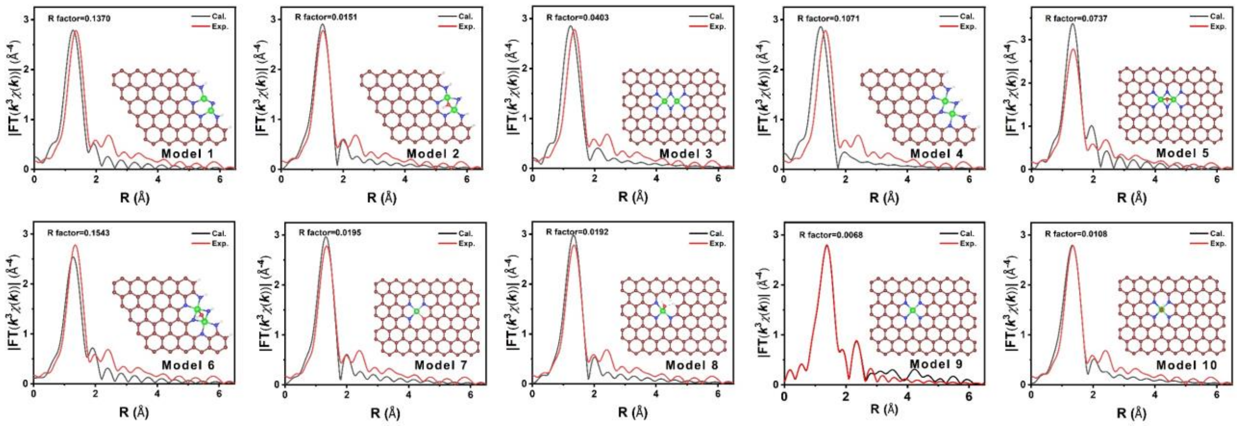

Figure S40. Comparison between the Co K-edge EXAFS experimental spectrum of Co_{SA}-N-HCS-900 (solid red line) and the theoretical spectrum (solid black line) calculated with different Co atomic structures (green ball: Co; blue ball: N; purple ball: C; red ball: O).

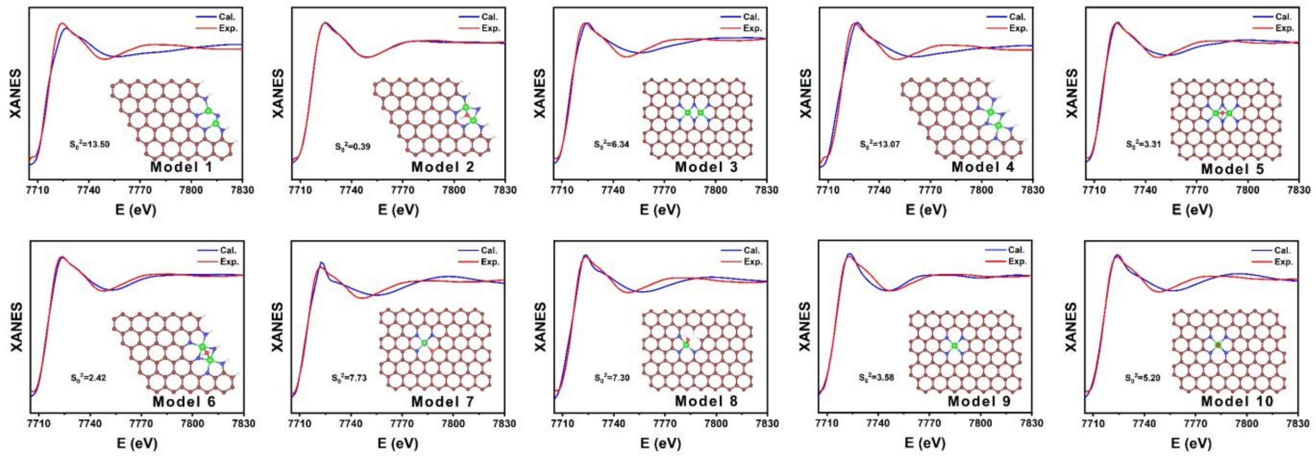

Figure S41. Comparison between the Co K-edge XANES experimental spectrum of Co_2-N-HCS-900 (solid red line) and the theoretical spectrum (solid blue line) calculated with different Co atomic structures (green ball: Co; blue ball: N; purple ball: C; red ball: O).

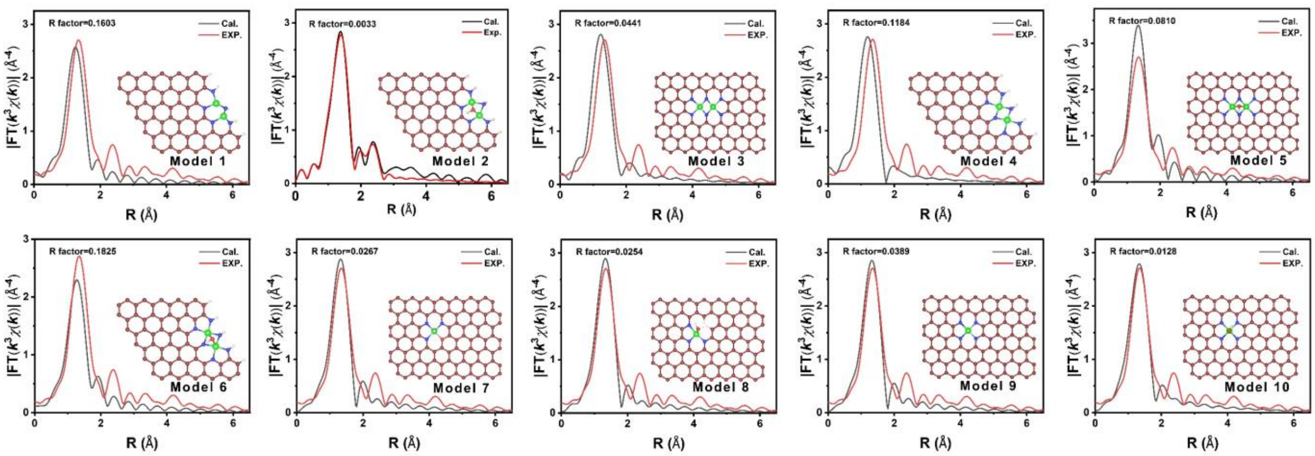

Figure S42. Comparison between the Co K-edge EXAFS experimental spectrum of Co_2-N-HCS-900 (solid red line) and the theoretical spectrum (solid black line) calculated with different Co atomic structures.

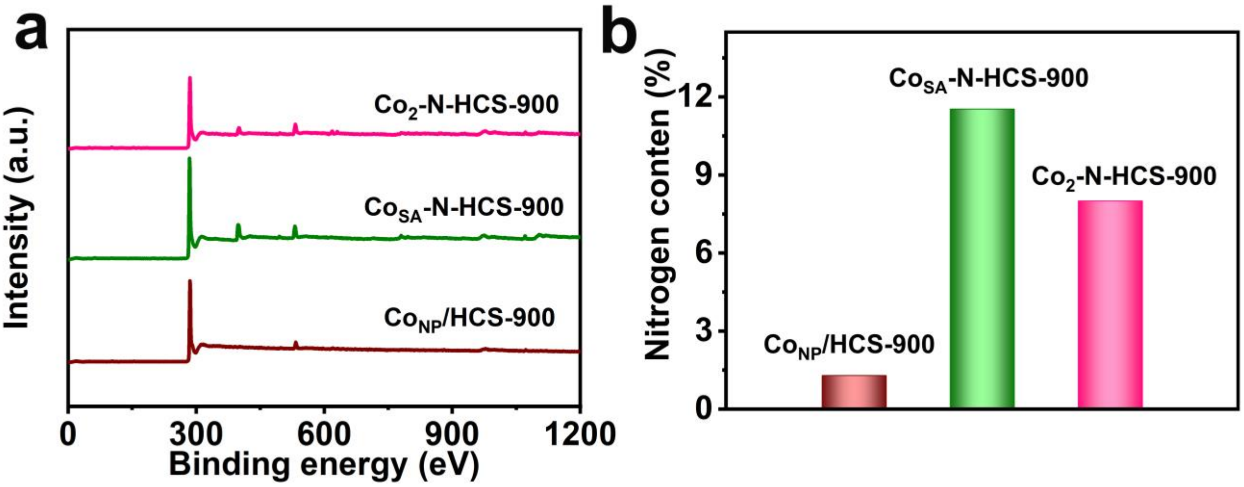

Figure S43. (a) XPS survey spectra and (b) the corresponding N content for Co_{NP}/HCS-900, Co_{SA}-N-HCS-900, and Co_2-N-HCS-900.

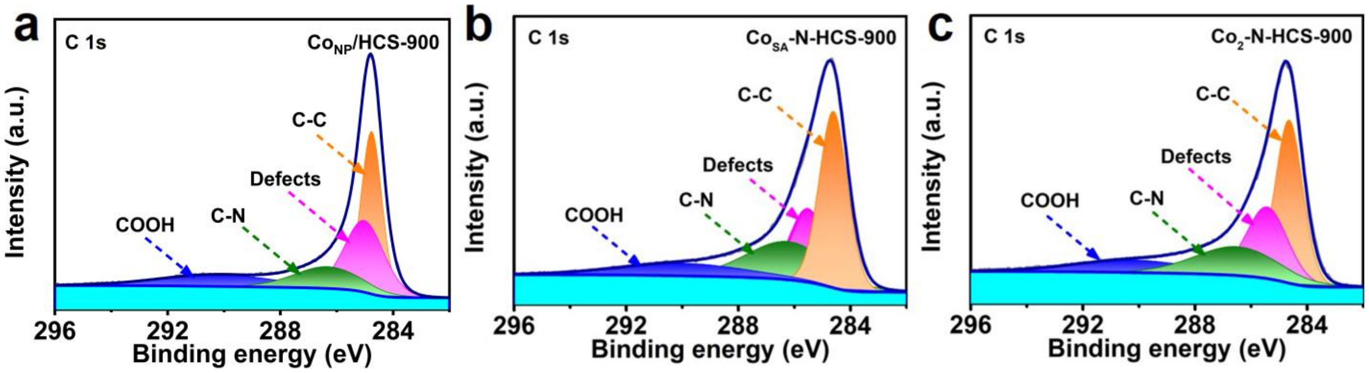

Figure S44. C 1*s* high-resolution XPS spectra of (a) Co_{NP}/HCS-900, (b) Co_{SA}-N-HCS-900, and (c) Co_2-N-HCS-900.

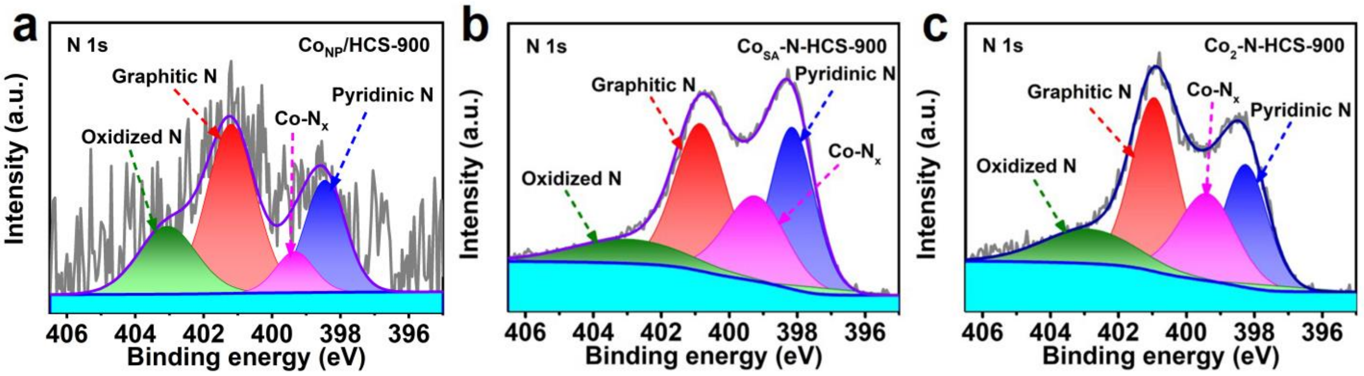

Figure S45. N 1*s* high-resolution XPS spectra of (a) Co$_{NP}$/HCS-900, (b) Co$_{SA}$-N-HCS-900, and (c) Co$_2$-N-HCS-900.

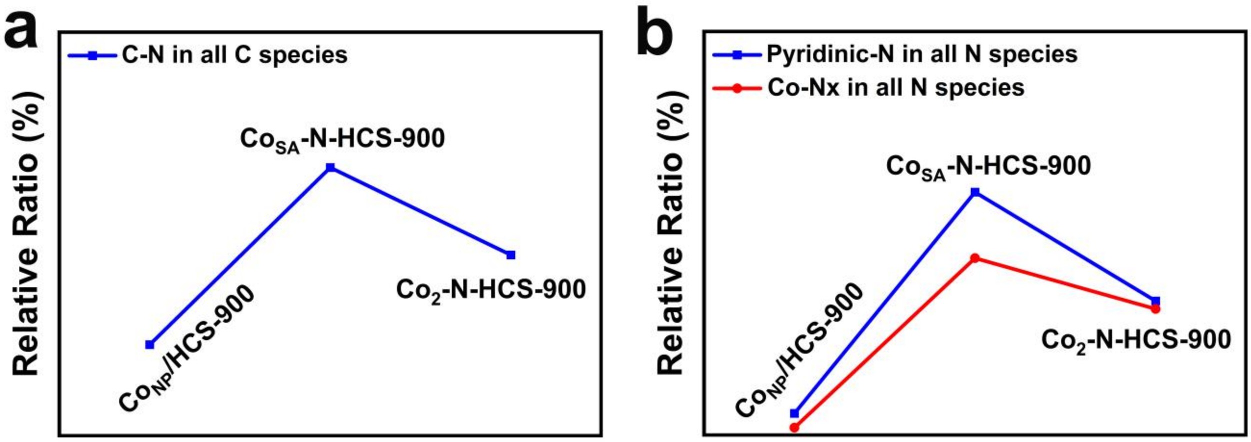

Figure S46. The tendency of (a) C-N bond, (b) pyridinic-N and $Co-N_x$ from Co_{NP}/HCS-900 to Co_{SA}-N-HCS-900 and Co_2-N-HCS-900.

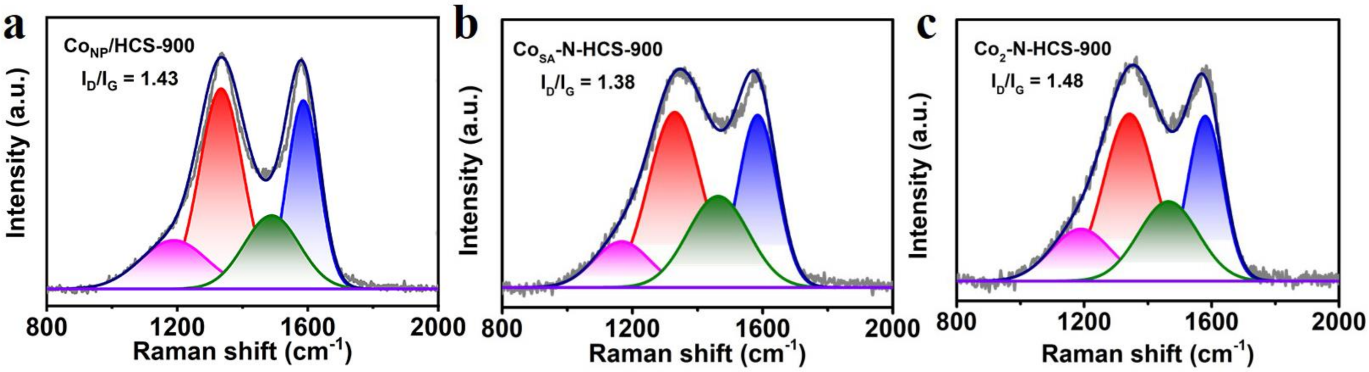

Figure S47. Raman spectra for the (a) Co_{NP}/HCS-900, (b) Co_{SA}-N-HCS-900, and (c) Co_2-N-HCS-900.

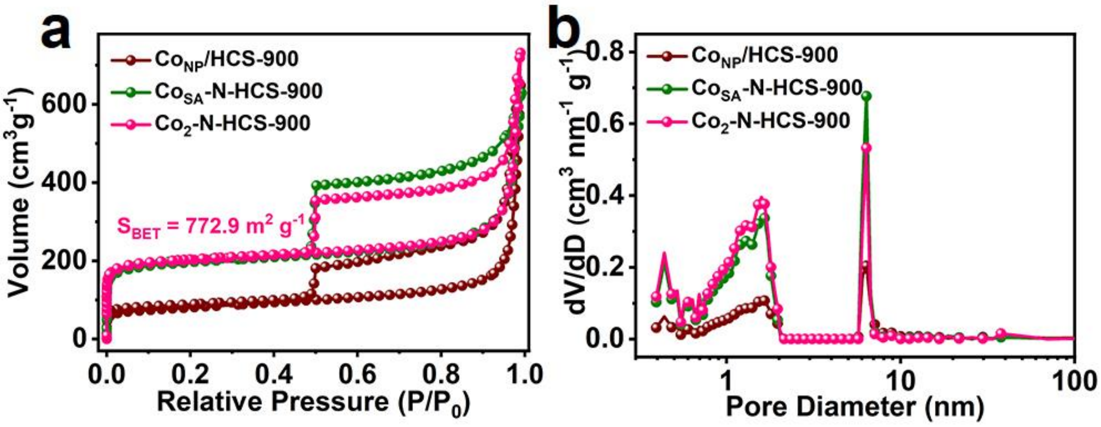

Figure S48. (a) Nitrogen adsorption-desorption isotherms and (b) pore size distribution for the Co_{NP}/HCS-900, Co_{SA}-N-HCS-900, and Co_2-N-HCS-900.

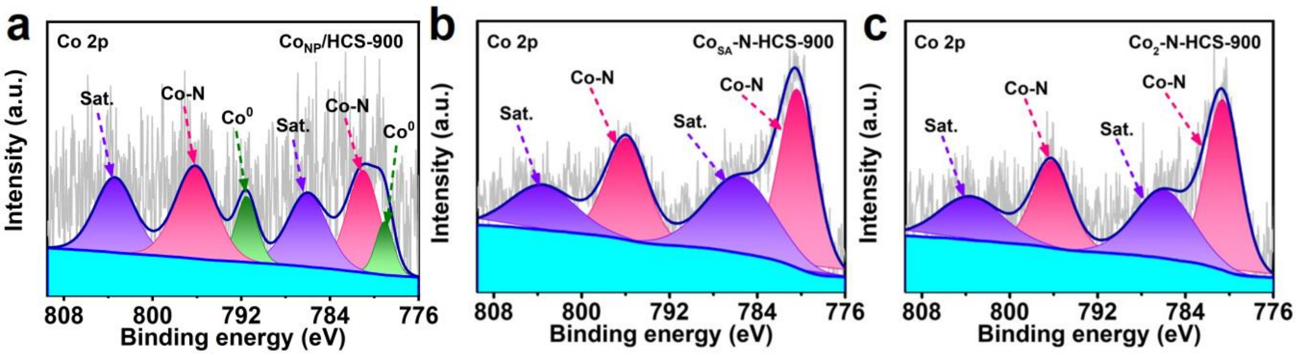

Figure S49. Co 2*p* high-resolution XPS spectra of (a) Co_{NP}/HCS-900, (b) Co_{SA}-N-HCS-900, and (c) Co_2-N-HCS-900.

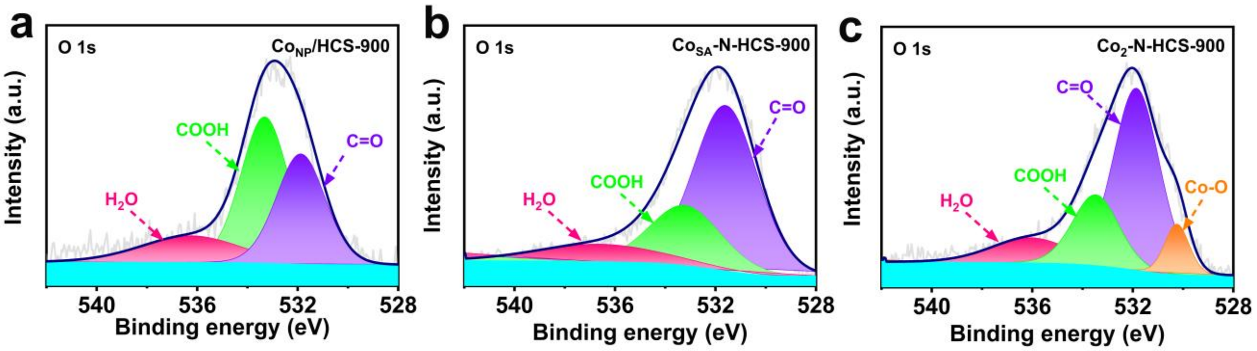

Figure S50. O 1*s* high-resolution XPS spectra of (a) Co$_{NP}$/HCS-900, (b) Co$_{SA}$-N-HCS-900, and (c) Co_2-N-HCS-900.

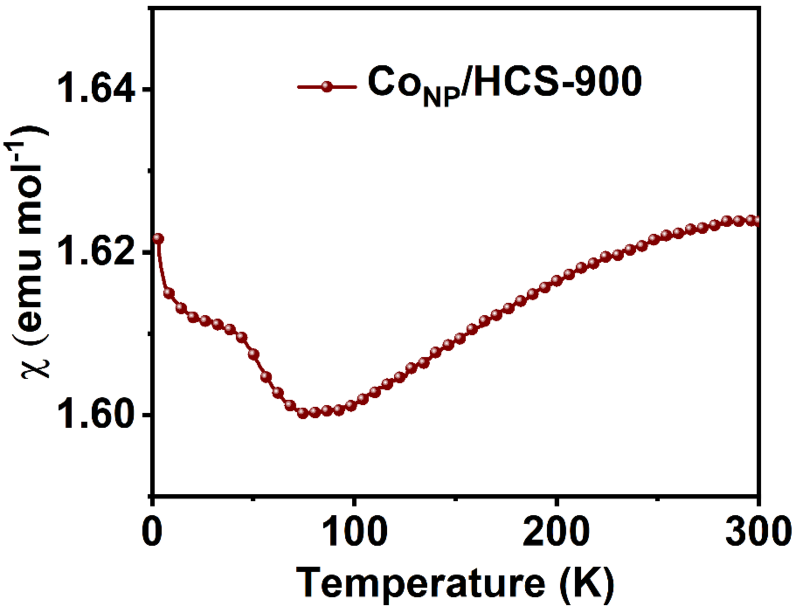

Figure S51. M-T curve of the Co_{NP}/HCS-900.

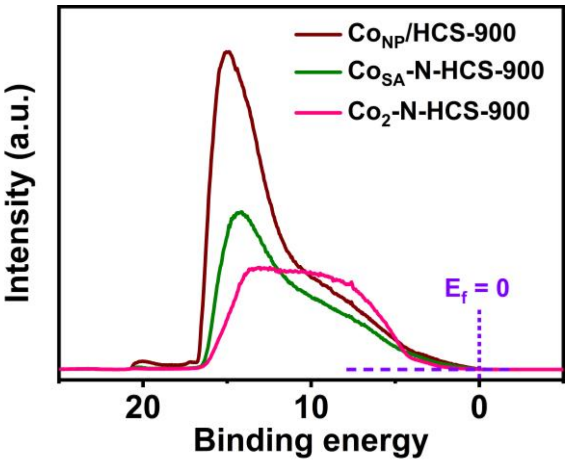

Figure S52. UPS for the Co$_{NP}$/HCS-900, Co$_{SA}$-N-HCS-900, and Co$_2$-N-HCS-900.

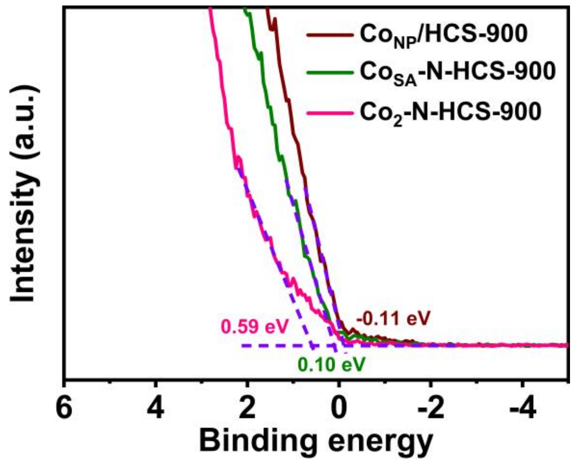

Figure S53. The valence-band spectrum for the Co_{NP}/HCS-900, Co_{SA}-N-HCS-900, and Co_2-N-HCS-900

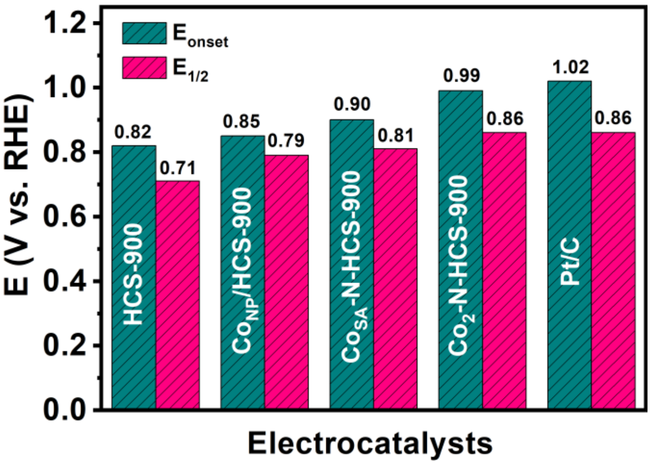

Figure S54. The corresponding E_{onset} and $E_{1/2}$ for the HCS-900, Co_{NP}/HCS-900, Co_{SA}-N-HCS-900, Co_2-N-HCS-900, and commercial Pt/C.

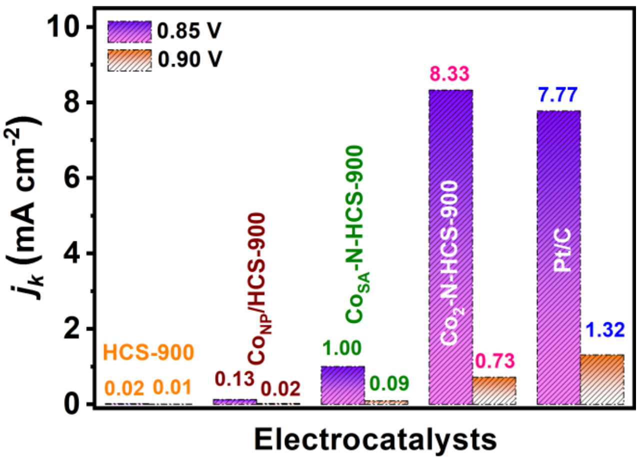

Figure S55. The corresponding j_k for the HCS-900, Co_{NP}/HCS-900, Co_{SA}-N-HCS-900, Co_2-N-HCS-900, and commercial Pt/C at 0.90 V and 0.85 V.

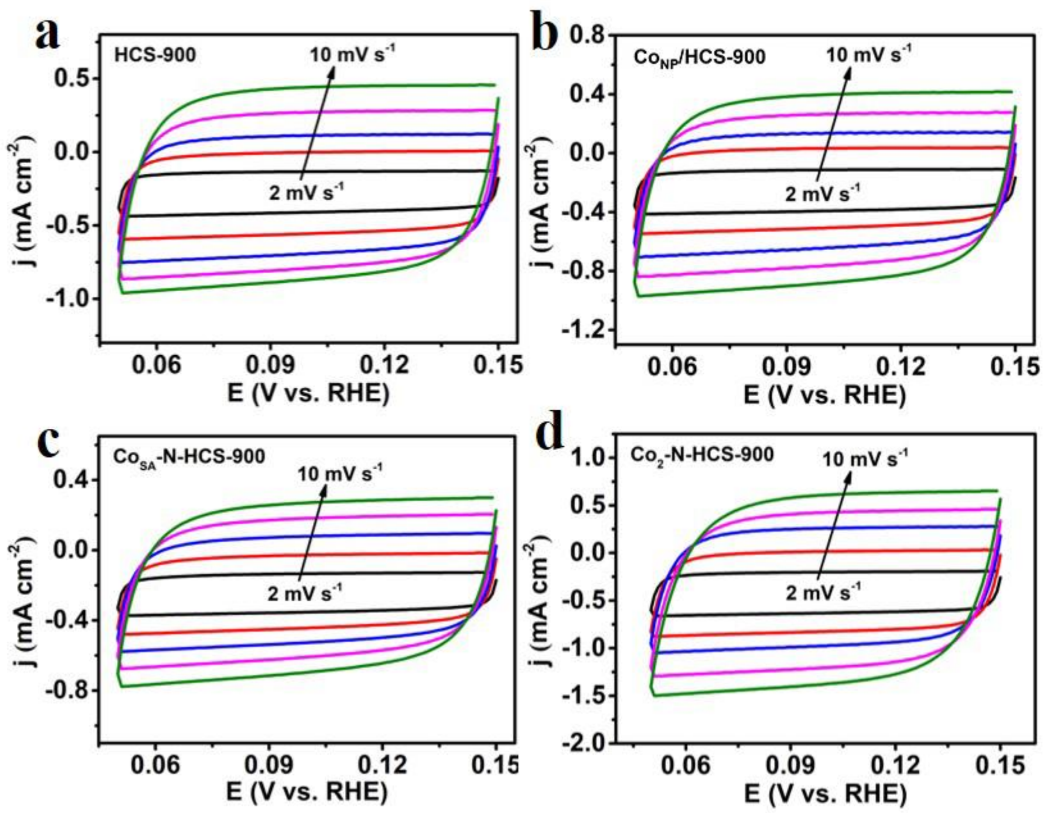

Figure S56. Cyclic voltammetry (CV) curves for (a) HCS-900, (b) Co_{NP}/HCS-900, (c) Co_{SA}-N-HCS-900, and (d) Co_2-N-HCS-900 in O_2-saturated 0.1 M KOH solution.

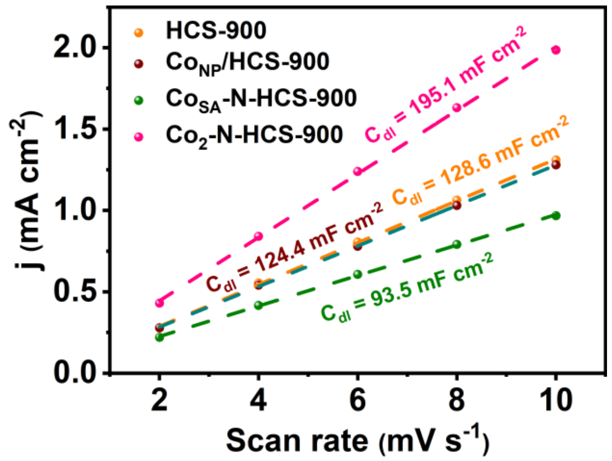

Figure S57. The value of C_{dl} for the HCS-900, Co_{NP}/HCS-900, Co_{SA}-N-HCS-900, and Co_2-N-HCS-900 in O_2-saturated 0.1 M KOH solution.

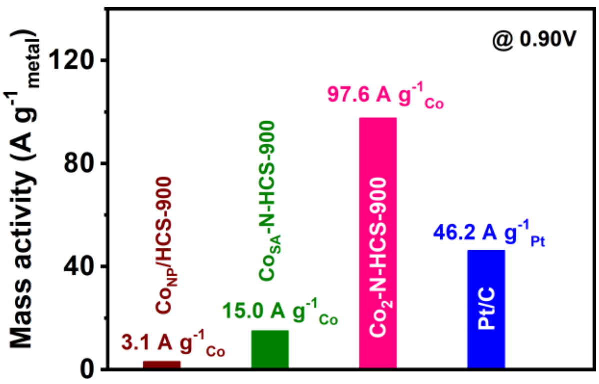

Figure. S58. Mass activity toward ORR for the Co_{NP}/HCS-900, Co_{SA}-N-HCS-900, Co_2-N-HCS-900 and commercial Pt/C.

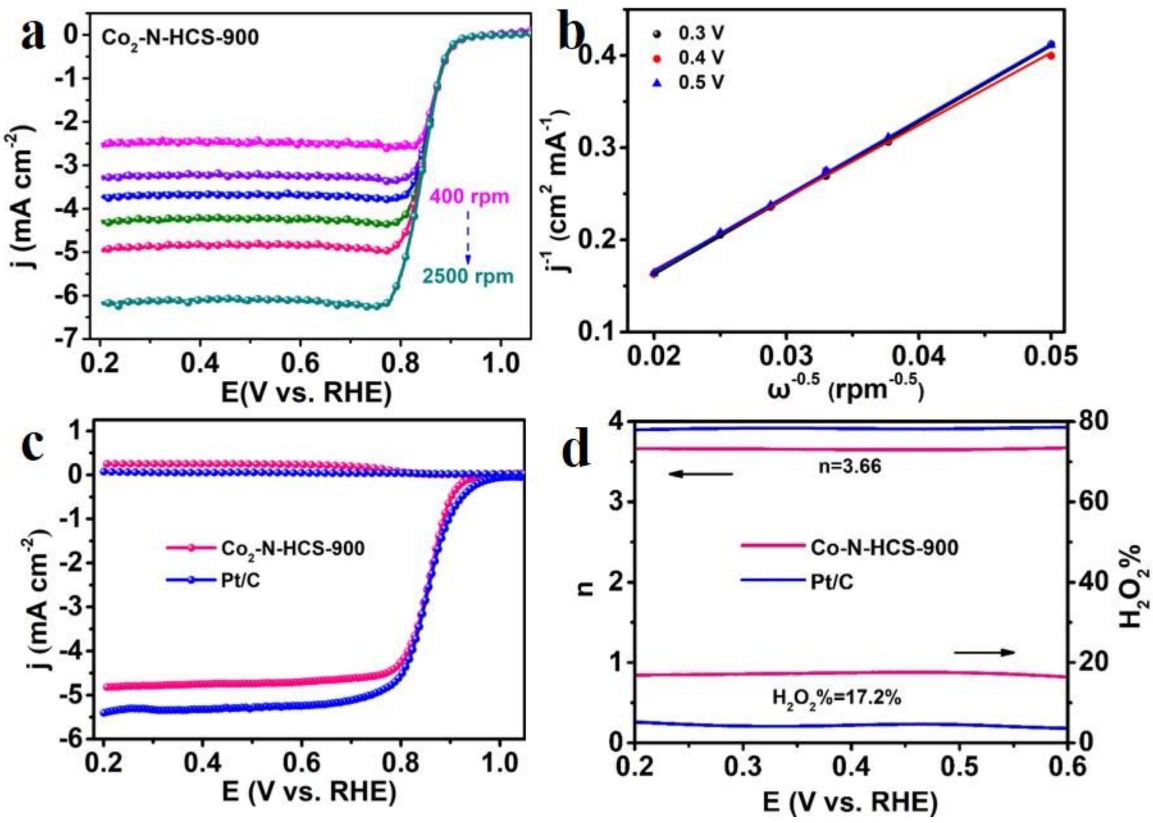

Figure S59. (a) LSV curves of Co_2-N-HCS-900 at different rotation rates, (b) K-L plots, (c) RRDE curves, and (d) the HO_2^- yield and n in O_2-saturated 0.1 M KOH solution.

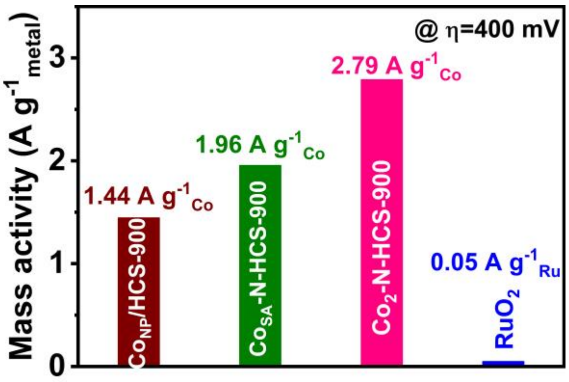

Figure S60. Mass activity toward OER for the Co_{NP}/HCS-900, Co_{SA}-N-HCS-900, Co_2-N-HCS-900 and commercial RuO_2.

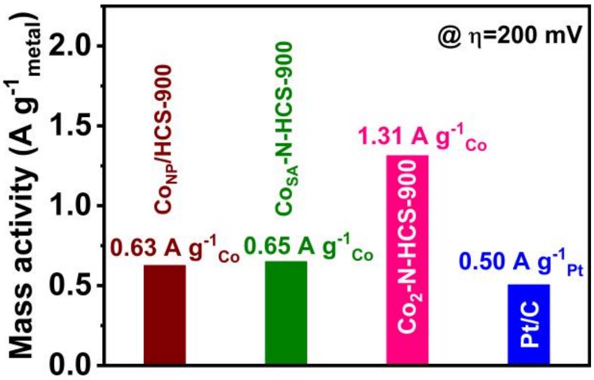

Figure S61. Mass activity toward HER for the Co_{NP}/HCS-900, Co_{SA}-N-HCS-900, Co_2-N-HCS-900 and commercial Pt/C.

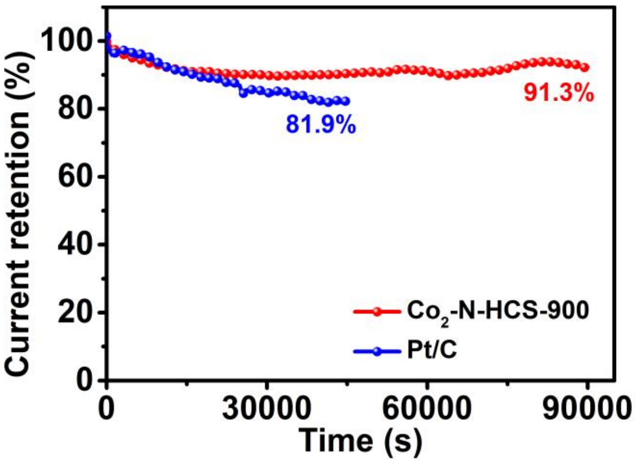

Figure S62. Relative current-density-time curves of Co_2-N-HCS-900 and Pt/C at 0.7 V with a rotation speed of 200 rpm in O_2-saturated 0.1 M KOH solution.

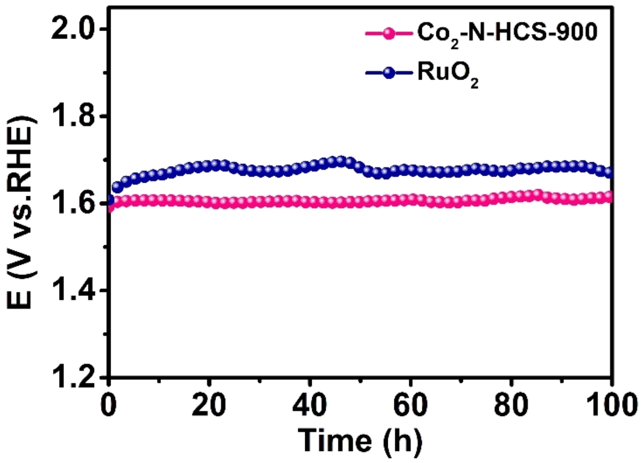

Figure S63. Durability tests for OER at 10 mA cm^{-2} in 1 M KOH solution.

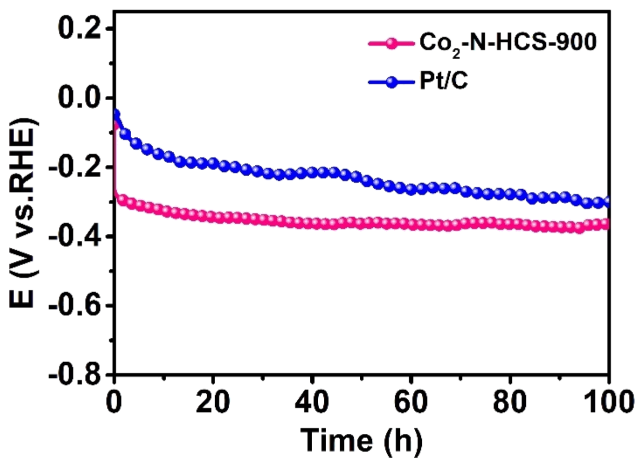

Figure S64. Durability tests for HER at 10 mA cm^{-2} in 1 M KOH solution.

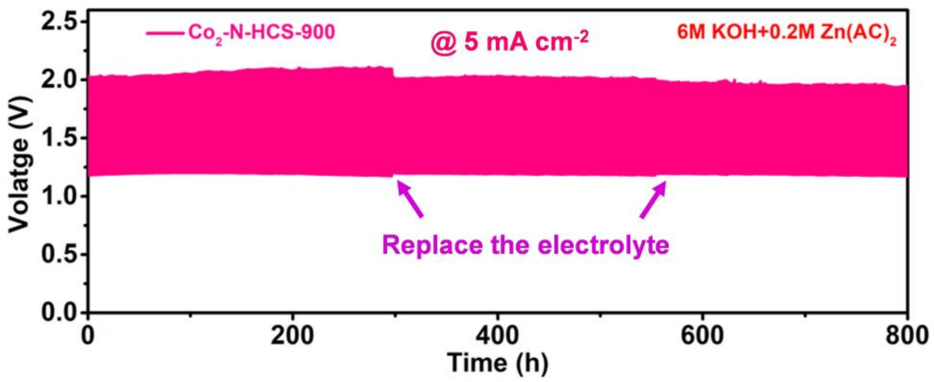

Figure S65. Galvanostatic discharge-charge cycling curves for ZABs driven by Co_2-N-HCS-900 at current density of 5 mA cm^{-2}.

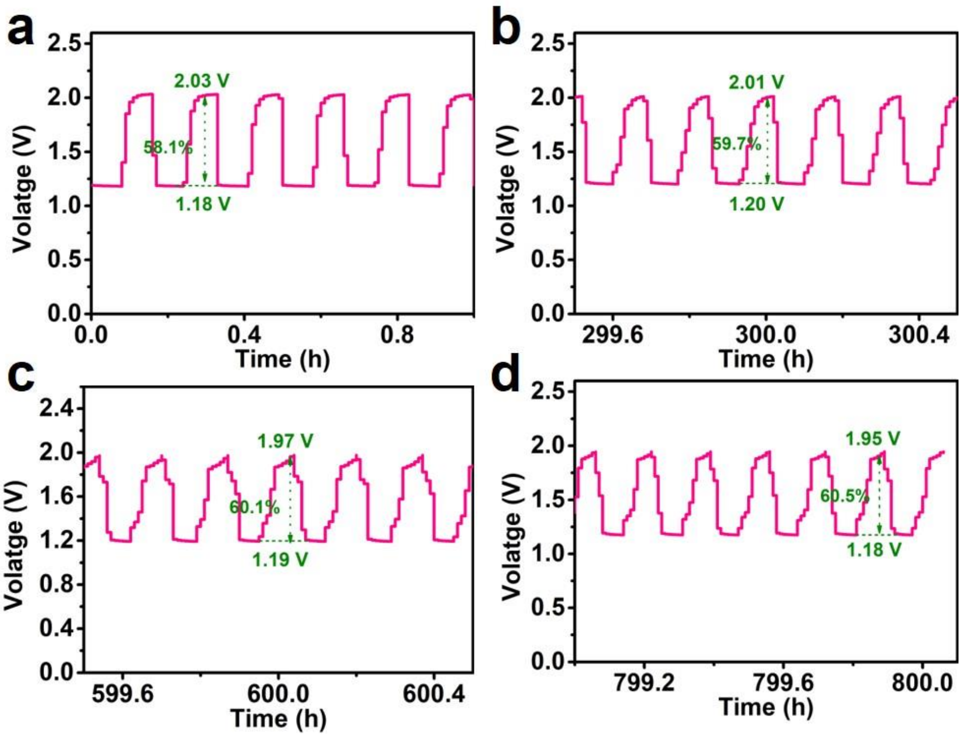

Figure S66. Galvanostatic discharge-charge cycling curves for ZABs using Co_2-N-HCS-900 as the air cathode at (a) 0-1 h, (b) 299-300 h, (c) 599-600 h, and (d) 799-800 h.

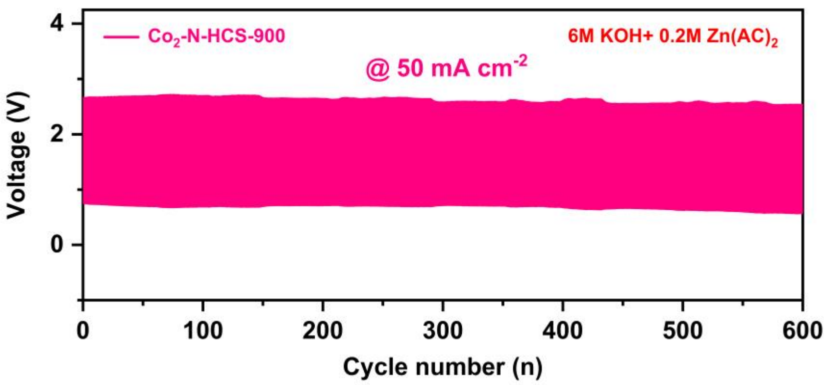

Figure S67. Galvanostatic discharge-charge cycling curves for ZABs driven by Co_2-N-HCS-900 at current density of 50 mA cm^{-2}.

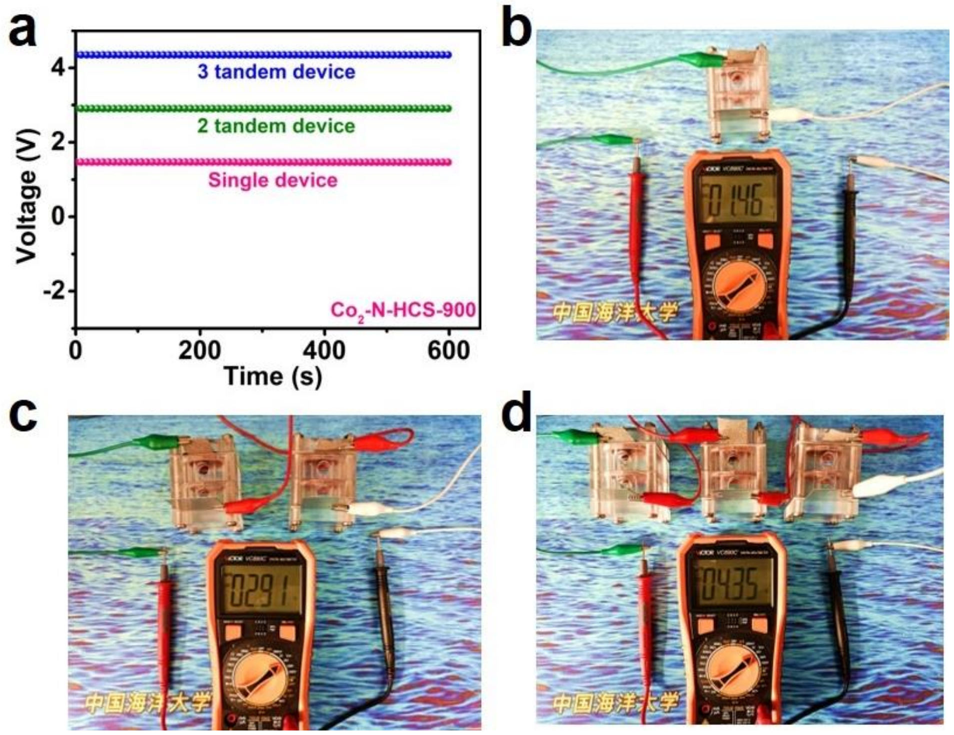

Figure S68. (a) Open-circuit potential plots of one or two or three tandem Zn-air batteries driven by Co_2-N-HCS-900, and (b-d) Photograph of one or two or three tandem ZABs driven by Co_2-N-HCS-900 displaying a measured open-circuit voltage of ~1.46, 2.91, and 4.35 V.

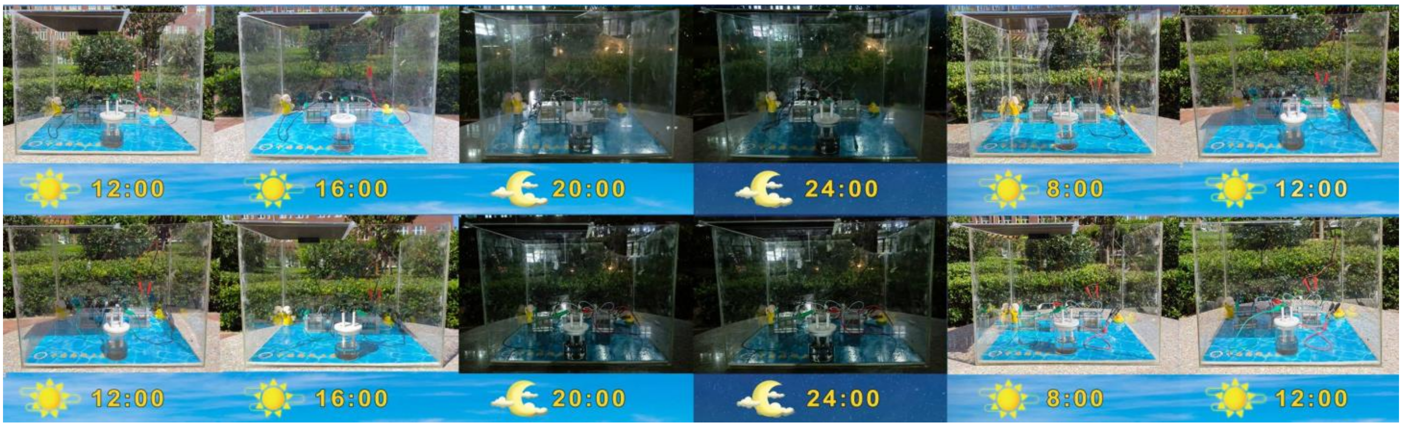

Figure S69. Photograph of series WSS driven by Co_2-N-HCS-900 during the day and night over 48 h.

Table S1. EXAFS fitting parameters at the Co K-edge for various samples (S_0^2= 0.776).

Sample	Shell	N^a	R(Å)^b	σ^2(Å^2)^c	ΔE_0(eV)^d	R factor
Co foil	Co-Co	12	2.49	0.0062	7.1	0.0004
CoO	Co-O	6.0	2.06	0.0101	-3.6	0.0004
	Co-Co	12.5	2.99	0.0095		
	Co-O	6.2	3.66	0.0101		
CoPc	Co-N	4.0	1.91	0.0016	6.8	0.0025
Co_{SA}-N-HCS-900	Co-N/O	4.0	1.93	0.0113	-6.9	0.0068
	Co-Co	0.1	2.23	0.0117		
	Co-C	2.1	2.84	0.0132		
Co_2-N-HCS-900	Co-N/O	3.9	1.92	0.0126	-10.8	0.0033
	Co-Co	0.8	2.24	0.0074		
	Co-C	2.4	2.83	0.0137		

$^a N$: coordination numbers; $^b R$: bond distance; $^c\sigma^2$: Debye-Waller factors; ^{d} ΔE_0: the inner potential correction. R factor: goodness of fit. S_0^2 was set to 0.776 for Co, according to the experimental EXAFS fit of Co foil references by fixing CN as the known crystallographic value.

Table S2. The XANES and EXAFS fitting parameters at the Co K-edge of the possible structures from model 1 to model 10 for the Co_{SA}-N-HCS-900.

Model	XANES (S_0^2)	EXAFS (R-factor)
1	10.73	0.1370
2	3.42	0.0151
3	5.34	0.0403
4	12.76	0.1071
5	2.40	0.0737
6	1.51	0.1543
7	7.93	0.0195
8	6.39	0.0192
9	0.46	0.0068
10	4.68	0.0108

Table S3. The XANES and EXAFS fitting parameters at the Co K-edge of the possible structures from model 1 to model 10 for the Co_2-N-HCS-900.

Model	XANES (S_0^2)	EXAFS (R-factor)
1	13.50	0.1603
2	0.39	0.0033
3	6.34	0.0441
4	13.07	0.1184
5	3.31	0.0810
6	2.42	0.1825
7	7.73	0.0267
8	7.30	0.0254
9	3.58	0.0389
10	5.20	0.0128

Table S4. Comparison of alkaline ORR performances between Co_2-N-HCS-900 and other M-N-C materials in the literatures.

Catalysts	$E_{1/2}$/VRHE	Tafel/mV dec^{-1}	Electrolyte	Reference
Co_2-N-HCS-900	0.86	48	0.1 M KOH	This work
Sb1/NG(O)	0.86	54	0.1 M KOH	*Angew. Chem. Int. Ed.* ***61***, 202202200 (2022)
PSTA-Co-1000	0.878		0.1 M KOH	*Angew. Chem. Int. Ed.* **132**, 14747-14754 (2020)
Fe/Co-Nx-C	0.86	53.6	0.1 M KOH	*Small,* **16**, 2000742 (2020)
Zn-B/N-C	0.886	50	0.1 M KOH	*Angew. Chem. Int. Ed.* **133**, 183-187 (2021)
Zn-N-C-1	0.873	103	0.1 M KOH	*Angew. Chem. Int. Ed.* **58**, 7035-7039 (2019)
Cu/Zn@NC	0.83	54.8	0.1 M KOH	*Angew. Chem. Int. Ed.* **60**, 14005-14012 (2021)
Co-Te DASs/N-C	0.852	62.3	0.1 M KOH	*Small* **18**, 2201974 (2022)
$FeCoN_X/C$	0.86	70	0.1 M KOH	*J. Am. Chem. Soc.* **141**, 17763-17770 (2019)
PtNPC-0.5	0.87		0.1 M KOH	*Angew. Chem. Int. Ed.* **60**, 21911-21917 (2021)
Cu-N-C	0.83	37	0.1 M KOH	*J. Am.Chem. Soc.* **143**, 14530-14539 (2021)
MS-CoSA-N-C-800°C	0.86	88.7	0.1 M KOH	*ACS Nano* **16**, 11944-11956 (2022)
(Zn, Cu)-NC	0.88	87.1	0.1 M KOH	*Adv. Funct. Mater.* **32**, 2203471 (2022)
CoN_4/NG	0.88	59	0.1 M KOH	*Nano Energy* **50**, 691-698 (2018)
Co-N-C@F127	0.84		0.1 M KOH	*Energy Environ. Sci.* **12**, 250-260 (2019)
NiSAs-Pd@NC (2 : 1)	0.84	55	0.1 M KOH	*J. Mater. Chem. A* **10**, 6086-6095 (2022)

Table S5. Comparison of alkaline OER performances between Co_2-N-HCS-900 and other M-N-C materials in the literatures.

Catalysts	η_{10} / mV	Tafel/mV dec^{-1}	Electrolyte	Reference
Co_2-N-HCS-900	333	97.1	1 M KOH	This work
CoFe-N-C	360	67.7	1 M KOH	*Nano Lett.* **22**, 3392-3399 (2022)
IrFe-N-C	350	43	1 M KOH	*ACS Catal.* **12**, 9397-9409 (2022)
NCAG/Fe-Cu	380		1 M KOH	*Angew. Chem. Int. Ed.* **61**, 202201007 (2022)
FeCo SAs@ Co/N-GC	290	56.6	1 M KOH	*ACS Nano* **15**, 14683-14696 (2021)
FeCo-DACs/NC	370	82.7	1 M KOH	*Adv. Mater.* **34**, 2107421 (2022)
Fe_1Co_3-NC-1100	349	99.93	1 M KOH	*ACS Catal.* **12**, 1216-1227 (2022)
Fe,Co,N-C	410	76	1 M KOH	*ACS Nano* **16**, 7890-7903 (2022)
CoSA/NCs	303	76	1 M KOH	*Appl. Catal. B- Environ.* **316**, 121674 (2022)
CoSA/N-HCS	306	38.1	1 M KOH	*Adv. Energy Mater.* **10**, 2002896 (2020)
NiFe-DASC	310	45	1 M KOH	*Nat. Commun.* **12**, 1-11 (2021)
Fe-N/S-CNT-GR	370		0.1 M KOH	*ACS Catal* **12**, 7994-8006 (2022)
CoN_4/NG	380	81	0.1 M KOH	*Nano Energy* **50**, 691-698 (2018)
Ni SAs-Pd@NC (2 : 1)	380	79	0.1 M KOH	*J. Mater. Chem. A* **10**, 6086-6095 (2022)
MoS_2@Fe-N-C	360	98	0.1 M KOH	*P. Natl. Acad. Sci. USA* **118**, 2110036118 (2021)

Table S6. Comparison of alkaline HER performances between Co_2-N-HCS-900 and other M-N-C materials in the literatures.

Catalysts	η_{10} / mV	Tafel/mV dec^{-1}	Electrolyte	Reference
Co_2-N-HCS-900	166	83.9	1 M KOH	This work
Co_{SA}/N, S-HCS	165	96	1 M KOH	*Adv. Energy Mater.* **10**, 2002896 (2020)
Ru/Co-N-C-800 °C	19	27.8	1 M KOH	*Adv. Mater.* **34**, 2110103 (2022)
Ru-1.0	13	25.3	1 M KOH	*Adv. Energy Mater.* **11**, 2101242 (2021)
S-Co/N/C	121	47	1 M KOH	*ACS Catal.* **11**, 4498-4509 (2021)
$RuSACoFe_2$/G	164	116	1 M KOH	*Energy Environ. Sci.* **13**, 5152-5164 (2020)
CuPor-RuN_3	114		1 M KOH	*Adv. Funct. Mater.* **31**, 2107290 (2021)
NiCo-SAD-NC	61	55	1 M KOH	*Nat. Commun.* **12**, 6766 (2021)
Ni-N-C	30.8	32	1 M KOH	*J. Am. Chem. Soc.* **138**, 14546 (2016)
Co-NMGO	146	95	1 M KOH	*Adv. Energy Mater.* **11**, 2101619 (2021)
Ru@Co/N-CNTs-2	48	33	1 M KOH	*ACS Sustain. Chem. Eng.* **8**, 9136 (2020)
CoFeN-NCNTs//CCM	151	130	1 M KOH	*Adv. Funct. Mater.* **32**, 2107608 (2021)
Co-Te DASs/N-C	217	76.8	1 M KOH	*Small* **18**, 2201974 (2022)
Co/CNFs (1000)	190	56	1 M KOH	*Adv. Mater.* **31**, 1808043 (2019)
CoSAs-MoS_2/TiN NRs	187	53.5	1 M KOH	*Adv. Funct. Mater.* **31**, 2100233 (2021)
Co-BM-C	126	63	1 M KOH	*Chem. Eng. J.* **433**, 134089 (2022)

Table S7. Comparison of trifunctional electrocatalytic performance between Co_2-N-HCS-900 and other M-N-C materials in the literatures.

**Catalysts**	**$E_{1/2}$ of ORR**	**η_{10} / mV of OER**	**η_{10} / mV of HER**	**Reference**
Co_2-N-HCS-900	0.86	333	166	This work
Co/CNFs (1000)	0.89	320	190	*Adv. Mater.* **31**, 1808043 (2019)
TSAs	0.88	393	94	*Adv. Energy Mater.* **13**, 2203150 (2023)
Co_{SA}/N, S-HCS	0.85	306	165	*Adv. Energy Mater.* **10**, 2002896 (2020)
Co-N, P-HCS	0.89	320	164	*Adv. Mater.* **34**, 2204021 (2022)
CF-NG-Co	0.88	400	212	*J. Mater. Chem. A* **6**, 489-497 (2018)
Co-NC@CC	0.81	240	73	*Adv. Funct. Mater.* **31**, 2009853 (2021)
Mo-N/C@MoS_2	0.81	390	117	*Adv. Funct. Mater.* **27**, 1702300 (2017)
Fe_3C-Co/NC	0.88	340	238	*Adv. Funct. Mater.* **29**, 1901949 (2019)
Co_2P/CoNPC	0.84	326	130	*Adv. Mater.* **32**, 2003649 (2020)

Table S8. Comparison of Zn-air batteries performances between Co_2-N-HCS-900 and other M-N-C materials in the literatures.

**Catalysts**	**Powder density ($mW\ cm^{-2}$)**	**Specific capacity ($mAh\ g^{-1}$)**	**Electrolyte**	**Reference**
Co_2-N-HCS-900	188.2	754.2	6 M KOH+0.2 M $Zn(OAC)_2$	This work
Pt+RuO_2	129.3	612.5	6 M KOH+0.2 M $Zn(OAC)_2$	This work
Co_{SA}/N, S-HCS	173.1	781.1	6 M KOH+0.2 M $Zn(OAC)_2$	*Adv. Energy Mater.* **10**, 2002896 (2020)
Fe,Mn/N-C	160.8	902 @ 5 mA cm^{-2}	6 M KOH+ 0.2 M ZnCl2	*Nat. Commun.* **12**, 1734 (2021)
CoFeN-NCNTs//CCM	145	778.4	6 M KOH+0.2 M $Zn(OAC)_2$	*Adv. Funct. Mater.* **32**, 2107608 (2021)
FeCo-DACs/NC	175	--	6 M KOH+0.2 M $Zn(OAC)_2$	*Adv. Mater.* **34**, 2107421 (2022)
FeNi SAs/NC	42.22	779.4	6 M KOH+0.2 M $Zn(OAC)_2$	*Adv. Energy Mater.* **11**, 2101242 (2021)
Fe-N/P-C-700	133.2	723@100 mA cm^{-2}	6 M KOH	*J. Am. Chem. Soc.* **142**, 2404-2412 (2020)
FeCo SAs@Co/N-GC	207	741@5 mA cm^{-2}	6 M KOH	*ACS Nano* **15**, 14683-14696 (2021)
o-MQFe-10: 20: 5	158.2	807.8	6 M KOH+0.2 M $Zn(OAC)_2$	*Angew. Chem. Int. Ed.* 202117617 (2022)
FeN4-Te_n	183	773@100 mAcm^{-2}	6 M KOH+0.2 M $Zn(OAC)_2$	*Adv. Mater.* **34**, 2202714 (2022)
D-Fe SAC	215	215	6 M KOH+0.2 M $Zn(OAC)_2$	*Angew. Chem. Int. Ed.* **60**, 22722-22728 (2021)
